# H3K27M cooperates with ASCL1 to maintain progenitor states and drive gliomagenesis

**DOI:** 10.1038/s41467-026-78138-z

**Published:** 2026-10-10

**Authors:** Alan L. Jiao, Abolfazl Maghrouni, Arantxa Gutierrez, Alva Annett, Cherubin Manokaran, Tingyu Wen, Niklas Grassl, Jonathan H. Chan, Sylvain Nguyen, Berna Bou-Tayeh, Wajih Jawhar, François Le Dily, Claudia L. Kleinman, Luciano Di Croce, Nada Jabado, Yang Shi

**Affiliations:** 1 Ludwig Institute for Cancer Research, Nuffield Department of Medicine, University of Oxford, Oxford, UK; 2 Department of Human Genetics, McGill University, Montreal, QC Canada; 3 Research Institute of the McGill University Health Centre, Montreal, QC Canada; 4 Centre for Genomic Regulation (CRG), Barcelona Institute of Science and Technology (BIST), Barcelona, Spain; 5 Department of Neurology, Medical Faculty Mannheim, MCTN, Heidelberg University, Mannheim, Germany; 6 Radcliffe Department of Medicine, Division of Cardiovascular Medicine, University of Oxford, Oxford, UK; 7 Centre for Human Genetics, University of Oxford, Oxford, UK; 8 Division of Experimental Medicine, Department of Medicine, McGill University, Montreal, QC Canada; 9 The Lady Davis Institute, Jewish General Hospital, Montreal, QC Canada; 10 Universitat Pompeu Fabra (UPF), Barcelona, Spain; 11 ICREA, Pg. Lluis Companys 23, Barcelona, Spain; 12 Department of Pediatrics, McGill University, and The Research Institute of the McGill University Health Centre, Montreal, QC Canada

**Keywords:** Epigenetics, Histone post-translational modifications, Cancer stem cells, CNS cancer

## Abstract

Histone 3 lysine 27-to-methionine substitutions (H3K27M) drive aggressive diffuse midline gliomas by blocking differentiation of radial glial progenitor cells committed to oligodendrogenesis. We establish prolonged differentiation as a powerful tool to uncover regulators of glioma cell states and demonstrate that the pioneer transcription factor (TF) ASCL1 is a key mediator of H3K27M-driven plasticity and tumorigenesis. H3K27M prevents Polycomb silencing of *ASCL1*, co-opting this TF to maintain oligodendroglial progenitor states. Consistently, ASCL1 loss drastically impairs tumor formation in vivo. Mechanistically, ASCL1 synergizes with H3K27M to redistribute the chromatin remodeler BRG1 to regulatory elements co-enriched for ASCL1 and SOX motifs, promoting expression of stem and neural progenitor genes at the expense of mesenchymal genes in DMGs. ASCL1 loss acts as a “fire exit”, enabling alternative differentiation despite H3K27M, underscoring the therapeutic potential of targeting stalled differentiation and tumor plasticity in histone-mutant gliomas and cancers with a stem cell hierarchy.

## Abstract (web summary)

Histone 3 lysine 27-to-methionine substitutions (H3K27M) cause aggressive DMGs by preventing radial glial progenitor cell differentiation into oligodendrocytes. Here, the authors show that the transcription factor ASCL1 works with K27M to guide BRG1, maintaining cells in an oligodendrocyte precursor state.

## Introduction

Phenotypic plasticity – defined here as the ability of a cell population to adopt multiple states – contributes to tumor heterogeneity and creates a significant obstacle to effective cancer treatment^1–4^. The chromatin state is a key determinant of plasticity, and is heavily remodeled during cell differentiation by the cooperative functions of lineage-determining transcription factors (TFs), chromatin remodelers, and chromatin modifying enzymes^5,6^. This epigenetic regulation underpins the multiple, interconvertible cell states identified in numerous malignancies including gliomas^7–9^, neuroblastomas^10^, lung^11,12^, breast^13,14^, and prostate^15,16^ cancers, highlighting aberrant phenotypic plasticity as a general hallmark of cancer^6,17^.

Diffuse midline gliomas (DMGs) are incurable childhood brain tumors primarily driven by somatic oncogenic, gain-of-function, lysine-27-to-methionine mutations in noncanonical and canonical H3 variants (hereafter referred to as “K27M”)^18–21^. These mutations, present in ~80% of DMGs^22^, arise in radial glial/oligodendroglial progenitor cells (RGC/OPCs)^23–26^, arresting them in a proliferative, oligodendrocyte precursor cell (OPC)-like state^27–31^. The dominant inhibition of Polycomb Repressive Complex 2 (PRC2) activity and spreading by K27M is conserved across species^32–36^, reducing global H3K27 trimethylation (H3K27me3) despite limited effects on PRC2 recruitment^34,37,38^. H3.3K27M was also recently shown to suppress mitotic H3.3Ser31 phosphorylation^39,40^. Abnormal self-renewing subpopulations of RGC/OPC-like cells, which are lost upon removal of K27M, are capable of tumor initiation and maintenance^41^. They co-exist with other cellular populations including non-cycling, astrocytic/mesenchymal (MES)-like cells^31,36,41,42^, suggesting a degree of cellular plasticity in oncohistone-driven DMGs. This resembles the co-existing cell states observed in adult glioblastoma, where hypoxia and the tumor microenvironment contribute to drive cell state transitions^17,43^. However, the mechanisms underlying plasticity in DMGs and potential for therapeutic exploitation remain largely unknown.

In this study, we demonstrate that phenotypic plasticity in H3K27M-driven DMGs critically depends on the lineage-specific pioneer TF ASCL1. Using a long-term differentiation approach, we uncover the dynamic interplay between lineage-determining TF activity and epigenetic dysregulation that drives plasticity and tumorigenesis in DMG. We show that ASCL1 collaborates with K27M to direct BRG1 deposition, maintaining cells in an OPC state. Our findings highlight ASCL1’s role in brain midline glial progenitors and OPCs, where it actively inhibits astro-mesenchymal and ependymal differentiation to preserve progenitors along an OPC trajectory. These insights offer promising therapeutic opportunities for pediatric gliomas, and our approach can be extended to other cancers with epigenetic abnormalities, identifying inherent targetable vulnerabilities arising from cellular plasticity.

## Results

### ASCL1 is a critical tumorigenic dependency in K27M-mutant DMGs

**Fig. 1:**
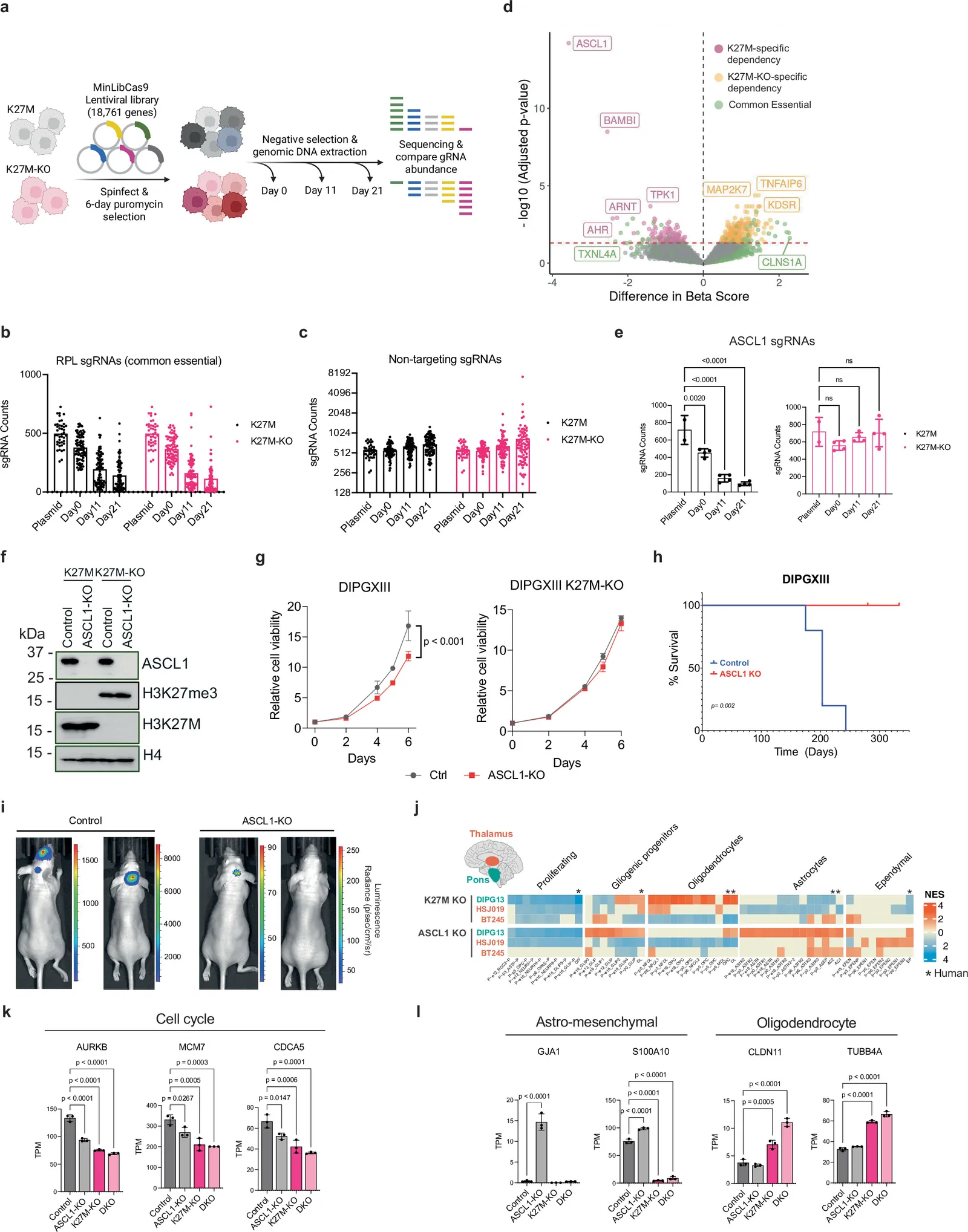
Enhanced ASCL1 dependency in K27M-mutant DMGs. **a** Schematic of CRISPR screen in isogenic DIPG-XIII lines. Created in BioRender. Jiao, A. (2026) https://BioRender.com/m6t1no9. Counts of guide RNAs (gRNAs) targeting common essential genes (**b**) and non-targeting scrambled controls (**c**). Each dot represents a single gRNA from one of two biological replicates. **d** Volcano plot of statistical significance (y-axis) and effect size (x-axis) for the difference in gene essentiality (MAGeCK-derived beta scores) between DIPGXIII K27M and K27M-KO lines. Negative differences in beta scores indicate K27M-specific essentiality. Top 4–6 genes as ranked by absolute difference in beta-values or statistical significance (two-sided Wald test) are labeled. Data are from the CRISPR screen shown in (**a**) performed as *n* = 2 independent experiments. Dashed red line indicates adjusted *p*-value < 0.05. **e** *ASCL1* gRNA counts at different time points of the screen. Each dot represents one of two *ASCL1*-targeting gRNAs in one of two biological replicates. “Plasmid” indicates gRNA counts in the starting gRNA plasmid pool before transfection. Data are shown as mean ± SD. ns not significant, two-way ANOVA. **f** Western blot confirmation of ASCL1 deletion in K27M and K27M-KO DIPG-XIII cells. Control cells carry non-targeting control sgRNAs. Data are representative of three independent experiments. **g** Cell proliferation as measured by CellTiterGlo following *ASCL1*-KO in either K27M or K27M-KO cells. Data are shown as mean ± SD and are representative of *n* = 3 independent experiments. Statistic: two-way ANOVA. **h** Kaplan–Meier survival curves of orthotopic xenograft mouse models with Control or ASCL1-KO DIPG-XIII injected cells into the pons (*n* = 5 per line; log-rank test). **i** Representative bioluminescent (BLI) images of mice xenografted with Control or ASCL1-KO DIPG-XIII-luciferase cells 99 days post-injection. **j** Heatmap of normalized enrichment scores (NES) of significantly enriched (adjusted *p* value < 0.05) developmental mouse pons cell types and human thalamus cell types by gene set enrichment analysis (GSEA) from RNA-seq in DIPG-XIII, HSJ019 and BT245 cells (*n* = 3 biological replicates). Schematic created in Adobe Illustrator. **k**, **l** Gene expression of cell cycle, oligodendrocyte, and astro-mesenchymal markers. Error bars = SD, *n* = 3 biological replicates. TPM, transcripts per million. Statistical significance was assessed by one-way ANOVA; only *p*-values < 0.05 are shown. Source data are provided as a Source Data file.

To identify cellular vulnerabilities heightened by K27M mutations, we employed isogenic K27M-mutant and K27M-KO gliomasphere cultures of the patient-derived DMG cell line DIPG-XIII^34,44^. DIPG-XIII has been used extensively in a large number of DMG studies^24,26,34,36,41,44,45^, and harbours a single copy of the H3.3K27M mutant allele in *H3F3A*, as well as mutations in *TP53* and amplification of wild-type *HIST1H3B*^45^. We conducted genome-wide CRISPR dropout screens in K27M and K27M-KO lines cultured in tumor stem medium (TSM) (Fig. 1a and Supplementary Fig. 1). As expected, regardless of K27M status, sgRNAs targeting common essential genes were depleted over time (Fig. 1b) while control (non-targeting) sgRNAs were stably maintained (Fig. 1c). The most significant K27M-specific vulnerability, as identified by sgRNA dropout in K27M but not K27M-KO cells, was the lineage-specific transcription factor ASCL1 (Fig. 1d, e and Supplementary Data 1). This aligns with previous findings that H3K27M directly impacts ASCL1 expression and sensitivity to transient ASCL1 knockdown^44,46^.

ASCL1, a bHLH transcription factor, is a well-established regulator of neuronal differentiation and reprogramming during development^47,48^. Sustained *ASCL1* expression is critical for neuronal reprogramming during oligodendrogliogenesis, functioning as an “on-target” pioneer factor that opens repressed chromatin and activates neuronal genes, often in coordination with co-factors^49^. ASCL1 activity is regulated by phosphorylation^50^ and cell cycle stage^51^, and marks distinct tumor subpopulations in lung^52^, prostate^53^, and CNS cancers^9,31,54^. In glioblastoma models, ASCL1 exhibits dual roles as both a tumor promoter and suppressor^55–57^. In oncohistone-mutant gliomas, we and others have reported high *ASCL1* expression in interneuron progenitor-derived H3.3G34R gliomas^25,44,58,59^.

We assessed *ASCL1* expression in both published and in-house high-grade glioma (HGG) transcriptome datasets^29,36,42,58,60^. High *ASCL1* expression was noted across all HGGs, particularly in K27M-mutant DMGs, compared to lower-grade gliomas and ependymomas (Supplementary Fig. 2a). As predicted, *ASCL1* loss resulted in a mild but statistically significant reduction in proliferation specifically in K27M-mutant cells (Fig. 1f, g). Remarkably, in an in vivo patient-derived orthotopic xenograft (PDOX) model, where control and ASCL1-KO CRISPR-edited DIPG-XIII cells were xenografted into the pons of 4–6-week-old NSG mice, none of the mice injected with ASCL1-KO DIPG-XIII cells developed tumors over a one-year follow-up (Fig. 1h, i). This lack of tumor formation occurred despite the presence of K27M and other oncogenic mutations such as TP53 mutation and MYCN amplification, similar to our observations following K27M removal^34,36,61^. Consistently, K27M-mutant tumor cells were virtually absent in ASCL1-KO xenograft sections (Supplementary Fig. 2b). In line with these results, high *ASCL1* expression in human DMGs was significantly associated with reduced patient overall survival (p = 0.018; Supplementary Fig. 2c). These data support an oncogenic role for *ASCL1* in DMGs.

### ASCL1 maintains RGC/OPC stem states in DMG

To explore how *ASCL1* drives oncogenicity in K27M-DMG, we began by examining its expression in the developing brain. Single-cell transcriptomic atlases of the human first trimester brain revealed that *ASCL1* is highly expressed during fetal brain development, including in glial progenitors, radial glial cells (RGCs), and neuroblasts (Supplementary Fig. 3a). *ASCL1* expression was enriched in neuronal and glial progenitors as well as OPCs within the developing brain in both human and mouse and restricted to OPCs in the adult pons (Supplementary Fig. 3b–d), aligning with the role of this TF in neuroglial differentiation^62,63^. It was depleted across the lifespan in astrocyte/ependymal-like cells. These data indicate that *ASCL1* expression is highly elevated in glial progenitors of the developing brain midline predisposed to oligodendrogenesis, which have been shown to be targeted by K27M mutations^31,36,42^. Accordingly, when we investigated single-cell transcriptomes of K27M-mutant tumors, this expression pattern was mirrored in tumors where *ASCL1* was also mainly enriched in cycling progenitors, RGCs and OPCs (Supplementary Fig. 4).

We next performed transcriptomic analyses of isogenic DMG cell lines to assess differentially expressed genes following ASCL1 removal. Notably, ASCL1 loss in a K27M-mutant background dysregulated nearly three times more genes than in a K27M-KO background (Supplementary Fig. 5a, b), indicating that ASCL1 may adopt unique gene regulatory roles in the presence of K27M. To identify the glial lineages impacted by ASCL1 loss, we performed gene set enrichment analysis (GSEA) using cell type-specific signatures derived from single-cell RNA sequencing (scRNA-seq) atlases of the human and mouse hindbrain and thalamus during development^36,64,65^. ASCL1-KO significantly reduced proliferative gene signatures, including the expression of cell cycle genes such as *AURKB*, *MCM7*, and *CDCA5*, which resembles the effects of K27M removal (Fig. 1j, k). Interestingly, while K27M loss promoted oligodendroglial differentiation signatures (e.g., *CLDN11*, *TUBB4A*), transcriptomes from ASCL1-KO glioma lines uniquely showed strong enrichment of astro-mesenchymal and ependymal signatures (e.g., *GJA1*, *S100A10*), indicating preferential skewing to these lineages in the absence of ASCL1 (Fig. 1j, l). Although ASCL1 and K27M converge in their impact in reducing proliferation, these findings highlight their non-overlapping roles in maintaining lineage specificity and oncogenic potential.

### ASCL1 and K27M cooperate to block differentiation and plasticity in DMG

**Fig. 2:**
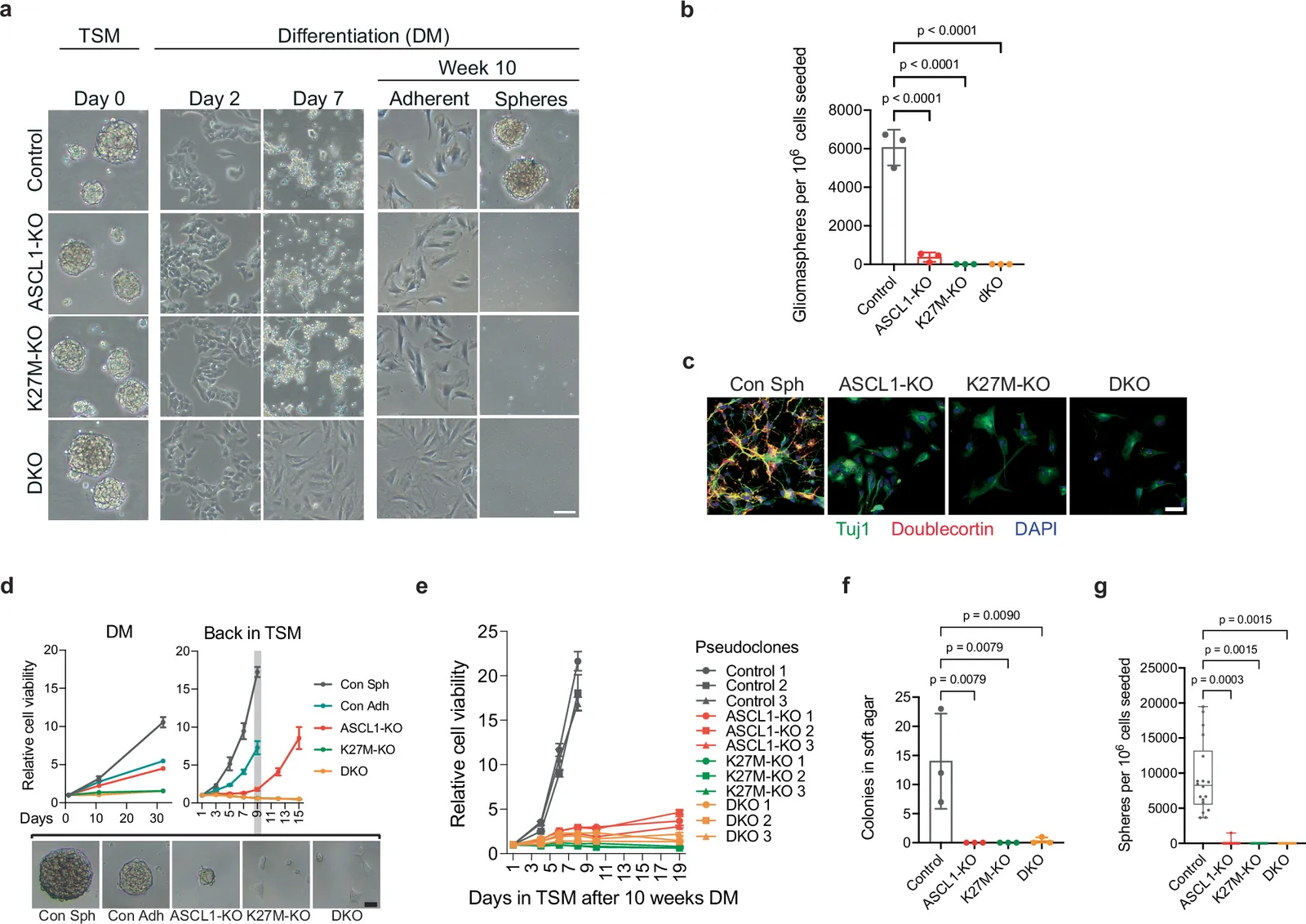
ASCL1 and K27M promote phenotypic plasticity. **a** Representative light micrographs of DIPG-XIII Control and KO cells undergoing serum-induced differentiation. On Day 0, cells cultured in TSM were dissociated into single cell suspensions and seeded in DM. Bar = 50 µm. Data are representative of five independent experiments. **b** Quantification of DIPG-XIII spheres in suspension after 5 weeks in DM. Data are shown as mean ± SD, *n* = 3 biological replicates. Statistic: one-way ANOVA. **c** Immunofluorescence images of DIPG-XIII cells stained for neuronal differentiation markers Tuj1 (green) and Doublecortin (red). After 2 months of culture in DM, Con Sph and KO adherent cells were treated with neural differentiation medium for 2 weeks prior to immunostaining. Bar = 50 µm. Data are representative of two independent experiments. **d** Above: CellTiterGlo assays for relative cell viabilities of the indicated genotypes, in DM and TSM. Cells were cultured in DM for 2 months prior to the start of the experiment (Day 0), and were either continued in DM for another 30 days or returned to TSM. Error bars represent SD, *n* = 4 wells. Below: representative light micrographs of indicated genotypes 10 days post-return to TSM. Bar = 10 µm. Data are representative of two independent experiments. **e** CellTiterGlo assays for relative cell viability, from expanded DIPG-XIII pseudoclones (*n* = 3 independent pseudoclones per condition) cultured in DM for 4 weeks prior to the start of the experiment (Day 0). Data are shown as mean ± SD, *n* = 4 technical replicates per clone. **f** Quantification of DIPG-XIII colonies in soft agar after 10 weeks in DM. 2250 cells were seeded per replicate. Average % of sphere-forming control cells is indicated. Data are shown as mean ± SD, *n* = 3 biological replicates. Statistic: one-way ANOVA. **g** Quantification of DIPG-XIII gliomaspheres after 4 weeks in DM. Each dot represents an individually expanded pseudoclone (starting from 1 to 3 cells) of the indicated genotype prior to DM exposure (*n* = 18 pseudoclones for Control, *n* = 12 pseudoclones for ASCL1-KO, *n* = 9 pseudoclones for K27M-KO, *n* = 9 pseudoclones for DKO). Bounds of box indicate 25th and 75th percentiles. Center line indicates median; whiskers indicate min to max. Statistic: one-way ANOVA. Source data are provided as a Source Data file.

Upregulation of distinct differentiation markers in K27M-KO and ASCL1-KO cells in TSM led us to hypothesize that inducing differentiation in DMG cell lines might further reveal the role of ASCL1 and its functional interactions with K27M. We first examined gliomasphere-forming capacity under prolonged serum-containing differentiation medium (DM) treatment (Fig. 2a). In TSM, gliomaspheres were present in all control, K27M-KO, ASCL1-KO, and double-KO (DKO) conditions. Gliomaspheres were rapidly lost upon DM exposure, and adherent, fibroblast-like cell morphologies emerged in all groups. This transition was fastest in the DKO cells, suggesting a synergistic effect of ASCL1 and K27M loss in promoting differentiation (Fig. 2a). Interestingly, in contrast to short-term differentiation, prolonged DM culture revealed unique phenotypic plasticity in control cells. After 2–3 weeks of increased cell death, control cells exhibited a striking re-emergence of gliomaspheres (DM spheres) alongside adherent fibroblast-like cells (Fig. 2a). These DM spheres were absent in ASCL1-KO, K27M-KO, and DKO cells, indicating that loss of either factor prevents their formation and promotes further differentiation (Fig. 2a, b).

Two additional lines of evidence support the dependence of DMG plasticity on both ASCL1 and K27M. First, after 10 weeks in DM, only the DM spheres retained the ability to differentiate into neural cells, suggesting that a functional OPC/RGC-like state persists only when both ASCL1 and K27M are present (Fig. 2c). Second, among the adherent cells at week 10 in DM, the control adherent subpopulation was the most reversible. Upon returning to TSM, these adherent cells re-formed gliomaspheres and resumed rapid proliferation (Fig. 2d). In contrast, this reversibility was delayed in ASCL1-KO cells and abolished in K27M-KO and DKO cells (Fig. 2d). Testing reversibility in pseudoclonal populations (derived from 1 to 3 cells each) showed that none of the DM-differentiated pseudoclones from ASCL1-KO, K27M-KO, or DKO lines regained rapid proliferation upon returning to TSM (Fig. 2e). Therefore, the single loss of ASCL1 or K27M is sufficient to reduce phenotypic plasticity under prolonged differentiation conditions.

To estimate the fraction of control cells capable of forming spheres in DM, we performed soft agar assays and found ~0.6% (42/6750) of control cells formed colonies after 10 weeks in DM (Fig. 2f). To rule out the possibility of genetic heterogeneity influencing differentiation and sphere-forming ability in DM, we tested pseudoclonal populations, each derived from 1 to 3 cells. If sphere formation in DM were a rare, genetically predetermined ability, we would expect only a small fraction of pseudoclones to form spheres. However, all (18/18) control pseudoclones formed gliomaspheres after 4 weeks in DM (Fig. 2g), indicating that resistance to differentiation is not genetically predetermined but is a plastic characteristic. When DM spheres were returned to TSM, they maintained a memory of their sphere-forming ability upon subsequent DM exposure. This memory gradually waned over 10 weeks (Supplementary Fig. 6a, b). These findings underscore the critical role of ASCL1 and K27M in promoting plasticity and driving phenotypic heterogeneity in DMGs. More broadly, our results suggest that prolonged differentiation could serve as a valuable method for identifying critical plasticity regulators that otherwise display mild phenotypic effects in stem cell conditions or short differentiation timepoints. Notably, these observations are reminiscent of drug tolerant persisters^66^, and suggest that chromatin-regulated states of resistance to differentiation can eventually be reversed upon removal of differentiation cues.

### ASCL1 and K27M cooperate to maintain gliomaspheres and stemness in DM

**Fig. 3:**
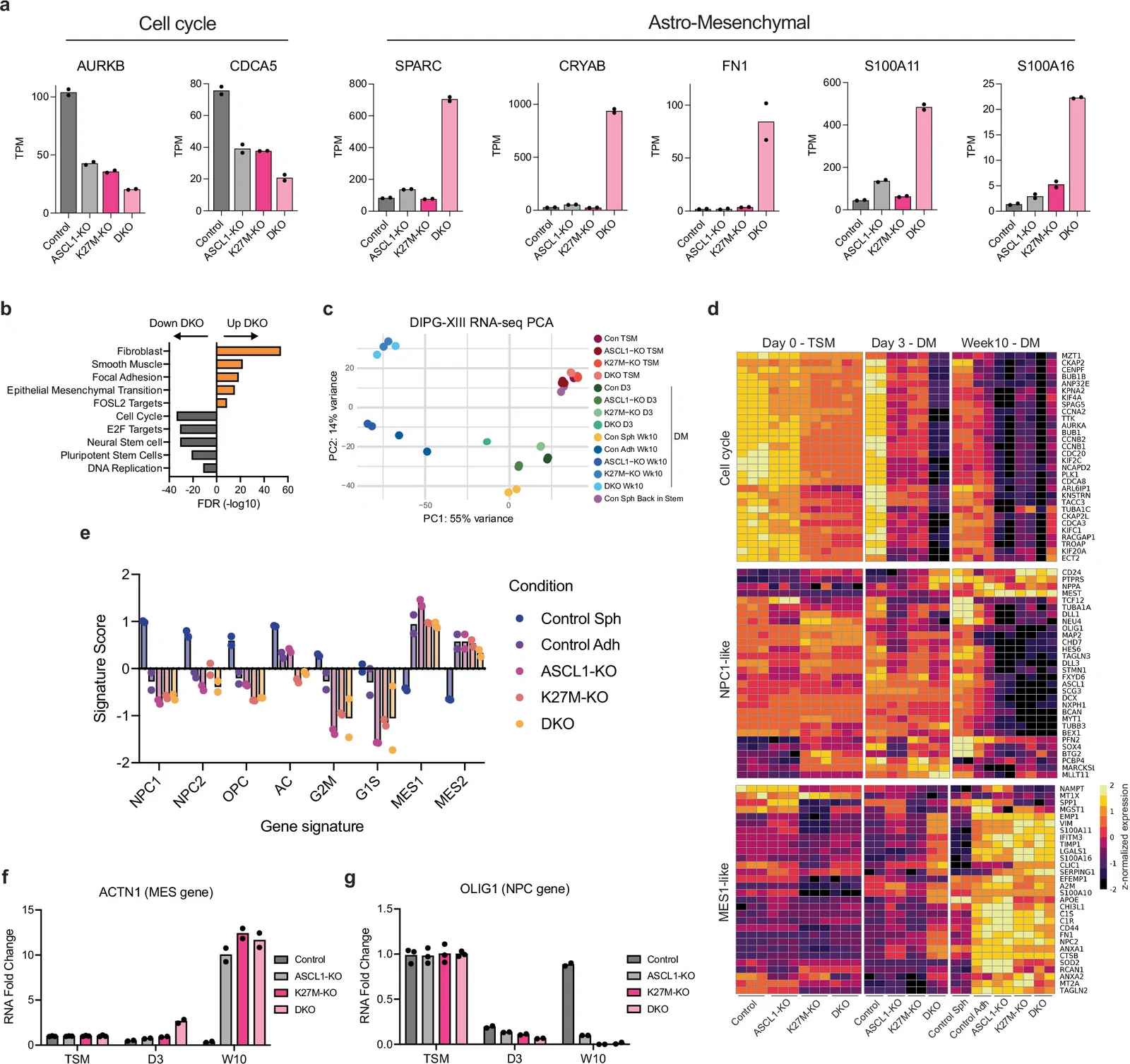
ASCL1 and K27M cooperate to maintain proliferative NPC-like states. **a** TPM (transcripts per million) of cell cycle and astro-mesenchymal genes at Day3 post-differentiation (*n* = 2 independent replicates). **b** Select gene sets from the Enrichr database, enriched among the cooperatively down- and upregulated genes, in DIPG-XIII D3 DKO vs. D3 Control. x-axis represents negative log of adjusted *p*-value of enrichment. **c** PCA plot of DIPG-XIII RNA-seq throughout differentiation. “Con Sph Back in Stem” refers to Control gliomaspheres being returned to TSM for 2 weeks, after 10 weeks in DM. **d** Heat maps of z-score normalized expression of Cell Cycle (G2/M), NPC1, and MES1 gene signatures derived from adult and pediatric glioblastomas (Neftel et al., 2019). **e** Relative expression of all metamodules defined in Neftel et al., 2019 in Wk10 DM samples (*n* = 2 independent replicates). **f**, **g** Relative expression of ACTN1 (a MES gene) and OLIG1 (an NPC gene) across differentiation conditions. At Week 10, “Control” refers to the DM sphere subpopulation. TSM Tumor Stem Medium, D3 Day 3 in Differentiation Medium, W10 Week 10 in Differentiation Medium. **a**–**g** RNA-seq was performed as *n* = 3 biological replicates in TSM and *n* = 2 biological replicates in DM. Source data are provided as a Source Data file.

To investigate the molecular mechanisms driving gliomasphere emergence in DM and their dependence on ASCL1 and K27M, we performed RNA sequencing (RNA-seq) on cells cultured in TSM and DM. Initially, we focused on early differentiation responses. After 3 days of serum-containing DM treatment, DKOs exacerbated the downregulation of stemness and cell cycle genes (e.g., *AURKB*, *CDCA5*) compared to single KOs, and synergistically upregulated astro-mesenchymal markers (e.g., *SPARC*, *CRYAB*, *FN1*, *S100A11*, *S100A16*) (Fig. 3a), highlighting the cooperative role of ASCL1 and K27M in maintaining DMG stemness and repressing mesenchymal differentiation (Fig. 3b). In total, 382 and 598 genes were cooperatively down- and upregulated in DKOs, respectively (Supplementary Data 2). These genes were not significantly altered in TSM (Supplementary Fig. 7a), highlighting the importance of differentiation conditions to uncover oncohistone-TF cooperativity in gene regulation.

When cells grown for 10 weeks in DM were included in the analysis, principal component analysis (PCA) revealed a distinct distribution pattern based on genetic manipulation and culture conditions (Fig. 3c). The transcriptome of gliomaspheres from 10-week DM cultures resembled that of cells grown in TSM. In contrast, the transcriptome of ASCL1-KO, K27M-KO, and DKO cells largely showed increased expression of mesenchymal genes (Fig. 3d). These findings were further validated by expression modules derived from primary glioma patient samples^9^ (Fig. 3e). The DM spheres resisted differentiation-induced expression changes (e.g., *ACTN1*) observed in all KO lines (Fig. 3f and Supplementary Fig. 7b) and maintained OPC/immature neural programs enriched for ASCL1 targets (e.g., *OLIG1*) (Fig. 3g and Supplementary Fig. 7c). Re-introduction of *ASCL1* into ASCL1-KO cells partially rescued sphere re-emergence in DM (Supplementary Fig. 8a–c), re-activated NPC gene expression, and re-silenced MES gene expression (Supplementary Fig. 8d). Thus, ASCL1 is necessary and sufficient to promote NPC programs in DMG. Together our data suggest ASCL1 and K27M drive a cycling progenitor state despite differentiating conditions.

### K27M prevents PRC2-mediated ASCL1 silencing in differentiating RGCs/OPCs

**Fig. 4:**
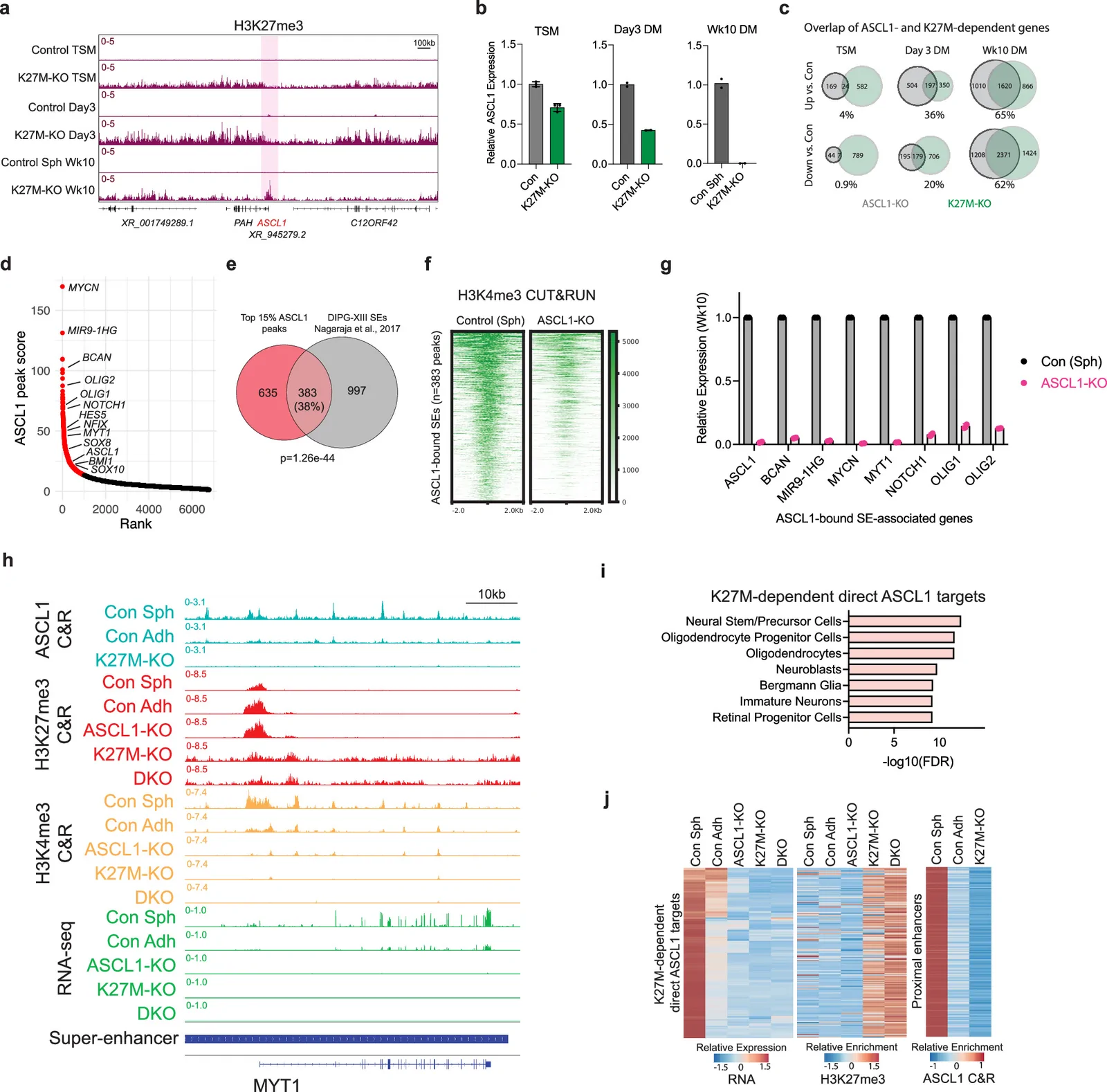
ASCL1 is a key mediator of K27M-dependent differentiation resistance. **a** IGV tracks of H3K27me3 enrichment in Control and K27M-KO cells. TSM H3K27me3 data are averages of two independent CUT&Tag reactions. Day3 and Wk10 H3K27me3 data in DM are averages of one CUT&RUN and two CUT&Tag reactions (total *n* = 3 per condition). **b** Relative *ASCL1* expression from RNA-seq in indicated conditions. Data are shown as mean of *n* = 3 independent experiments in TSM and *n* = 2 independent experiments in DM. **c** Venn diagrams indicating overlap of significantly differentially regulated genes (abs(Log2FoldChange > 1) and adjusted *p*-value < 0.05) between ASCL1-KO vs. Control (gray circles) and K27M-KO vs. Control (green circles), at indicated time points. *p*-values from Wald tests were adjusted for multiple comparisons using the Benjamini–Hochberg method. The % of genes dysregulated in K27M-KO which are also dysregulated in ASCL1-KO is indicated. RNA-seq was performed as *n* = 3 biological replicates in TSM and *n* = 2 biological replicates in DM. **d** ASCL1 CUT&RUN peaks in Wk10 DM Control gliomaspheres ranked by MACS2 peak score. The top-scoring 15% of peaks are shown in red. Genes nearest select top peaks are indicated. **e** Overlap between the top 15% (*n* = 1018) ASCL1 CUT&RUN peaks in Wk10 DM Control gliomaspheres with previously reported DIPG-XIII superenhancers^67^. *P*-value of a two-sided hypergeometric test is shown. **f** Heat maps of H3K4me3 CUT&RUN signal at the shared regions in (**e**). **g** Relative expression of SE-associated direct ASCL1 targets highlighted in (**d**), *n* = 2 independent biological replicates. **h** IGV tracks showing CUT&RUN (C&R) for ASCL1, H3K27me3, and H3K4me3, and RNA-seq signal, at an NPC gene (MYT1) locus. Super-enhancer annotations were lifted from Nagaraja et al., 2017. **i** Gene set enrichment (Enrichr) of ASCL1- and H3K27M-dependent genes associated with the top 15% ASCL1 in Wk10 DM Control spheres (*n* = 184 genes). x-axis represents negative log of adjusted *p*-value of enrichment. **j** Heatmaps of relative RNA expression, H3K27me3 enrichment across the gene body, and ASCL1 enrichment at the nearest ASCL1 peak, of the 184 ASCL1- and H3K27M-dependent genes in Wk10 DM Control spheres. Color scales represent z-normalized expression (for RNA) and enrichment (for ASCL1 and H3K27me3). Source data are provided as a Source Data file.

Profiling H3K27me3 using CUT&RUN and CUT&Tag techniques revealed the mechanistic basis for the similar phenotypes observed between ASCL1-KO and K27M-KO cells. In K27M-KO cells, differentiation led to a strong, specific accumulation of Polycomb-mediated H3K27me3 at the *ASCL1* locus (Fig. 4a), which resulted in a complete loss of *ASCL1* expression by Week 10 in DM (Fig. 4b). Notably, ASCL1-KO increasingly phenocopied K27M-KO in DM. By Week 10, ASCL1-KO cells recapitulated 65% of the upregulated genes and 62% of the downregulated genes seen in K27M-KO cells (Fig. 4c and Supplementary Fig. 9a). These findings strongly suggest that ASCL1 acts as a key effector of K27M-dependent plasticity, with its expression being enabled by K27M’s inhibition of Polycomb-mediated silencing, allowing resistance to further lineage differentiation.

### ASCL1 and K27M cooperatively activate (super)enhancer-linked NPC/OPC Genes

To further explore how ASCL1 maintains a stem-like, neural progenitor state, we performed CUT&RUN for ASCL1 in DM spheres. We identified that many of the genes associated with the top 15% of ASCL1 peaks are known drivers or markers of glioma stemness (Fig. 4d). Notably, 38% of these top peaks overlapped with previously reported super-enhancers (SEs) in DIPG-XIII gliomaspheres from TSM cultures^67^ (Fig. 4e). At these SEs, ASCL1 was directly required for H3K4me3 – a mark associated with active transcription (Fig. 4f), as well as the expression of SE-associated genes (Fig. 4g, h). 23% (184/802) of the top ASCL1-bound genes, which were significantly enriched in RGC/OPC markers (Fig. 4i), depended on both ASCL1 and K27M for expression, with K27M playing a crucial role in preventing H3K27me3 accumulation at most of these genes (Fig. 4j). These results reveal an important cooperative relationship between ASCL1 and K27M: the combination of K27M-mediated inhibition of PRC2 silencing and ASCL1-driven transcriptional activation is essential for maintaining high expression of SE-regulated neural progenitor genes. This cooperation allows stem-like DM spheres to persist even in differentiating conditions.

### Dual states of ASCL1 and polycomb inhibition underlie phenotypic plasticity in DMG

**Fig. 5:**
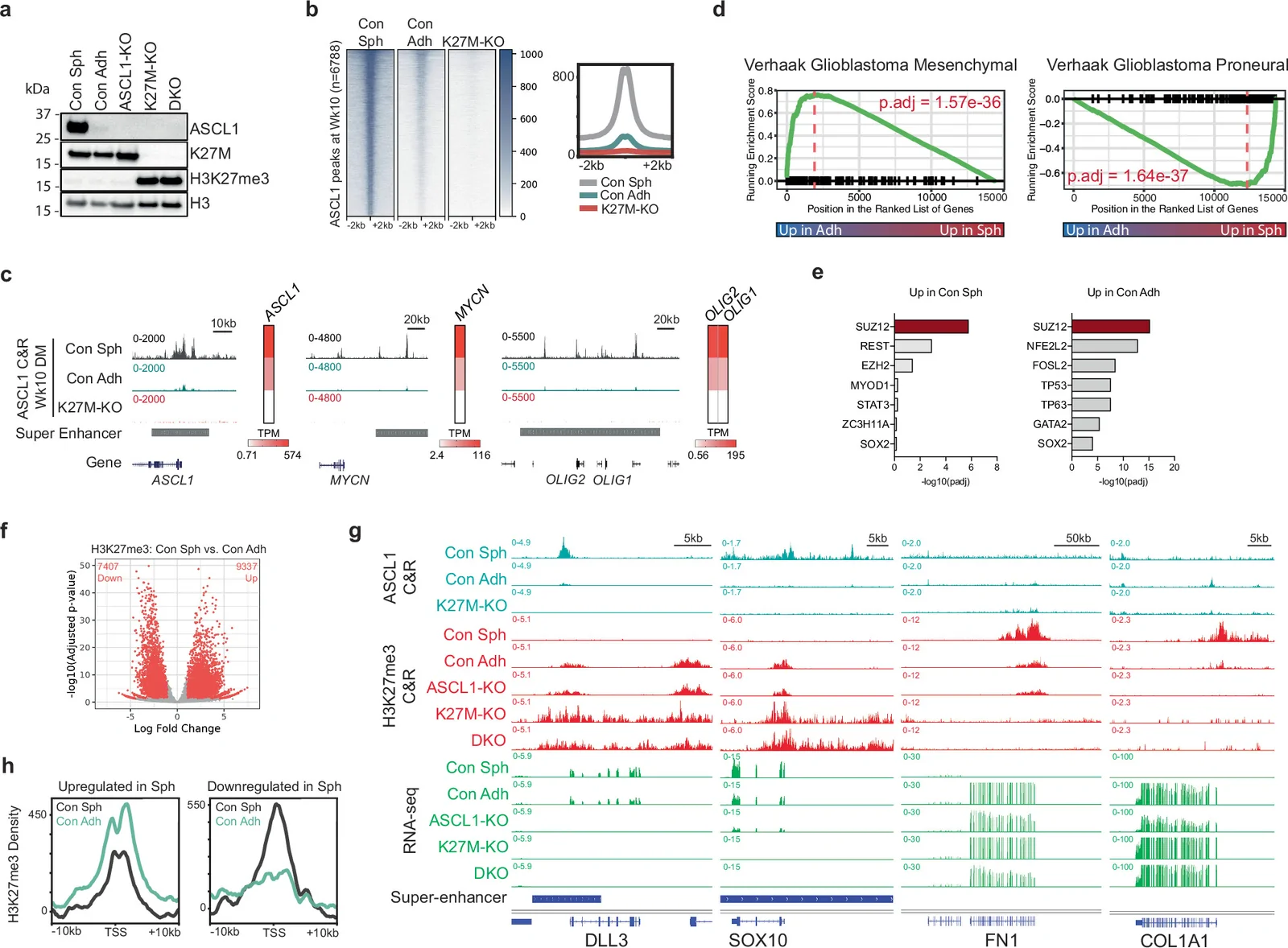
Dual states of ASCL1 and H3K27me3 underlie phenotypic plasticity. **a** Western blots of ASCL1, H3K27M, and H3K27me3, with histone H3 as loading control, of W10 DM samples. Data are representative of *n* = 3 independent experiments. **b** Heat map and density plot of spike-in-normalized ASCL1 CUT&RUN density at ASCL1 peaks called in Con Sph and Con Adh at Wk10 in DM (average of 2 independent replicates). **c** IGV tracks of ASCL1 CUT&RUN enrichment at select super-enhancer-associated NPC/OPC genes^67^, with heat map of mRNA levels (TPM, transcripts per million). **d** Gene set enrichment analysis (GSEA) of differential gene expression between Control Sphere (Sph) and Control Adherent (Adh) subpopulations at Wk10 (*n* = 2 biological replicates). Genes were ranked from most upregulated in Adh to most upregulated in Sph (ranked by Wald statistic), and compared against Verhaak mesenchymal and proneural genes. Enrichment significance was assessed by permutation testing, and *p*-values were adjusted using the Benjamini–Hochberg method. **e** Enrichr analysis of the 150 genes uniquely upregulated (Log2FoldChange > 1.5 & adjusted *p*-value < 0.01) in DM spheres relative to Day0, Day3, and Con Adh (“Up in Con Sph”), and of the 722 genes uniquely upregulated in Con Adh relative to Day0, Day3, and DM spheres (“Up in Con Adh”). *n* = 2 biological replicates. *p*-values from Wald tests were adjusted for multiple comparisons using the Benjamini–Hochberg method. **f** Volcano plot of differential H3K27me3 enrichment between control subpopulations at Wk10 in DM. Significantly differential peaks (Log2FoldChange > 1 & adjusted *p*-value < 0.05) are shown in red. *p*-values from Wald tests were adjusted for multiple comparisons using the Benjamini–Hochberg method; *n* = 3 biological replicates. **g** IGV tracks of ASCL1 and H3K27me3 enrichment and RNA-seq signal at select genes at Week10 in DM. Tracks are averages of *n* = 2 biological replicates for ASCL1, *n* = 3 biological replicates for H3K27me3; *n* = 2 biological replicates for RNA-seq. **h** Profile plots of H3K27me3 density (average of 3 biological replicates) centered around transcriptional start sites (TSSs) of up- and downregulated genes between control subpopulations. Source data are provided as a Source Data file.

Next, we examined differences between control subpopulations (spheres vs. adherent cells) in DM to further understand the mechanisms behind phenotypic plasticity. We observed that ASCL1 protein levels were nearly undetectable in adherent control cells after 10 weeks in DM, as measured by Western blot (Fig. 5a) and CUT&RUN (Fig. 5b). Reduced ASCL1 binding at SEs correlated with lower expression of RGC/OPC genes, including ASCL1 itself, MYCN, and OLIG1/2 (Fig. 5c). Phenotypic variation along a clinically relevant proneural-mesenchymal axis between spheres and adherent control cells in 10-week DM was confirmed by GSEA (Fig. 5d). These findings demonstrate that genetic ablation of ASCL1 is sufficient to deplete RGC/OPC-like states and promote MES-like states, even in the presence of K27M. However, ASCL1 can also be silenced in a subpopulation of cells without genetic manipulation, allowing MES-like differentiation. Remarkably, 96% of upregulated genes in control adherent cells were also upregulated in KO lines (Supplementary Fig. 9b, c), indicating that downregulation of ASCL1 mirrors K27M removal and strongly supporting ASCL1 as a master regulator of DMG cell states. We conclude that K27M promotes high ASCL1 expression to sustain RGC/OPC-like states in DM, but under differentiation pressure, a subset of K27M cells can still silence ASCL1, leading to an ASCL1-low, MES-like state, thereby enabling phenotypic plasticity.

Interestingly, the core PRC2 subunit SUZ12 emerged as the most enriched transcriptional regulator for both upregulated and downregulated genes in DM spheres compared to control adherent cells (Fig. 5e). In addition to the dual states of ASCL1, we observed significant plasticity in H3K27me3 deposition between spheres and control adherent cells grown in DM. Specifically, 16,744 H3K27me3 peaks were differentially enriched between DM spheres and control adherent cells (Fig. 5f), despite unchanged global levels of K27M and H3K27me3 (Fig. 5a). Notably, PRC2 preferentially targeted RGC/OPC genes in adherent cells (e.g., *DLL3*) and the neural crest marker *SOX10*^68^, while in DM spheres, it preferentially targeted MES genes (e.g., *FN1*) and the fibroblast marker *COL1A1* (Fig. 5g). Consistent with PRC2-mediated gene silencing, genes upregulated in DM spheres showed a decrease in H3K27me3 at their promoters, while downregulated genes exhibited an increase in H3K27me3 levels (Fig. 5h). These findings indicate that, despite K27M, recruitment of PRC2 to chromatin and its deposition of the H3K27me3 mark can still be modulated in response to selective pressures leading to variable deposition of the mark within different cells of the same population. This epigenetic plasticity promotes phenotypic flexibility, allowing RGC/OPC-like and MES-like subpopulations to coexist, even when the H3K27me3 mark is unable to spread and remains as narrow peaks in both subpopulations.

### ASCL1 and K27M cooperatively direct BRG1 deposition to maintain RGC/OPC-stem states

**Fig. 6:**
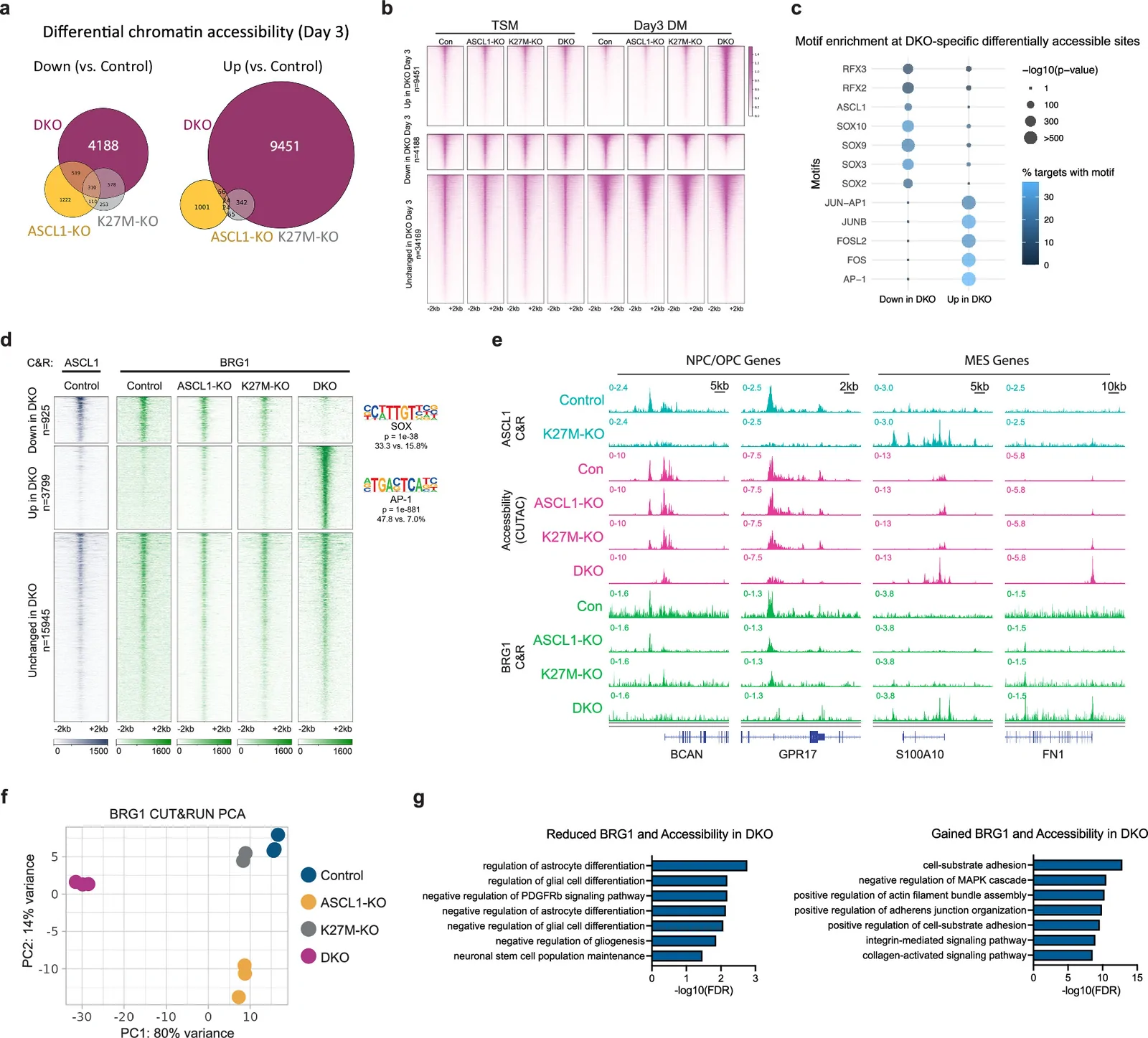
ASCL1 and K27M cooperatively direct BRG1 deposition to maintain RGC/OPC-stem states. **a** Venn diagrams depicting the number and overlap of differentially accessible regions (abs(Log2FoldChange) > 1 and adjusted *p*-value < 0.05) between Control, single-KO, or double-KO (DKO) cells, as measured by CUTAC at Day 3 DM. *p*-values from Wald tests were adjusted for multiple comparisons using the Benjamini–Hochberg method (*n* = 2 biological replicates per condition). **b** Heat maps of CUTAC (chromatin accessibility) signal averages (*n* = 2 biological replicates per condition) at the uniquely downregulated (*n* = 4188) and upregulated (*n* = 9451) peaks in DKOs, defined as those with abs(Log2FoldChange) > 1 & *p*adj <0.05 in DKO vs. Control, and not single KOs vs. Control, in DIPG-XIII at Day3 in DM. Unchanged peaks (*n* = 34,169) are also shown. *p*-values from Wald tests were adjusted for multiple comparisons using the Benjamini–Hochberg method. **c** Enrichment of known sequence motifs identified with HOMER, at uniquely up/downregulated CUTAC peaks in Day3 DKO vs. Control (*n* = 2 biological replicates). Dot size represents negative log of nominal *p*-values (hypergeometric test), colors represent % of sequences containing the indicated motif. **d** Heat maps of spike-in-normalized average CUT&RUN signal density for ASCL1 (*n* = 2) and BRG1 (*n* = 3) in DIPG-XIII at Day 3 DM. The set of 925 peaks represents the triple intersection of regions bound by ASCL1, with DKO-specific reduced accessibility, and reduced BRG1 occupancy. The set of 3799 peaks represents the intersection of DKO-specific increased accessibility with increased BRG1 occupancy. Unchanged peaks (*n* = 15,945) are also shown. The most enriched known motif identified by Homer is indicated for differentially regulated peaks. Percentages indicate % of target sequences containing the motif vs. a GC-matched background set of genomic regions. **e** IGV tracks showing ASCL1, accessibility, and BRG1 signal at NPC/OPC and MES genes. **f** PCA plot of BRG1 CUT&RUN at Day3 in DM. The top 500 most variable peaks were used in the analysis. **g** GREAT analysis of the two peak sets (*n* = 925 and 3799) shown in (**d**) with differential BRG1 and accessibility in DIPG-XIII Day3 DKO vs. Control. x-axis represents negative log of Benjamini–Hochberg-adjusted *p*-values (binomial test).

As ASCL1 and/or K27M removal led to significant transcriptomic changes in K27M DMGs, we explored the chromatin landscape and transcription factor (TF) regulation using CUTAC^69^ to assess chromatin accessibility. The CUTAC analysis showed DM-specific synergistic effects on chromatin accessibility, particularly in DKO cells, with 13,639 differentially accessible regions (DARs) unique to DKO cells in DM (Fig. 6a, b). DARs with reduced accessibility in DKOs were predominantly enriched for motifs of neurodevelopmental TF families such as SOX, ASCL1, and RFX, while DARs with increased accessibility were primarily enriched for JUN/AP-1 family motifs (Fig. 6c). These results add to previous findings that AP-1 is a major hub for regulating gene expression in H3.3 WT DMGs^46^, by demonstrating a causal role for ASCL1 and K27M in negatively regulating AP-1 motif accessibility throughout the genome. Over 85% of DARs were located in distal regulatory elements (Supplementary Fig. 10a), suggesting that ASCL1 and K27M cooperatively regulate enhancer accessibility in response to differentiation signals.

To investigate the mechanism behind the cooperative regulation of enhancers, we first performed CUT&RUN for ASCL1 and compared its binding sites with differentially accessible regions (DARs). ASCL1 directly bound to 45% (1865/4188) of the DARs that were closed in DKO cells, but only 4% (421/9451) of the DARs that were opened in DKO cells (Supplementary Fig. 10b). The subset of ASCL1 peaks that lost accessibility in DKO cells was uniquely characterized by co-enrichment of both ASCL1 and SOX motifs (Supplementary Fig. 10c). These results are consistent with ASCL1 directly opening and indirectly closing chromatin, possibly in collaboration with SOX family TFs. SOX TFs have been shown to indirectly repress chromatin accessibility by competing for chromatin remodelers^70^. Moreover, the chromatin remodeler BRG1 (SMARCA4) is aberrantly recruited by SOX10 to chromatin in K27M-mutant DMGs^71,72^.

We therefore investigated BRG1 deposition in our models. BRG1 deposition overlapped with ASCL1 binding at SOX-enriched motifs (Fig. 6d). In ASCL1-KO and K27M-KO lines, this deposition shifted partly to AP1 motif-enriched sites, while in DKO cells after 3 days in DM, it was drastically redistributed there, consistent with the increased chromatin accessibility observed in these conditions (Fig. 6d–f). In all 3 conditions but especially in DKO, BRG1 was redistributed from neuroglial elements to mesenchymal regulatory elements (Fig. 6g), in keeping with the fibroblast-like differentiation observed in culture (Fig. 2a). Genes (*n* = 732) associated with increased BRG1 and chromatin accessibility in DKOs were upregulated at Day 3 and further upregulated after 10 weeks in DM (Supplementary Fig. 10d). Taken together, these data suggest that ASCL1 and K27M cooperate to regulate the BRG1-dependent enhancer landscape in the early response to differentiation. These observations support a model whereby K27M and ASCL1, potentially in cooperation with SOX factors, influence BRG1 redistribution to preserve neuroglial/OPC programs and constrain differentiation. This model is consistent with the previously reported, direct interactions between ASCL1 and BRG1 during neural differentiation^73^, and offers a mechanistic rationale for targeting BRG1 in K27M-mutant DMG suggested by previous studies^71,72^.

### ASCL1 drives phenotypic plasticity and tumorigenesis in a thalamic DMG

**Fig. 7:**
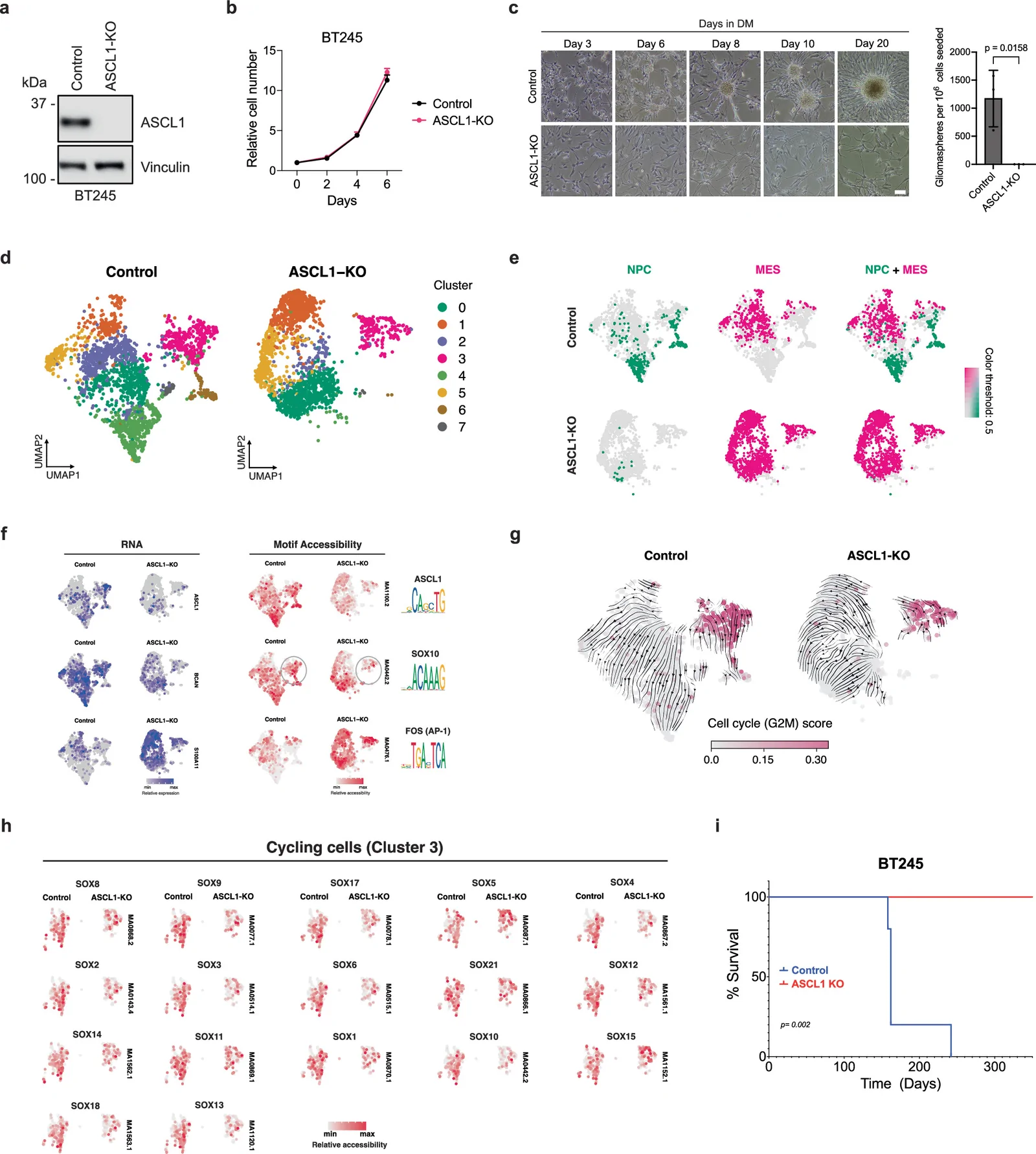
ASCL1 drives plasticity and gliomagenesis in a thalamic DMG model. **a** Western blot confirmation of ASCL1-KO in BT245 cells. Data are representative of three independent experiments. **b** CellTiterGlo assays for relative cell viability of BT245 cells in TSM. Data are shown as mean ± SD, *n* = 4 technical replicates. Data are representative of *n* = 3 independent experiments. **c** Left: Representative light micrographs of differentiating BT245 cells. On Day 0, cells cultured in TSM were dissociated into single cell suspensions and seeded in DM. Bar = 100 µm Right: Quantification of gliomaspheres at Day 20. Data are shown as mean ± SD, *n* = 3 independent biological replicates. Statistic: two-tailed *t*-test. **d**–**g** UMAPs of BT245 (*n* = 2245 Control and *n* = 1806 ASCL1-KO cells) 6 days post-serum treatment, colored by **d** unsupervised cell clusters; **e** expression of NPC-like (NPC1) and MES-like (MES1) gene signatures (Neftel et al., 2019); **f** RNA expression and chromVAR scores of motif accessibility, with gray circles highlighting Cluster 3 cells identified in (**d**); **g** G2/M cell cycle signature score (Neftel et al., 2019) with superimposed RNA velocity vectors. **h** UMAP visualization of Cluster 3 cells identified in (**d**) (*n* = 361 cells in Control and *n* = 180 cells in ASCL1-KO), colored by chromVAR scores of motif accessibility, for all SOX TF motifs in the JASPAR 2020 CORE vertebrate collection. **i** Kaplan–Meier survival curves of orthotopic xenograft mouse models with clonal Control or ASCL1-KO BT245 injected cells into the caudate putamen (*n* = 5 per line; log-rank test). Source data are provided as a Source Data file.

To test whether ASCL1 promotes phenotypic plasticity and tumorigenesis beyond DIPG-XIII, which was derived from the pons, we deleted ASCL1 in a second K27M-mutant DMG cell line, BT245, derived from a thalamic DMG (Fig. 7a). Like DIPG-XIII, removal of K27M in BT245 cells restored H3K27me3 and reduced H3K27Ac (Supplementary Fig. 11). ASCL1-KO did not significantly impair BT245 proliferation in TSM (Fig. 7b). However, as in DIPG-XIII, BT245 gliomaspheres adhered upon serum-induced differentiation, with sphere-like structures re-emerging over time in an ASCL1-dependent manner (Fig. 7c).

To characterize the emerging heterogeneity, we performed single-nucleus multiomics (RNA + ATAC-seq) at Day 6 post-differentiation. Control BT245 cells contained unique transcriptional clusters of cells (clusters 4, 6, 7) largely absent in ASCL1-KO (Fig. 7d), consistent with ASCL1-dependent transcriptional heterogeneity. These additional clusters reflected NPC-like states and, as in DIPG-XIII, ASCL1 loss restricted cells to predominantly MES-like states (Fig. 7e). Consistently, ASCL1 expression and activity was heterogeneous in control cells, and ASCL1-KO downregulated NPC genes (e.g., *BCAN*) while increasing astro-mesenchymal genes (e.g., *S100A11*) and AP-1 motif accessibility (Fig. 7f). These data support a model where plasticity in ASCL1 expression drives transcriptional and phenotypic heterogeneity in multiple K27M-mutant DMG lines, with ASCL1-low cells primed for MES-like differentiation.

Further in line with our findings in DIPG-XIII, ASCL1 loss reduced SOX10 motif accessibility, in certain cell subpopulations (Fig. 7f, gray circles). This was most notable in “Cluster 3”, which corresponded to the actively cycling subpopulation as determined by high cell cycle gene expression (Fig. 7g). RNA velocity vectors projected from Cluster 3 cells toward other cell states (Fig. 7g), suggesting that actively cycling cells in DM were also the most progenitor-like – recapitulating the hierarchies reported in both pediatric and adult gliomas from primary tumor samples^31,54^. Moreover, in these proliferative progenitors, ASCL1-dependent SOX motif accessibility was not restricted to SOX10, but applied broadly to most other SOX TFs (Fig. 7h), further supporting cooperativity between ASCL1 and SOX TFs in driving proliferative, progenitor-like states. As in DIPG-XIII, ASCL1-KO abrogated tumor formation and significantly extended xenograft survival in the BT245 model (Fig. 7i).

**Fig. 8:**
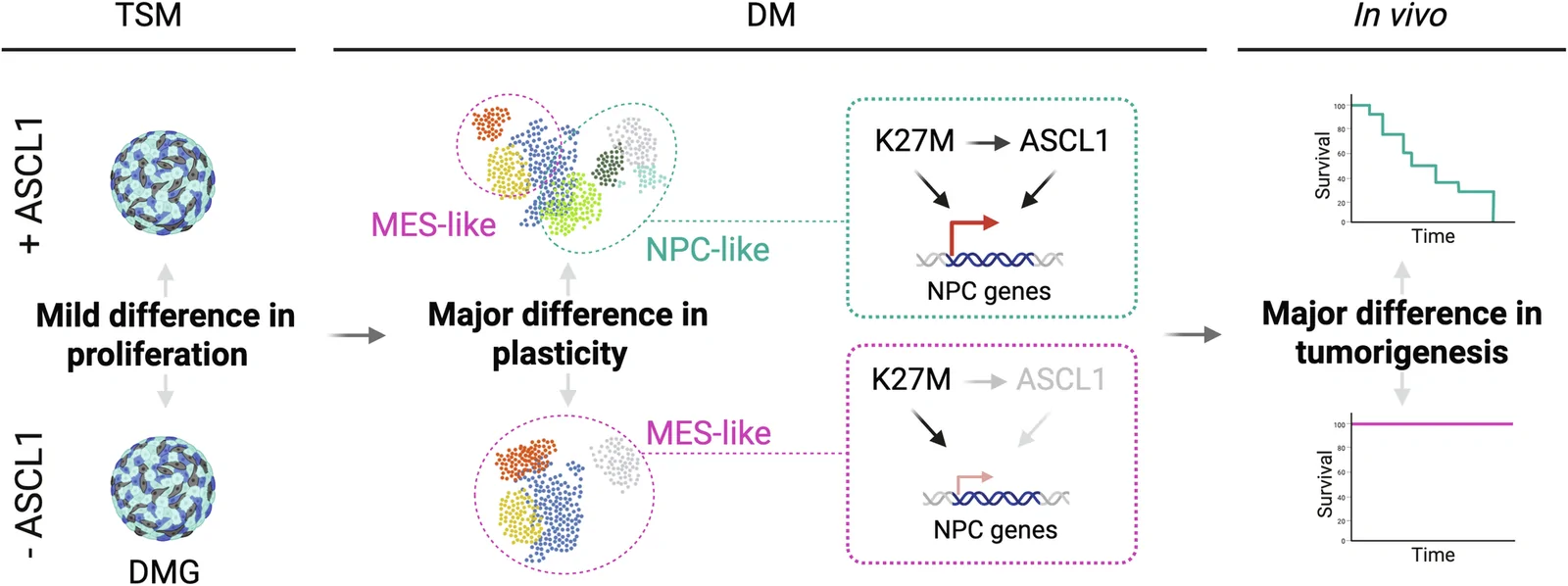
Summary. H3K27M DMGs exhibit ASCL1-dependent neural progenitor states that drive gliomagenesis. This dependency is masked in culture conditions promoting stemness but revealed under differentiating conditions – where ASCL1 expression is dependent on K27M – and in vivo. TSM tumor stem medium, DM differentiation medium, NPC neural progenitor cell, MES mesenchymal. Created in BioRender. Jiao, A. (2026) https://BioRender.com/pbyyye5.

In summary, we identify a critical tumorigenic dependency on ASCL1 in K27M-driven DMGs (Fig. 8). This dependency is masked in culture conditions promoting stemness but revealed under differentiating conditions. Without ASCL1, cells are diverted into non-tumorigenic lineages, blocking gliomagenesis.

## Discussion

We identify ASCL1 as a key downstream effector of K27M in DMG, where it cooperates with this oncohistone to block glial differentiation. Its removal in K27M-DMG significantly impacts oncogenicity, impairing in vivo tumor formation in PDOX models and promoting differentiation along an astrocytic-ependymal path in K27M-mutant cell lines. Long-term culture in DM shows that ASCL1 and K27M are required to maintain a stem-like, proliferative pool of RGC/OPC-like cells that resist differentiation and drive gliomagenesis. Removal of either factor leads to terminal differentiation, severely impairing tumorigenesis. Notably, we show that ASCL1 expression is epigenetically regulated, including by PRC2. ASCL1 expression decreases in cells with mesenchymal-like features that can escape K27M-mediated differentiation blockade, contributing to the phenotypic plasticity observed in K27M-DMG. We also reveal an unexpected adaptability in PRC2 recruitment under selective pressure, as the complex can be removed from certain sites and recruited to others, including *ASCL1*, to silence self-renewal and stem markers. This plasticity is driven by the recruitment of BRG1 to neuroglial regulatory elements, including SOX genes, which are kept open by this pioneer transcription factor, diverting it from AP-1 mesenchymal sites that promote mesenchymal differentiation. Beyond gliomas, our findings suggest that ASCL1 in midline RGC/OPC cells prevents differentiation into astrocytic-ependymal lineages, maintaining these progenitors along an OPC/oligodendroglial path, and that its modulation may improve the in vitro generation of astrocytes and ependymal glia.

The co-existence of RGC/OPC-like and MES-like cell states has been well documented in primary pediatric and adult gliomas^9,42,74,75^, and K27M-driven cell states and epigenetic landscapes represent potential therapeutic targets^76,77^. While recent advances in brain tumor organoids offer unique opportunities to study cancer cell states^78–82^, we find that simple monocultures under homogenous differentiating conditions are also sufficient to reveal phenotypic plasticity and its regulators. Long-term serum-induced differentiation unambiguously reveals phenotypic plasticity in DMGs: RGC/OPC-like spheres in suspension vs. MES-like adherent cultures. We have observed gradual gliomasphere re-emergence in the months following differentiation for many, albeit not all, pediatric glioma lines tested (not shown), suggesting a general approach for studying regulators of glioma plasticity.

We elucidate a K27M/ASCL1/SOX/BRG1 axis that drives blocked differentiation of an RGC/OPC progenitor along the oligodendroglial pathway. There are three distinct mechanisms through which ASCL1 and H3K27M cooperatively function, which differ depending on differentiation conditions. First, in conditions promoting self-renewal (TSM), ASCL1 actively prevents ependymal and astro-mesenchymal differentiation. ASCL1 depletion triggers these alternate differentiation pathways, providing the K27M-mutant cells unable to further commit to the OPC lineage an alternative route to differentiate akin to a “fire-exit” that promotes exit from stemness.

Second, in the initial response to differentiation, ASCL1 acts synergistically with H3K27M to maintain a neural progenitor state through BRG1 recruitment to proneural enhancers, away from mesenchymal enhancers. Interestingly, MES-like cell states are relatively enriched in (H3 wild-type) adult glioblastomas compared to pediatric K27M DMGs, and this difference has been attributed to age-related changes in the immune microenvironment^9,42^. Our data suggest a parallel, tumor-intrinsic mechanism, mediated by K27M and ASCL1, in the active repression of mesenchymal fates in pediatric DMGs.

Third, in prolonged differentiation, ASCL1 expression becomes fully dependent on K27M and functions as a key downstream effector of K27M-driven RGC/OPC-like states. Critically, ASCL1 is epigenetically silenced in a subset of cells, thereby enabling plasticity and the co-existence of RGC/OPC-like and MES-like states. A major implication of these findings is that ASCL1 offers an alternative therapeutic target to K27M itself. Importantly, we uncover a degree of nimbleness in PRC2 recruitment and deposition of H3K27me3 on chromatin that occurs despite the presence of K27M. Even if deposition of the mark remains peaky, it can change in cells based on selective differentiation and possibly other microenvironmental pressures.

While we have demonstrated potent anti-tumor effects of genetically ablating ASCL1 in pediatric gliomas, ASCL1 is not currently easily druggable. One possibility is to use the recently developed TF-targeting PROTAC degrader technology to selectively deplete ASCL1^83,84^ in tumor cells. Alternatively, targeting upstream regulators of ASCL1, such as the lysine-specific demethylase 1A (LSD1) and Notch signaling^85,86^, or the downstream effector BRG1 using available inhibitors as recently shown^71,72^ may provide therapeutic benefit in K27M-DMG. Finally, we note that in vitro differentiation and culture conditions may not fully recapitulate the cellular heterogeneity present in primary tumors, and ASCL1 may functionally interact with additional factors beyond SOX and BRG1 in vivo.

Beyond DMGs, other lineage-specific TFs may cooperate with epigenetic dysregulation to drive cancer cell plasticity through mechanisms analogous to those described here. Our findings suggest such plasticity phenotypes are likely to be missed in growth conditions optimized for self-renewal, in which large-scale screens for cancer dependencies are typically performed. ASCL1 and K27M are two examples whose depletion results in mild proliferative defects in TSM, but dramatic effects on plasticity when challenged with differentiating conditions in vitro, and on gliomagenesis in vivo. Our results suggest that a new class of tumor dependencies may be uncovered in prolonged, suboptimal self-renewal conditions that place stronger selective pressures on increased plasticity.

## Methods

### Ethics statement

Our research complies with all relevant ethical regulations of the Ludwig Institute for Cancer Research, the University of Oxford, the McGill University Health Center Animal Care Committee, and the Canadian Council on Animal Care.

### Cell culture

Diffuse midline glioma cell culture models (SU-DIPG-XIII and BT245) were grown as gliomaspheres in Tumor Stem Medium (TSM), consisting of a 1:1 mixture of DMEM/F-12 and Neurobasal A (Thermo Fisher), supplemented with: 20 ng/mL PDGF-AA (Peprotech), 20 ng/mL PDGF-BB (Peprotech), 10 ng/mL human EGF (Peprotech), 10 ng/mL human bFGF (Peprotech), 2 µg/ml heparin solution (Stem Cell Technologies), and 1X B-27 minus vitamin A, 1 mM sodium pyruvate (Thermo Fisher), 10 mM HEPES (Thermo Fisher), 1x GlutaMax (Thermo Fisher), 1x Non-Essential Amino Acids (Thermo Fisher), and 1x Antibiotic-Antimycotic (Thermo Fisher). SU-DIPG-XIII cells were established in the lab of Dr. Michelle Monje. BT245 cells were provided by Dr. Keith Ligon. Cell line identity was confirmed by STR fingerprinting. Cells were passaged every 3–4 days by sphere dissociation in TrypLE Express Enzyme (Thermo Fisher). HEK293T cells (ATCC) were cultured in high glucose DMEM (Thermo Fisher) supplemented with 10% FBS (Gibco) and 1% Penicillin-Streptomycin. All cultures were grown at 37 °C and 5% CO_2_, and confirmed to be free of mycoplasma contamination.

### Lentivirus generation and infection

To generate individual lentiviral gRNA constructs, HEK293T cells grown to ~80% confluence in 100 mm plates were transfected with 3 µg of pMD2.G (Addgene 12259), 4 µg of psPAX2 (Addgene 12260), and 5 µg of the gRNA construct (in lentiCRISPR v2 Puro (Addgene 52961) for ASCL1, and pLenti SpBsmbI sgRNA Hygro (Addgene 62205) for H3K27M), using lipofectamine 2000 (Thermo Fisher) according to the manufacturer’s protocol. Viral supernatant was harvested 48 h post-transfection, 0.45 µm-filtered, and concentrated overnight in 50% PEG-8000 at 4 °C. Following centrifugation at 1000 × *g* for 20 min, precipitated viral particles were resuspended in 1 mL of DMEM/F-12 media, and 500 µL was used to infect 1 × 10^6^ glioma cells. Infections were performed with spinning (1000 × *g*) of 1 × 10^6^ dissociated glioma cells per well in 12-well plates, for 1 h at room temperature. Following centrifugation, cells were allowed to recover for 48 h before puromycin (1 µg/mL) or Hygromycin (200 µg/mL) selection for 5–7 days. DIPG-XIII cells stably expressing Cas9 were generated in ref. ^85^. Sequences of control (non-targeting) gRNAs used are: 5’- AAAAAGCTTCCGCCTGATGG −3’ and 5’- CTATTGTCGGCTGGATGGA −3’. The gRNA sequence used to target H3.3K27M is: 5’- GAGGGCGCACTCATGCGAG −3’. The gRNA sequence used to target ASCL1 is: 5’- TTGGGGACGTGCTCCCGAA −3’. For the ASCL1-KO rescue construct, a 3xFLAG-ASCL1 cDNA fragment was cloned downstream of a CMV promoter. 3 silent mutations near the ASCL1 gRNA target site were introduced by site-directed mutagenesis to create a gRNA-resistant 3XFLAG-ASCL1 lentiviral expression construct.

To generate lentivirus for the genome-wide CRISPR screen with the MinLibCas9 library (which targets 18,761 human genes with two gRNAs per gene, Addgene 164896)^87^, six 150 mm plates of ~80% confluent HEK293T cells were each transfected with 9 µg of pMD2.G, 13.5 µg psPAX2, and 18 µg of MinLibCas9 library using lipofectamine 2000 according to the manufacturer’s protocol. Virus was harvested and concentrated as above, and titered by infections of serial dilutions. Infection efficiencies were assessed by %BFP using flow cytometry.

### Genome-wide CRISPR/Cas9 screen

DIPG-XIII and DIPG-XIII(K27M-KO) cells were each screened in duplicate, with 7 × 10^7^ cells infected per replicate per line at a multiplicity of infection (MOI) of 0.3. Following 72 h of recovery post-infection, cells were dissociated and selected in puromycin (1 µg/mL) for 7 days. The “Day 0” genomic DNA sample was collected at the end of puromycin selection, and the cells were cultured for an additional 21 days, seeding at least 2 × 10^7^ cells per replicate for each passage to maintain 500x gRNA coverage. The mid-point genomic DNA collection was performed at Day 11, and the endpoint collection at Day 21. DNA isolation from ~2 × 10^7^ cells per sample was performed with Quick-DNA Midiprep Plus Kit (Zymo Research, D4075). For library preparation, all of the isolated DNA was PCR amplified using Q5 Hot Start High Fidelity 2x Master Mix (NEB M0494L), using 5 µg input gDNA per 100 µL reactions, for 24 cycles. Amplicons were cleaned using 0.9x volumes of Ampure XP beads (BECLA63881). A second round of PCR was performed for 8 cycles, to barcode samples for Illumina sequencing. Amplicons were cleaned using 0.7x volumes of Ampure beads, and sequenced on a Nextseq 2000. Approximately 20 million reads were obtained per sample. Statistical analysis of sequencing results was conducted via the MAGeCK (version 0.5.9.5) package^88,89^. Raw sequencing reads were aligned to gRNA sequences via *mageck count* to generate raw counts for each gRNA. These counts were normalized using the median counts of the 200 non-targeting negative control gRNAs to enable comparison across samples. Gene essentiality estimates (beta scores) were derived for each of the K27M and K27M-KO cell lines via *mageck mle* function. Beta scores were normalized using the median scores for 625 positive control essential genes to enable comparison across cell lines to provide correction for proliferation rate differences between cell lines^89^. Statistically significant cell line-specific essential genes were identified via Wald test. DepMap common essential genes were filtered out.

### Western blotting

Cells were lysed in 2x Laemmli buffer (BioRad #1610737). Samples were sonicated in a Bioruptor for 2.5 min (30 s on, 30 s off) on high, boiled for 10 min, and separated on 4–15% polyacrylamide gels (BioRad #4561086). Transfers were performed using a Trans-blot Turbo system (BioRad) onto 0.2 µm nitrocellulose membranes (BioRad). Membranes were blocked in 5% milk (or 5% BSA for anti-H3K27M blots) in PBST (1x PBS + 0.1% Tween-20). Primary antibodies (1:2000 anti-H3K27me3, Cell Signaling Technology #9733; 1:1000 anti-H3K27M, Abcam ab190631; 1:1000 anti-H3K27me2, Cell Signaling Technology #9728; 1:1000 anti-H3K36me2, Abcam ab9049; 1:10,000 anti-beta-Actin-HRP, Santa Cruz Biotechnology sc-47778 HRP; 1:1000 anti-ASCL1, Santa Cruz Biotechnology sc-374104; 1:1000 anti-histone H4, Cell Signaling Technology #2592; 1:2000 anti-histone H3, Cell Signaling Technology #9715) were rocked overnight at 4 °C, washed 3x in PBST, and incubated in 1:10,000 HRP-conjugated secondary antibody (Cell Signaling #7074 or #7076) for one hour at room temperature. Signal was resolved using Clarity Western ECL Substrate (BioRad #1705061) and imaged on a BioRad Chemidoc. Western blots were performed as biological triplicates unless otherwise noted.

### Serum differentiation

Gliomaspheres in TSM were dissociated on Day 0 and seeded as a single cell suspension into differentiation medium (DM; 10% FBS in high glucose DMEM/F-12 (Thermo Fisher #11320033) with 1x Antibiotic-Antimycotic). 1 million cells were seeded per T25 flask. To quantify gliomaspheres in DM, the number of spheres in 10 random fields of views (under 4x or 10x objectives) were counted, averaged, and scaled by flask size to calculate the total sphere number. Images were taken with a Chromyx HD camera connected to a Nikon Eclipse Ts2 inverted microscope equipped with either a 4x or 10x objective.

### Differentiation reversibility

To test differentiation reversibility, gliomaspheres in TSM were first dissociated, seeded into DM in T25 flasks, and cultured for 10 weeks (generating sphere and adherent subpopulations for controls, and only adherent populations for ASCL1-KO, K27M-KO, and DKO). Each culture (either bulk populations (*n* = 2) or pseudoclones (*n* = 3) expanded from 1 to 3 cells) was then dissociated into single cells, and seeded in 96-well plates in either DM or TSM, considered the “Day0” time point, and relative cell viabilities were subsequently monitored by CellTiterGlo.

### Memory of differentiation resistance

To test a functional memory of differentiation resistance, two independent replicates of DIPG-XIII gliomaspheres were collected after 10 weeks in DM, dissociated into single cells, and expanded in TSM for varying lengths of time (1–10 weeks). Expanded cultures were once again dissociated into single cells, and re-seeded in DM for a second time. Spheres in suspension were counted after 5 days of DM re-exposure, a point at which spheres are not observed in cultures exposed to DM for the first time.

### Neural differentiation and immunofluorescence

Sphere and adherent cells cultured in DM for two months were dissociated into a single cell suspension, and seeded onto ornithine/laminin-coated coverslips in 12-well plates in neural differentiation medium: Neurobasal medium with 2% B-27 Serum Free Supplement and 2 mM GlutaMAX-I. Medium was refreshed every 3 days. After 12 days, cells were fixed in 4% PFA for 15 min at room temperature, and blocked in Immunofluorescence Blocking Buffer (Cell Signaling Technology #12411) for 1 h. Samples were incubated in primary antibodies (1:1000 anti-Doublecortin (Cell Signaling Technology #4604); 1:1000 anti-beta-tubulin III (GeneTex GT1338)) in Antibody Dilution Buffer (1X PBS/3% BSA/0.3% Triton™ X-100) overnight at 4 °C. Alexa Fluor 488 and Alexa Fluor 594-conjugated secondary antibodies (Thermo Fisher) were diluted 1:1000 in PBS + 1.5% BSA and incubated for 1.5 h in the dark at room temperature. Samples were washed 3 times in PBS for 5 min each, stained with DAPI (Thermo Fisher). Images were acquired under an LSM 710 Confocal Microscope (Zeiss) equipped with a 10X objective.

### Immunohistochemistry

Formalin-fixed, paraffin-embedded sections of the xenografts of 5µm-thickness were deparaffinized and rehydrated. The IHC was performed with the Discovery Ultra instrument and reagents from Roche following the manufacturer’s instructions. Briefly, antigen retrieval was performed on unstained sections in EDTA buffer (pH = 8) for 32 min for H3K27M at sub-boiling temperature. Slides were then incubated for 24 min at 37 °C with rabbit anti-histone H3 K27M (1:600, RevMab Biosciences, catalogue no. 31-1175-00-S) followed by secondary antibody incubation with OmniMap anti-Rb HRP (Roche, catalogue no. 760-4311) at room temperature for 20 min. Next, detection was performed using ChromoMap DAB Kit (Roche, catalogue no. 760-159). Slides were then counter-stained with the hematoxylin, dehydrated, cleared and cover-slipped for downstream analysis. Slides were digitally scanned at 40x using an Aperio Turbo 2 T scanner (Leica) for morphometric analysis with Imagescope software.

### Cell viability assays

Cell viabilities were measured with CellTiter-Glo assays (Promega) according to the manufacturer’s protocol. For cell proliferation, 1000 cells were seeded per 96-well in at least triplicates. CellTiterGlo measurements were taken immediately (Day 0) and at regular intervals, with all measurements normalized to Day 0.

### Soft agar assays

Soft agar assays were performed in biological triplicates in 12-well plates, with 500 µL of a 0.6% low-melt agarose base layer made up of a 1:1 ratio of PBS to DMEM/F-12 with 10% FBS. The top layer consisted of 500 µL of a 0.3% low-melt agarose top layer made up of a 1:3 ratio of PBS to DMEM/F-12 with 10% FBS, containing 2250 DIPG-XIII cells from gliomasphere cultures in TSM. Cells were maintained at 37 °C in a humidified 5% CO_2_ atmosphere. Fresh differentiation medium (DMEM/F-12 with 10% FBS) was regularly added to each well to prevent drying, and the number of colonies were counted after 10 weeks.

### LogRank test across quartiles of ASCL1 mRNA expression

All available diffuse glioma and CNS/Brain samples from pedcBioPortal with H3K27M mutations in either H3F3A, HIST1H3C, HIST1H3D, HIST1H3F and available mRNA Expression data were pooled, amounting to 143 patients. These patients originated from the following databases: Open Pediatric Brain Tumor Atlas (OpenPBTA), Pacific Pediatric Neuro-Oncology Consortium Clinical Genomics Atlas (PNOC, PBTA, Provisional), Pediatric Brain Tumor Atlas (PBTA, Provisional), Pediatric MiOncoSeq CNS Tumors (PBTA, Provisional), Proteomics (CBTTC, PBTA, Provisional), Brain Lower Grade Glioma (TCGA, PanCancer Atlas), Paediatric High Grade Glioma (HERBY Clinical Trial, Cancer Cell 2018), Paediatric High Grade Glioma (ICR London, Cancer Cell 2017). At the time of annotation in pedcBioPortal some of these samples were not recognized as H3K27M altered DMGs, but according to WHO classification of CNS tumors of 2021 they fall into this category (https://doi.org/10.1093/neuonc/noab106). ASCL1 RNA expression z-scores were split into quartiles and multi-group univariate LogRank test was calculated on pedcBioPortal excluding overlapping samples and patients.

### qPCR

Reverse transcription of total RNA was performed using oligo dT primers according to the manufacturer’s protocol (Maxima H Minus cDNA Synthesis Master Mix - Thermo Scientific, #M1661). qPCR was performed on a QuantStudio 3 Real-Time PCR System (Applied Biosystems) with SYBR Select Master Mix (Applied Biosystems, #4472908), under the following conditions: 95 °C for 15 s, 60 °C for 60 s (40 cycles), with the following oligos: MYT1 (F: TTCAGACCAGCGAAACCTCACC, R: TGTGGCTTCGTGCTGAGGTTCT), BCAN (F: TAAGCACAGCCGCTTCAACGTC, R: CTCTGTCACTGTGACGATAGCC), S100A11 (F: CCAGAAGTATGCTGGAAAGGATG, R: CATCATGCGGTCAAGGACACCA), ACTN1 (F: CAGGACCGTGTGGAGCAGATTG, R: CAGATTGTCCCACTGGTCACAG), IGFBP3 (F: CGCTACAAAGTTGACTACGAGTC, R: GTCTTCCATTTCTCTACGGCAGG), and GAPDH (F: GGAGCGAGATCCCTCCAAAAT, R: GGCTGTTGTCATACTTCTCATGG). Two biological replicates and four technical replicates were used, and the relative expression was normalized to the expression of housekeeping gene GAPDH.

### CUT&RUN

CUT&RUN (Cleavage Under Targets & Release Under Nuclease) was performed in biological duplicates as described in ref. ^90^ with minor modifications. 100,000 cells per reaction were used for histone modification CUT&RUN, and 500,000 cells were used for non-histone targets. For each reaction, cells were added to 10 µL Concanavalin A beads (Bangs Laboratories, BP531). Primary antibodies (1:100 anti-ASCL1, Abcam ab74065; 1:100 anti-H3K27me3, Cell Signaling Technology #9733; 1:100 anti-H3K27M, Abcam ab190631; 1:100 anti-BRG1, Cell Signaling Technology #49360) were added and nutated overnight at 4 °C. 50 µL of protein AG-Mnase (in-house purified) was added per reaction. Mnase digestion was performed at 4 °C for 30 min for histone modifications and 2hrs for non-histone targets. Spike-in DNA for sample normalization from *S. cerevisiae* (Cell Signaling Technology) was included in the STOP buffer and added to a final concentration of 0.5 pg/µL. Target-enriched DNA fragments were purified using Monarch PCR & DNA Cleanup columns (NEB T1030S). DNA was quantified by Qubit prior to library preparation. Libraries were prepared using NEB Ultra II DNA Library Prep Kit for Illumina (E7645L) according to the manufacturer’s protocol. Size selection was performed using Ampure XP beads (Beckman Coulter A63881) and libraries were quantified by Qubit. 50×50 paired-end sequencing was performed on a Nextseq 2000.

### CUTAC

CUTAC (Cleavage Under Targeted Accessible Chromatin) was performed in biological duplicates as described in ref. ^69^ with minor modifications. In brief, nuclei from fresh cell cultures were isolated in NE1 buffer (20 mM HEPES pH 7.9, 10 mM KCl, 0.5 mM spermidine, 0.1% Triton-X-100, 20% glycerol, one Roche protease inhibitor tablet, EDTA-free, per 50 mL buffer) without fixation, and cryopreserved in 10% DMSO in Wash Buffer (20 mM HEPES pH 7.9, 150 mM NaCl, 0.5 mM spermidine, one Roche protease inhibitor tablet, EDTA-free, per 50 mL buffer). For each CUTAC reaction, 50,000 nuclei were thawed and added to 3.5 µL of Concanavalin A beads (Bangs Laboratories, BP531). Samples were nutated in 1:75 dilutions of primary antibody (anti-H3K4me2, Sigma-Aldrich 07-030) overnight at 4 °C in 30 µL reaction volumes. Secondary antibody incubations (1:100 guinea pig anti-rabbit IgG, Novus Biologicals NBP1-72763) were performed for 1 h at room temperature. For transposase binding, 0.3 µL of pA-Tn5 (purified in-house) pre-loaded adapter complex was added to each reaction and nutated at room temperature for 1.5h. Tagmentation was performed in Wash buffer containing 0.05% Triton-X-100, 5 mM MgCl_2_, and 10 mM TAPS buffer, for 20 min at 37 °C in a thermocycler. PCR amplification of released DNA fragments was performed with non-hot-start polymerase (NEB M0541S) using the following program: 58 °C, 5 min; 72 °C, 5 min; 98 °C 30 s; (98 °C 10 s, 63 °C 30 s) x 12 cycles total, 72 °C, 1 min, with primers described in ref. ^91^. Size selection was performed using Ampure XP beads (Beckman Coulter A63881) and libraries were quantified by Qubit. 50×50 paired-end sequencing was performed on a Nextseq 2000.

### CUT&Tag

CUT&Tag was performed in biological duplicates according to^69^ with minor modifications for profiling H3K27me3 genomic occupancy. Aliquots of 50,000 nuclei were used per reaction. Primary antibody (1:75 anti-H3K27me3, Cell Signaling Technology #9733) was added and nutated overnight at 4 °C. Secondary antibody incubations (1:100 guinea pig anti-rabbit IgG, Novus Biologicals NBP1-72763) were performed for 1 h at room temperature. For transposase binding, 0.3 µL of pA-Tn5 (purified in-house) pre-loaded adapter complex was added to each reaction and nutated at room temperature for 1.5 h. Tagmentation was performed for 1 h at 37 °C. No spike-in DNA was added. Primers used for indexing are described in ref. ^91^.

### RNA-seq

Total RNA was extracted from 0.5 to 1.5 million cells using Direct-zol RNA Miniprep Plus columns (R2072) with on-column Dnase I treatment according to the manufacturer’s protocol. DMG cells grown in TSM were processed in biological triplicates. DMG cells grown in DM were processed in biological duplicates. Oligo dT-based mRNA purification was performed using NEBNext Poly(A) mRNA Magnetic Isolation Module (E7490L), starting from 200 to 500 ng of total RNA. Non-stranded cDNA libraries were prepared using NEBNext Ultra II RNA Library Prep Kit for Illumina (E7770L) according to the manufacturer’s protocol. Size selection was performed using Ampure XP beads (Beckman Coulter A63881) and libraries were quantified by Qubit. 50×50 paired-end sequencing was performed on a Nextseq 2000.

### RNA-seq analysis

Fastq files were aligned to the hg38 genome using STAR v2.7.9a to generate sorted bam files. Bam files were sorted and indexed using samtools v1.8. Gene counts were generated using featureCounts from the Rsubread v2.6.1 package, using the inbuilt hg38 genome annotation and default parameters. Differential gene expression was performed with DESeq2 v1.32.0. *P*-values were adjusted using the Benjamini–Hochberg procedure. Genes synergistically dysregulated in DKOs at Day3 were defined as those having greater-than-additive effects in DKOs, e.g., for upregulated genes, (Log2FoldChange(ASCL1-KO vs. Con)) + Log2FoldChange(K27M-KO vs. Con) <Log2FoldChange(DKO vs. Con), while also passing the significance threshold in DKOs (abs(Log2FoldChange) > 1 & adjusted *p*-value < 0.05).

### GSEA analysis for lineage programs

To evaluate developmental lineage programs in the bulk RNA seq samples, a panel of gene signatures (*length* = *100 genes)*, was assembled using an scRNA-seq atlas spanning mouse pontine development from embryonic day 10.5 to postnatal day 6, as reported in the original studies^29,36^. Signatures for human second trimester thalamus (*length* = *100 genes)*, was generated from the cell type marker genes reported in the original publication^92^. Marker genes were filtered for genes with an adjusted *p*-value < 0.05 and a positive fold change, and then the top 100 genes per cell type ranked by fold change were selected for the signature. Gene set enrichment analysis (GSEA) was performed using the R-package ClusterProfiler v4.0.0^93^ with the lineage signatures as input, ranking genes by the negative binomial Wald test statistic (DESeq2 v1.22.2)^94^. *P*-values were adjusted using the Benjamini–Hochberg procedure and signatures with adjusted *p*-value < 0.05 were considered significantly enriched or depleted.

### Expression of ASCL1 in single cell atlas

Processed and annotated first trimester and adult pons and thalamus data was downloaded from the original publication (https://github.com/linnarsson-lab/developing-human-brain, https://storage.cloud.google.com/linnarsson-lab-human/adult_human_20221007.loom), and randomly down sampled to max 5000 cells per cell type for visualization^95,96^. Processed and annotated single cell data of the developing human thalamus^92^ was downloaded from UCSC cell browser (http://cells.ucsc.edu/?ds=dev-thal)^92^. The single cell atlas of developing mouse pons was processed and annotated as described in original studies^29,36^. For each reference the average normalized expression was calculated per cell type and scaled between 0-1 and the detection rate was calculated as the percentage of cells with expression >0 per cluster.

### Expression of ASCL1 in single cell tumor samples

Single cell tumor samples were processed and annotated as described in original study^36^. Normalized expression of *ASCL1* was visualized per tumor entity and projected cell type.

### Expression of ASCL1 in bulk tumor samples

Bulk RNA-seq samples from high grade gliomas, low grade gliomas and ependymomas were collected from previous publications^29,36,58,61,97,98^. Samples were processed as previously described^36^.

### GBM gene signatures

Neural progenitor-like and mesenchymal-like genes were obtained from the NPC1 and MES1 metamodules from ref. ^9^. Gene signature scores were calculated as the average relative expression (z-score) of the metamodule for each sample at Week 10 in DM.

### Super enhancer analysis

Super enhancers (SEs) in DIPG-XIII grown as gliomaspheres in TSM were previously identified based on H3K27Ac enrichment in ref. ^67^. SE coordinates (originally in hg19) were lifted to hg38 using CrossMap.py. The overlap between the top 15% (*n* = 1018) of ASCL1 peaks in Week 10 DM spheres (ranked by MACS2 peaks score) and SEs was obtained using the subsetByOverlaps function from the GenomicRanges (v1.46.1) package.

### CUTAC analysis

Paired-end reads were aligned to hg38 using Bowtie2 v2.3.5.1 with parameters --no-unal --local --very-sensitive-local --no-mixed --no-discordant --phred33 -I 10 -X 700. Bam files were sorted and indexed using samtools v1.8. Bigwig track files were created using bamCoverage from deepTools v3.3.1, with settings --binSize=20 --smoothLength=60 --normalizeUsing BPM. No spike-in normalization was performed for CUTAC. Peaks were called for each sample individually using MACS2 v2.6 with default parameters, using an IgG CUT&Tag sample as the control. A merged CUTAC peakset across all samples was generated using the count function in Diffbind v3.2.7 with default parameters, keeping only peaks called in at least two samples. featureCounts (Rsubread v2.6.1) was used to count CUTAC reads mapping to the consensus peakset, and differential enrichment was performed with DESeq2 v1.32.0. *P*-values were adjusted using the Benjamini–Hochberg procedure. CUTAC peaks uniquely up/downregulated in DKOs at Day3 were defined as those peaks passing the threshold of abs(Log2FoldChange) > 1 & adjusted *p* value < 0.05 in DKOs but not single KOs vs. Control. Unchanged peaks were defined as those with abs(log2FoldChange) < 1 & adjusted *p* value > 0.1.

### CUT&RUN and CUT&Tag analysis

For CUT&RUN, reads were aligned to the human hg38 (target genome) and Saccharomyces cerevisiae sacCer3 (spike-in genome) using Bowtie2 v2.3.5.1, with settings --no-unal --local --very-sensitive-local --no-mixed --no-discordant --phred33 -I 10 -X 700. Bam files were sorted and indexed using samtools v1.8. Spike-in-normalized bigwig track files were created using bamCoverage from deepTools v3.3.1, with settings --binSize=20 --smoothLength=60 --normalizeUsing BPM --scaleFactor $scale_factor, where $scale_factor = the ratio of total reads / spike-in reads. For H3K27me3 CUT&Tag, data was similarly processed as above except the E. coli genome (K-12 MG1655) was used as the spike-in genome reference. Bigwig files from replicate experiments were averaged and merged for visualization using the bigWigMerge and bedGraphToBigWig commands from the UCSC Genome Browser’s utilities.

For BRG1 CUT&RUN, peaks were called on individual samples with MACS2 v2.6 with default parameters, using an IgG CUT&RUN sample as the control. A merged BRG1 peakset was generated using the count function in Diffbind v3.2.7 with default parameters, keeping only peaks called in at least two samples. featureCounts (Rsubread v2.6.1) was used to count reads mapping to the consensus peakset. Counts were normalized to spike-in reads by multiplying the counts for each sample by the ratio of total reads/spike-in reads. The spike-in-normalized (but not read depth-normalized) counts table was used as input for differential enrichment analysis by DESeq2 v1.32.0. *P*-values were adjusted using the Benjamini–Hochberg procedure. BRG1 peaks significantly up/downregulated in DKOs at Day3 were defined as those peaks with abs(Log2FoldChange) > 1 & adjusted *p* value < 0.05 vs. Day3 Controls. Unchanged peaks were defined as those with abs(log2FoldChange) < 1 & adjusted *p* value > 0.05.

For H3K27me3 peaks in Con Sph and Con Adh at Wk10 DM, peaks were called on merged replicates with default (narrowPeak) settings in MACS2 v2.6 (narrowPeak because the oncohistone mutation restricts H3K27me3 spreading), using an IgG CUT&RUN sample as the control. Peaks called in Con Sph and Con Adh were merged using the BEDTools (v2.28.0) merge function. featureCounts (Rsubread v2.6.1) was used to count reads mapping to the merged peakset. Counts were scaled according to CUT&RUN scaling factors and differential enrichment was performed using DESeq2 v1.32.0, with significantly differential enrichment defined as abs(Log2FoldChange) > 1 and adjusted *p*-value < 0.05.

For ASCL1 CUT&RUN, replicates were pooled and MACS2 v2.6 was used with default parameters for peak calling, using ASCL1 CUT&RUN in ASCL1-KO as the control. A merged set of 13,034 ASCL1 peaks from Day0 (TSM) and Day3 DM was generated using the union function from the GenomicRanges (v1.46.1) package. These peaks were intersected with uniquely reduced CUTAC peaks (*n* = 4188) in DKO vs. Control at Day 3, resulting in 1865 peaks. These were further intersected with reduced BRG1 peaks in DKO vs. Control at Day 3, resulting in 925 peaks.

### Peak annotation and motif analysis

Peak annotation was performed using the annotatePeaks.pl script from Homer v4.11, using a custom GTF containing only coding genes. This was generated from the UCSC hg38 genes.gtf file by filtering out non-coding RNAs using the grepl function to keep only annotations containing “NM_” (mRNA). Known and de novo sequence motif enrichment was performed using the findMotifsGenome.pl from Homer v4.11. Gene ontology analysis of peaks with re-distributed BRG1 and chromatin accessibility was performed using GREAT^99^.

### Bigwig data visualization

IGV versions 2.11.9 and 2.19.4 were used to visualize bigwig track data.

### Gene set enrichment analysis

GSEA analysis was performed using the msigdbr (v7.5.1) R package, which provides the Molecular Signatures Database (MSigDB) gene sets. Differentially expressed genes were ranked by Wald statistic. The clusterProfiler (v4.0.5) R package was used to perform gene ontology analyses to identify overrepresented biological processes.

### Single-cell RNA + ATAC-seq and analysis

Control and ASCL1-KO BT245 cells were collected at Day 6 in DM. ~4000 nuclei per sample were loaded onto the Chromium X (10X Genomics), and gel beads in emulsion (GEMs) were processed for single-nuclear RNA + ATAC-seq according to the manufacturer’s protocol (Chromium Next GEM Single Cell Multiome ATAC + Gene Expression Rev F) using 7 cycles for cDNA amplification and 11 cycles for library amplification. Samples were sequenced on a Nextseq 2000 at targeted ~25,000 reads per cell. Count matrices were generated by mapping reads to hg38 using CellRanger-ARC (v2.0.0) and SAMtools (v1.18). Downstream processing was performed in Seurat v5. Filtering: Cells with >20% of transcripts derived from mitochondria, <1000 or >7000 genes detected were removed. Cells with less than 1000 or more than 30,000 ATAC peaks were removed, as were cells with a TSS enrichment score of <2 or a nucleosome signal >1.5. Downstream analyses were performed on *n* = 2245 Control and 1806 ASCL1-KO cells. Cell clusters were identified using the Louvain algorithm with FindClusters (resolution = 0.5). A UMAP embedding was generated using the RNA assay with RunUMAP (reduction = “pca”, dims 1:30). For integration of ATAC data, integration anchors were identified with FindIntegrationAnchors (reduction = “rlsi” and dims = 2:30).

To plot gene module scores, NPC1, MES1, and G2M gene modules were obtained from Neftel et al., 2019. Module scores were visualized on a UMAP embedding using Seurat’s FeaturePlot (blend = true, threshold = 0.5). To plot chromatin accessibility scores, DNA motifs were obtained using the getMatrixSet command from TFBSTools (v1.40) (collection = “CORE”, tax_group = ‘vertebrates’, all_versions = FALSE) from the JASPAR 2020 database (v0.99.10)^100^.

### RNA velocity analysis

We inferred transcriptional dynamics by RNA velocity analysis using scVelo (v.0.3.3)^101^ implemented in Python (v3.9.23), according to the developers’ tutorial. Loom files were generated from BAM files output by Cell Ranger using velocyto (v.0.17.17)^102^. RNA velocity analysis was performed in stochastic mode on the top 2000 highly variable genes with default settings.

### Mouse orthotopic xenografts

For DIPG-XIII Control and ASCL1-KO cells expressing luciferase, 10 million cells were seeded in flask, and the following day, cells were resuspended in 45 µL of matrigel. 5 µl was injected into each animal (Control, *N* = 7; ASCL1-KO, *N* = 6) (athymic 5-6 week old females, Charles Rivers Labs), using a manual stereotaxic frame, into the fourth ventricle. Tumors were monitored using the IVIS Spectrum In Vivo Imaging System.

For generation of xenograft survival data, mice were housed, bred, and handled according to procedures approved by the McGill University Health Center Animal Care Committee and in accordance with the Canadian Council on Animal Care guidelines. Mice were maintained in a pathogen-free facility under a 12-h light/dark cycle with ad libitum access to food and water and were group-housed (up to 5 animals per cage) with environmental enrichment.

Female NOD.Cg-Prkdcscid Il2rgtm1Wjl/SzJ (The Jackson Laboratory, RRID: IMSR_JAX:005557) mice, aged 4–6 weeks, were used for orthotopic xenograft experiments. Sex/gender analysis was not performed because this study focused on the molecular mechanisms of tumor-intrinsic cell plasticity. Only female mice were used to reduce variability associated with sex-specific differences in behavior and housing, as female mice can be group-housed with reduced aggression and stress compared to males, therefore improving experimental consistency.

Mice were injected with the intended cell lines at a concentration of 7 × 10^5^ cells in the pons for DIPGXIII: (1) DIPGXIII unedited cells (*n* = 5), (2) DIPGXIII ASCL1-KO (*n* = 5), and in the caudate putamen for BT245: (1) BT245 unedited cells (*n* = 5), (2) BT245 ASCL1-KO (*n* = 5). Stereotaxic injections were performed using the Neurostar Robot Stereotaxic system.

Maximum tumor burden for intracranial xenografts was defined by humane endpoint criteria rather than a predefined tumor volume due to the intracranial location. Mice were monitored at least 2–3 times per week, and more frequently after the onset of symptoms. Animals were euthanized upon presentation of neurological or systemic signs including ≥15–20% body weight loss, lethargy, impaired mobility or gait abnormalities, seizures, hydrocephalus, dehydration, or inability to access food or water.

Euthanasia was performed using isoflurane anesthesia followed by carbon dioxide asphyxiation, with cervical dislocation as a secondary physical method to ensure death, in accordance with institutional and CCAC guidelines. Brains were collected and fixed in 10% formalin for histological analysis.

Kaplan–Meier survival plots were generated using GraphPad Prism software. Mice that succumbed to tumor burden were assigned a value of 1, whereas mice that survived until the end of the experiment or were euthanized for reasons unrelated to tumor burden were censored (value of 0).

### Statistics and reproducibility

Statistical analyses were performed using Prism 9/10 (GraphPad). Error bars were plotted as mean ± SD unless otherwise noted. No statistical method was used to predetermine sample sizes. Sequencing experiments which failed at QC were excluded from analyses. CRISPR screens, CUT&Tag for H3K27me3, CUT&RUN for ASCL1, BRG1, and K27M, and CUTAC experiments were performed as *n* = 2 biological replicates. Bulk RNA-seq from cells grown in DM were performed as *n* = 2 biological replicates. Bulk RNA-seq from cells grown in TSM were performed as *n* = 3 biological replicates. scRNA-seq analyses were performed on *n* = 2245 (control) and *n* = 1806 (ASCL1-KO) BT245 cells. Experiments were not randomized and investigators were not blinded to allocation during experiments and outcome assessment.

### Reporting summary

Further information on research design is available in the Nature Portfolio Reporting Summary linked to this article.

## Supplementary information

Supplementary Information

Description of Additional Supplementary Information

Supplementary Data 1

Supplementary Data 2

Reporting Summary

Transparent Peer Review file

## Source data

Source Data

## Data Availability

The sequencing data generated in this study have been deposited in the Gene Expression Omnibus (GEO) database under accession codes GSE269554 (https://www.ncbi.nlm.nih.gov/geo/query/acc.cgi?acc=GSE269554) (chromatin accessibility by CUTAC), GSE269555 (https://www.ncbi.nlm.nih.gov/geo/query/acc.cgi?acc=GSE269555) (CUT&RUN), GSE269556 (https://www.ncbi.nlm.nih.gov/geo/query/acc.cgi?acc=GSE269556) (CUT&Tag), GSE269557 (https://www.ncbi.nlm.nih.gov/geo/query/acc.cgi?acc=GSE269557) (bulk RNA-seq), and GSE326427 (https://www.ncbi.nlm.nih.gov/geo/query/acc.cgi?acc=GSE326427) (single-cell RNA + ATAC-seq). The publicly available data used in this study are available in the European Genome-phenome Archive (EGA) under accession code EGAS00001004301^58^, EGAS00001007510^60^, EGAS00001003572^61^, EGAS00001002696^98^, EGAS00001004107^96^; under dbGaP accession number phs002380.v1.p1^97^; on the UCSC cell browser at http://cells.ucsc.edu/?ds=dev-thal^92^, the Gene Expression Omnibus (GEO) under accession code GSE133531^29^, GSE188625^36^, GSE131928^9^, GSE184357^42^, GSE94259^67^; and the BRAIN Initiative Cell Census Network under accession code SCR_015820^95^. The remaining data are available within the Article, Supplementary Information or Source Data file. Source data are provided with this paper.
