# Supplementary material for: H3K27M cooperates with ASCL1 to maintain progenitor states and drive gliomagenesis
Source: Nat Commun. 2026 Oct 10;17:11510. doi: 10.1038/s41467-026-78138-z (PMC13745522; doi:10.1038/s41467-026-78138-z)
Supplement: Supplementary file 1 [file 41467_2026_78138_MOESM1_ESM.pdf]

H3K27M cooperates with ASCL1 to maintain progenitor states and drive gliomagenesis

Supplementary Figures

Supplementary Figure 1. CRISPR screen metrics.

Supplementary Figure 2. ASCL1 promotes K27M-driven gliomagenesis.

Supplementary Figure 3. ASCL1 expression in neural development.

Supplementary Figure 4. ASCL1 expression in high grade glioma.

Supplementary Figure 5. ASCL1 regulates unique gene expression programs in K27M-DMGs.

Supplementary Figure 6. Epigenetic memory of serum treatment.

Supplementary Figure 7. ASCL1 and K27M promote RGC/OPC-like states and repress MES-like states.

Supplementary Figure 8. ASCL1 is necessary and sufficient to drive NPC programs.

Supplementary Figure 9. A MES-like subpopulation arises without genetic manipulation.

Supplementary Figure 10. ASCL1 and K27M cooperatively regulate chromatin accessibility.

Supplementary Figure 11. K27M-KO restores H3K27me3 and reduces H3K27Ac in BT245.

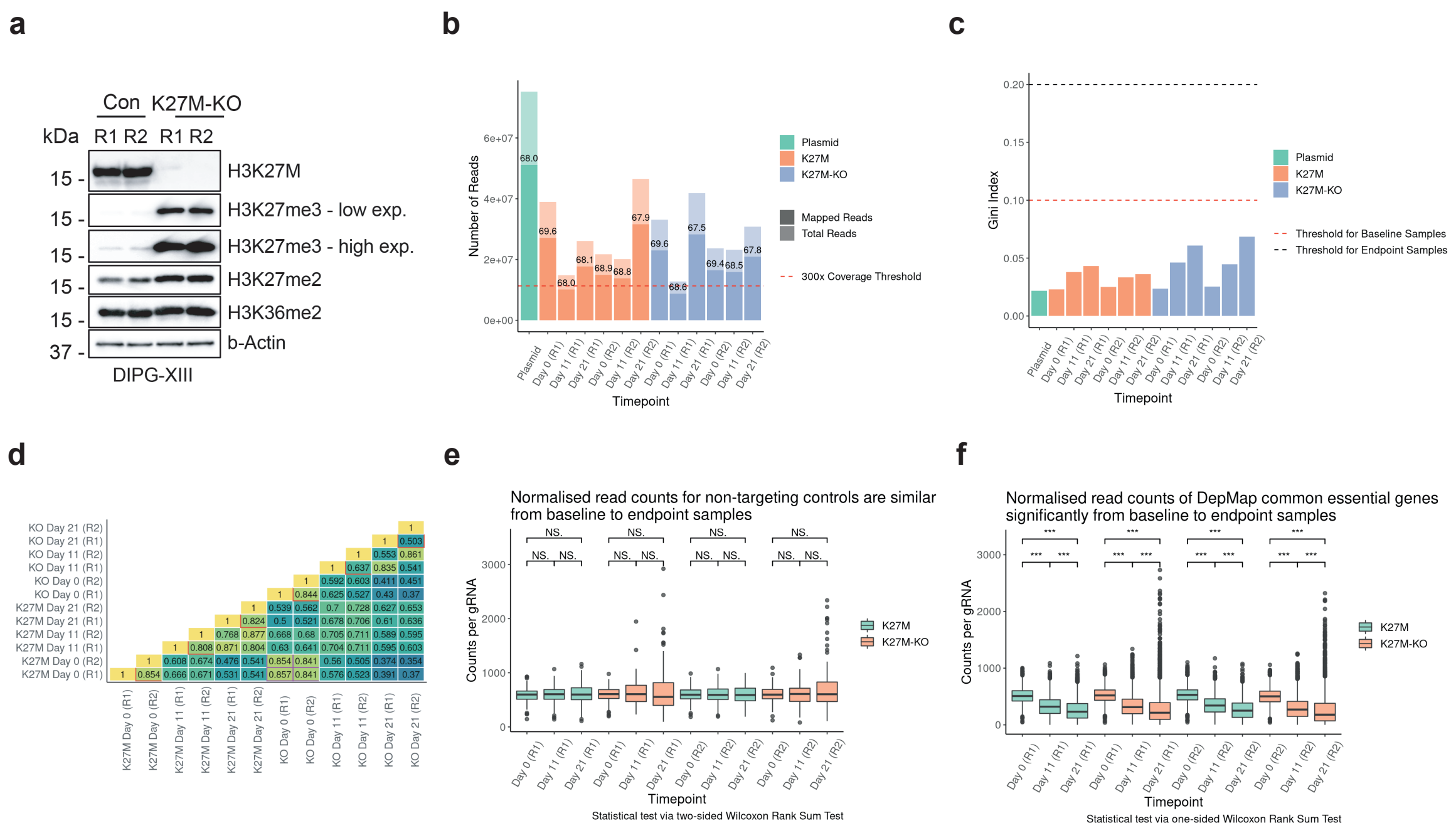

Supplementary Figure 1. CRISPR screen metrics.

(**a**) Western blot demonstrating H3K27M deletion in DIPG-XIII cells. Con = Control (non-targeting) guide RNA-expressing cells. R: technical replicates. (**b**) Number of mapped/unmapped reads per sample. Plasmid refers to the amplified MinLibCas9 plasmid library used for transfections. R1 = replicate 1, R2 = replicate 2. (**c**) Gini index (measure of the evenness of gRNA read counts per sample) values for each sample. Low Gini index indicates even distribution of read counts over all gRNAs within each sample. Quality control thresholds applied as per [1]. R: Biological replicate. (**d**) Pairwise Pearson correlation matrix across all samples with correlation coefficients labelled and correlations between biological replicates (red-lined) and between baseline (Day 0) samples (purple-lined). (**e, f**) Boxplot of counts across all samples for the 200 non-targeting negative control gRNAs of the MinLibCas9 library (e) and for gRNAs targeting Cancer Dependency Map (DepMap) common essential genes (f). Bounds of box indicate 25th and 75th percentiles. Centre line indicates median; whiskers indicate Q1-1.5*(interquartile range) and Q3+1.5*(interquartile range). Statistical comparison performed via two-sided and one-sided Wilcoxon Rank Sum tests respectively. Common essential genes sourced from DepMap Q1/22 release. R: Biological replicate, NS.: Non-Significant, ***: P < 0.001. All CRISPR screen metrics are based on independent biological duplicate screens. Source data are provided as a Source Data file.

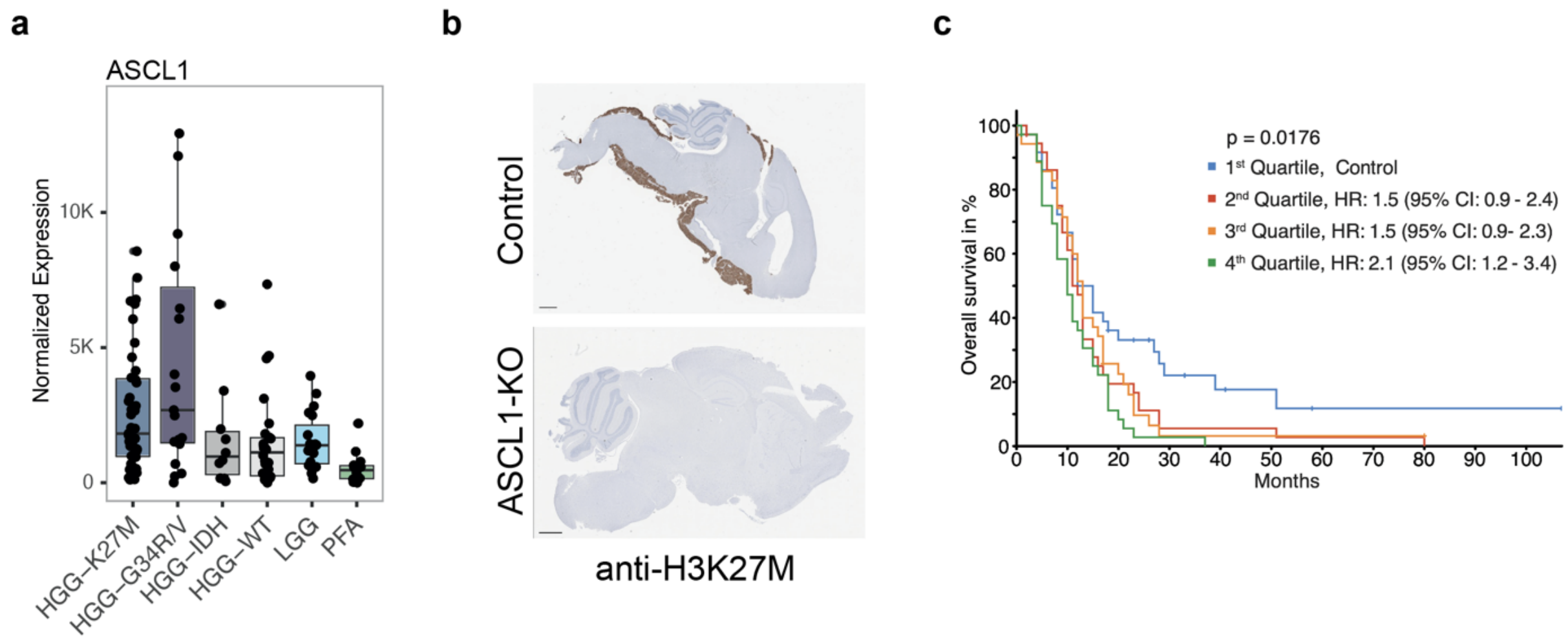

Supplementary Figure 2. ASCL1 promotes K27M-driven gliomagenesis.

(**a**) Normalized expression of *ASCL1* in (n=132) bulk RNA-seq tumour samples. HGG: high grade glioma, LGG: low grade glioma, PFA: posterior fossa ependymoma type A. Bounds of box indicate 25th and 75th percentiles. Centre line indicates median; whiskers indicate Q1-1.5*(interquartile range) and Q3+1.5*(interquartile range). Data beyond whiskers are plotted individually. (**b**) Immunohistochemistry for H3K27M in DIPGXIII-luciferase xenografts 100 days post-injection. Scale bar = 1mm. Similar results were obtained from n=5 mice per condition. (**c**) Kaplan–Meier survival analysis for ASCL1 expression split by quartiles for 143 patients (HR, hazard ratio; p value according to multi-group univariate LogRank test). Source data are provided as a Source Data file.

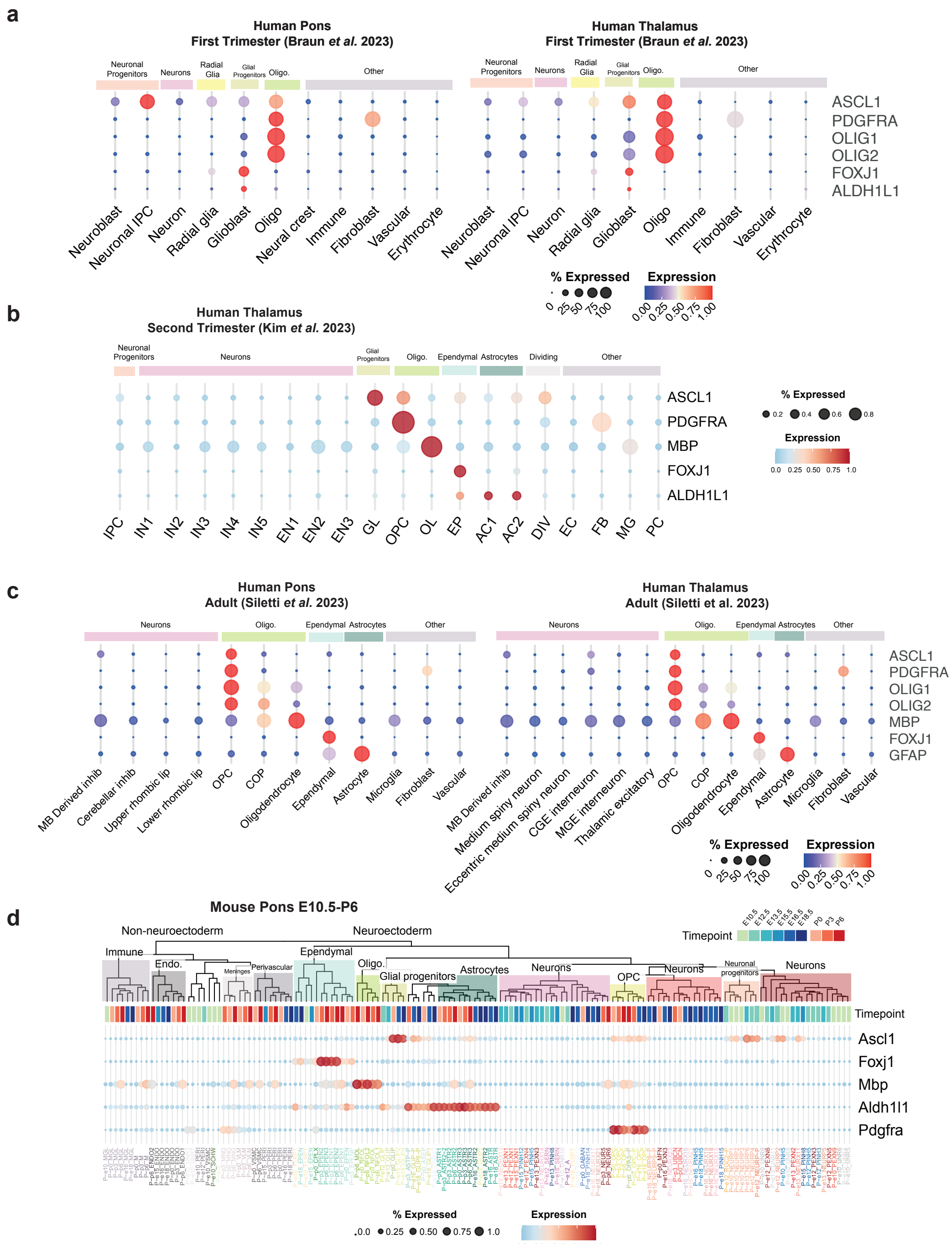

Supplementary Figure 3. *ASCL1* expression in neural development.

(**a**) Average expression and detection rate of *ASCL1* and cell type markers *PDGFRA, OLIG1, OLIG2* (oligodendrocyte progenitor cells), *FOXJ1* (ependymal), and *ALDH1L1* (astrocytes) in developing human pons (n=27084 cells) and thalamus (n=24124 cells). First trimester: gestational week 5-14[2]. (**b**) Average expression and detection rate of *ASCL1* and cell type markers *PDGFRA*

(oligodendrocyte progenitor cells), *MBP* (oligodendrocytes), *FOXJ1* (ependymal), and *ALDH1L1* (astrocytes) in developing human thalamus (second trimester: gestational week 16-25) (n=137007 cells). **(c)** Average expression and detection rate of *ASCL1* and cell type markers *PDGFRA, OLIG1, OLIG2* (oligodendrocyte progenitor cells), *MBP* (oligodendrocytes) *FOXJ1* (ependymal), and *ALDH1L1* (astrocytes) in adult human pons (n=38224 cells) and thalamus (n=46694 cells)[3]. (**d**) Average expression and detection rate of *Ascl1* and cell type markers *Foxj1* (ependymal), *Mbp* (oligodendrocytes), *Aldh1l1* (astrocytes), and *Pdgfra* (oligodendrocyte progenitor cells) in developing mouse pons (n=47907 cells; embryonic day 10.5- postnatal day 6).

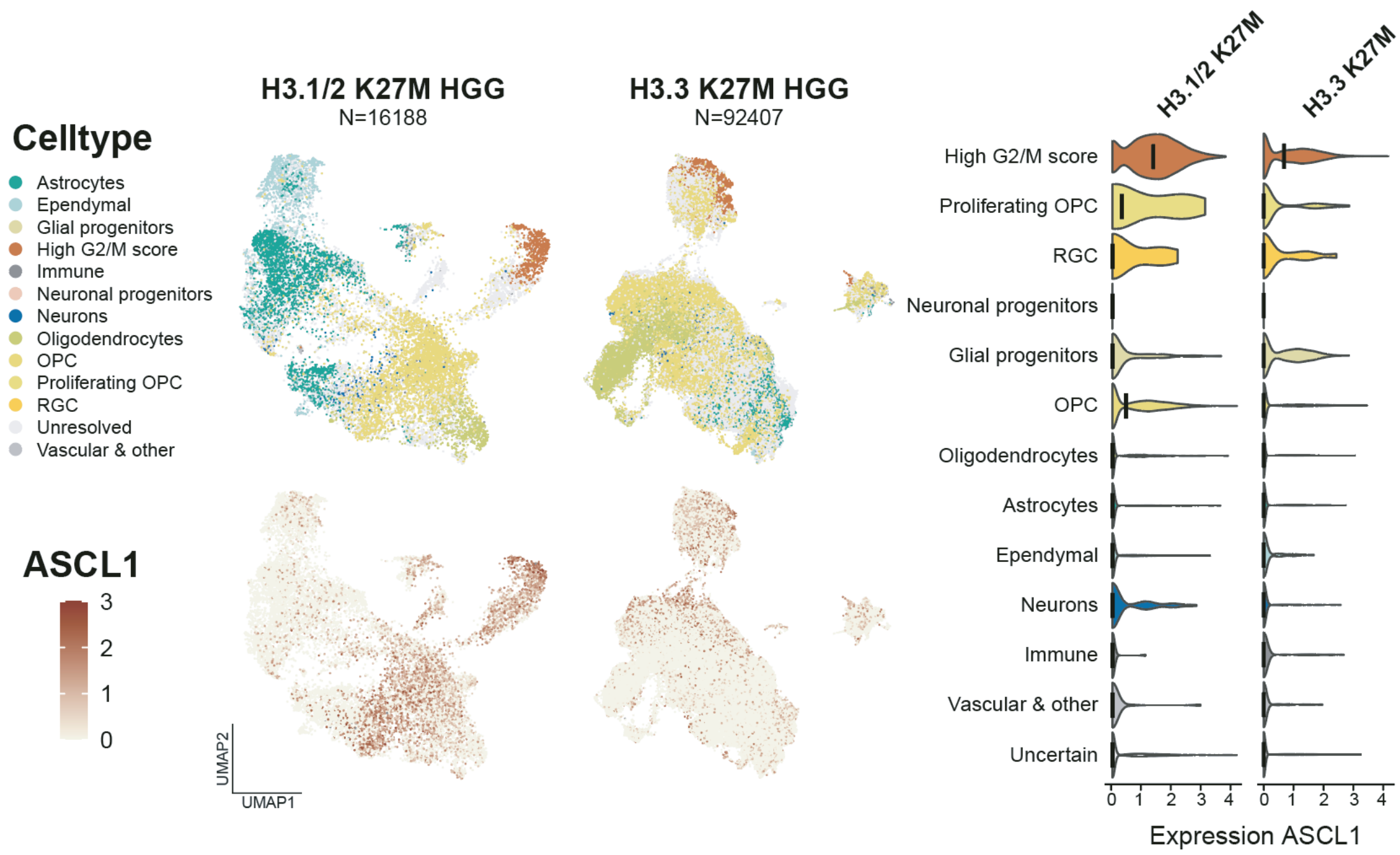

Supplementary Figure 4. *ASCL1* expression in high grade glioma.

UMAP for each tumor type: high grade glioma with H3.1/2 K27M (n=16188 cells), high grade glioma H3.3 K27M and posterior fossa ependymoma type A (n=92407 cells). Top: projected cell types from a normal developmental brain reference. Bottom: normalized expression of *ASCL1*. Right: Normalized expression of *ASCL1* per projected developmental cell type and tumour entity.

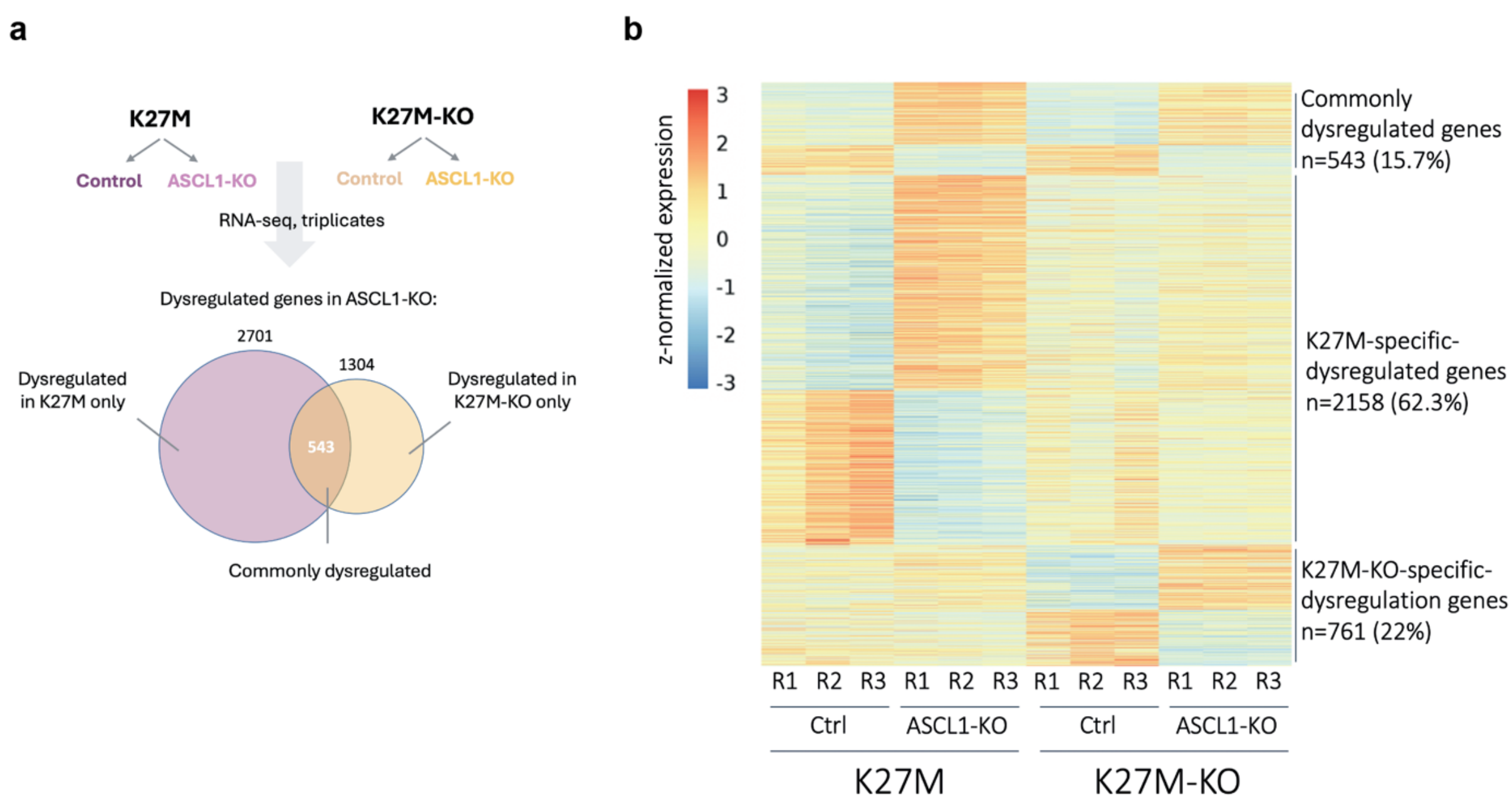

Supplementary Figure 5. ASCL1 regulates unique gene expression programs in K27M-DMGs.

(**a**) Schematic of RNA-seq conditions and Venn diagrams indicating number and overlap of genes significantly dysregulated (abs(Log2FoldChange) > 0.3 and padj < 0.05) upon ASCL1-KO in either K27M or K27M-KO DIPG-XIII cells. (**b**) Heat map of unique and shared differentially regulated genes upon ASCL1-KO in either K27M or K27M-KO DIPG-XIII cells. Percentages indicate % of total dysregulated genes following ASCL1-KO. R: Biological replicate. RNA-seq was performed as n=3 independent biological replicates.

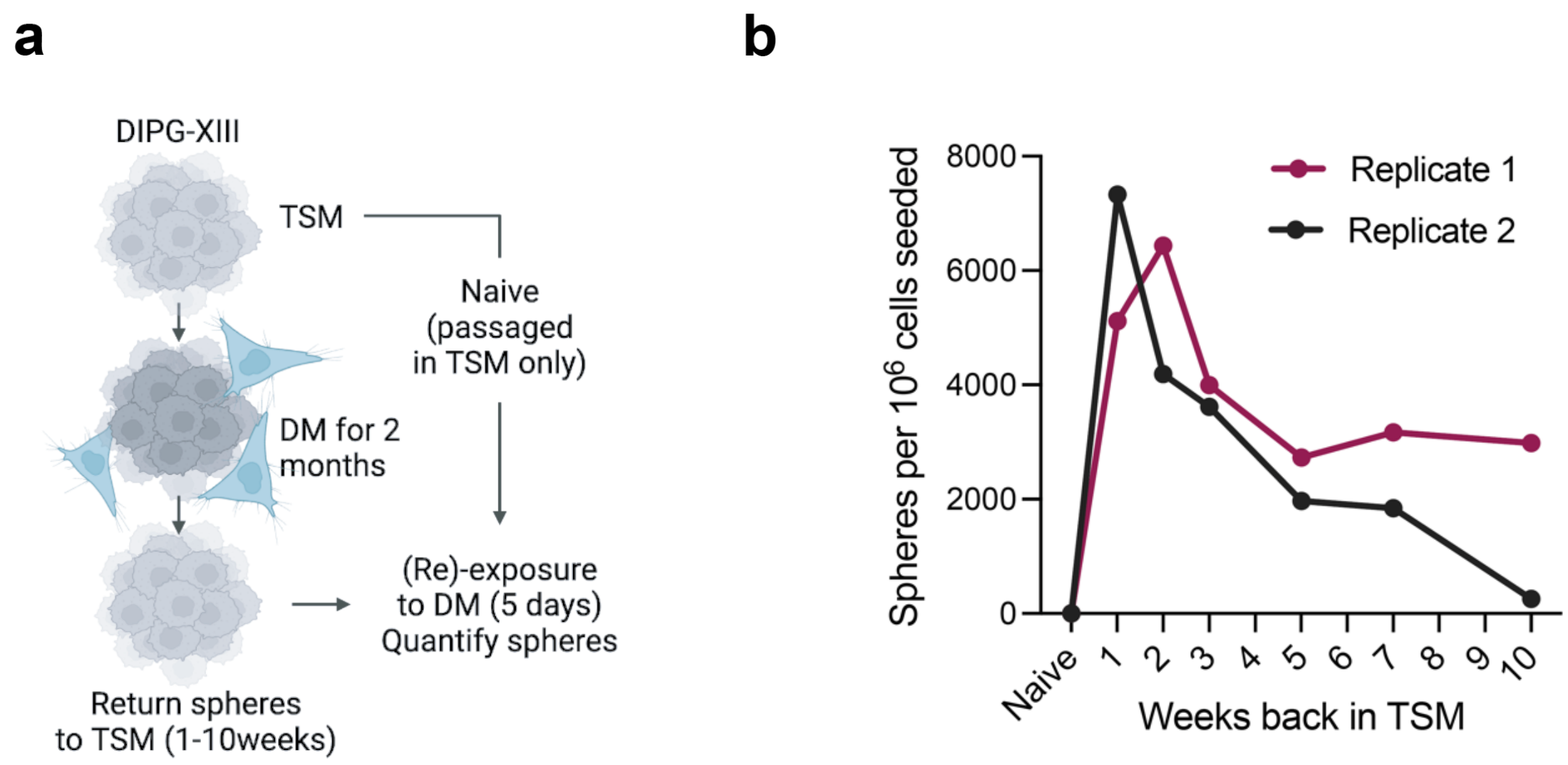

Supplementary Figure 6. Epigenetic memory of serum treatment.

(**a**) Schematic to test functional memory of gliomasphere formation upon a second exposure to DM. After 2 months in DM (first exposure), spheres were returned to TSM for variable amounts of time before dissociation and re-seeding in DM (second exposure). Created in BioRender. Jiao, A. (2026) https://BioRender.com/qlz6dij. (**b**) Quantification of gliomaspheres after 5 days of the second exposure in DM. n=2 biological replicates are shown individually. Source data are provided as a Source Data file.

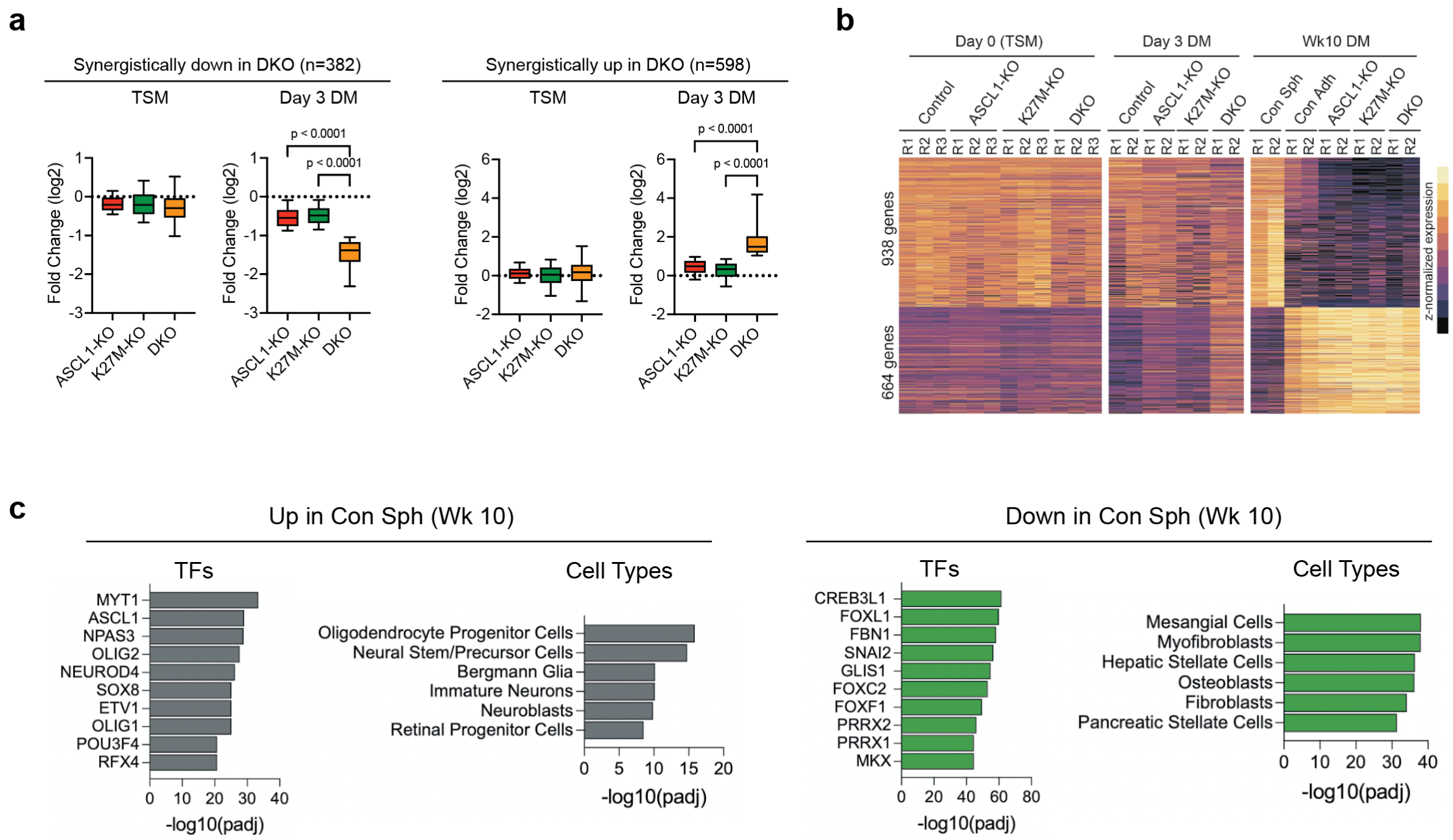

Supplementary Figure 7. ASCL1 and K27M promote RGC/OPC-like states and repress MES-like states.

(**a**) Relative expression of cooperatively (greater than additive effects of single KOs) up/downregulated genes in DIPG-XIII D3 DKO vs. D3 Control. Bounds of box indicate 25th and 75th percentiles. Centre line indicates median; whiskers indicate 10th and 90th percentile of n=382 and n=598 genes. Statistic: one-way ANOVA. Dotted line indicates expression level in Control. n=3 independent biological replicates in TSM and n=2 independent biological replicates in DM. (**b**) Heat map of gene expression in TSM, and Day3 and Wk10 in DM. 938 genes were significantly downregulated at least 4-fold in all three KO lines at Wk10 relative to Con Sph. 664 genes were significantly upregulated at least 4-fold in all three KO lines at Wk10 relative to Con Sph. Statistical significance: adjusted p-value < 0.05. R: replicate. n=3 independent biological replicates in TSM and n=2 independent biological replicates in DM. (**c**) Enrichr analysis of the genes up- and downregulated in DM spheres, respectively, relative to all KO lines at Week10 DM. RNA-seq was performed as n=2 independent biological replicates. The top n=100 most highly expressed genes in each set were used for the analysis.

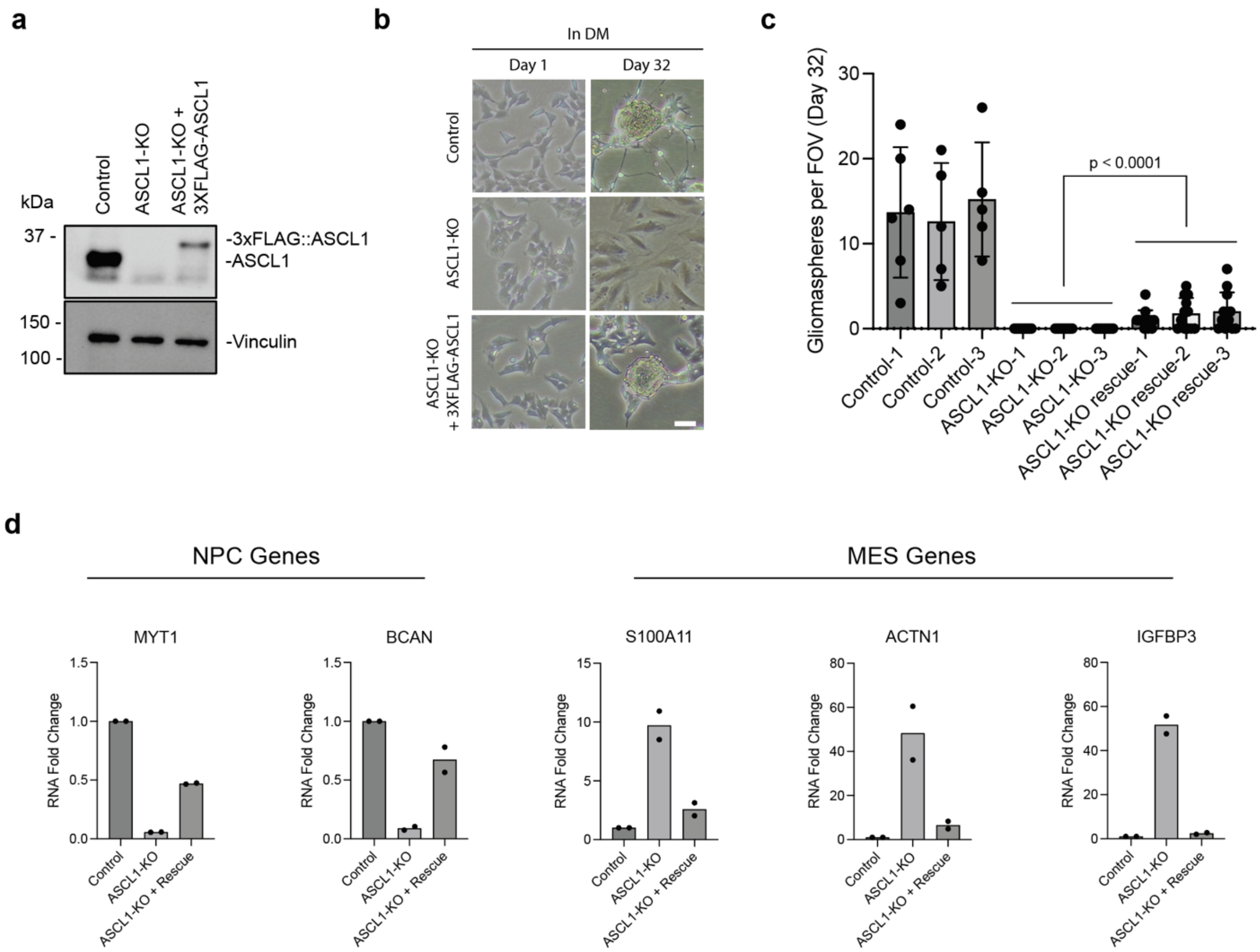

Supplementary Figure 8. ASCL1 is necessary and sufficient to drive NPC programs.

(**a**) ASCL1 Western blot confirms expression of 3XFLAG::ASCL1 rescue construct in ASCL1-KO DIPGXIII. (**b**) Light micrographs of DIPG-XIII following serum-induced differentiation. Gliomaspheres stably expressing 3xFLAG::ASCL1 were generated in TSM prior to dissociation and seeding in DM. Bar = 50um. Images are representative of n=3 independent experiments quantified in (c). (**c**) Gliomasphere quantification per field of view (FOV) at Day 32 in DM. Statistic: two-tailed t-test (n=3 independent biological replicates). (**d**) qPCR of select NPC and MES gene expression from RNA collected at Day 32 in DM (n=2 independent replicates). Each dot represents the average of four technical replicates. Source data are provided as a Source Data file.

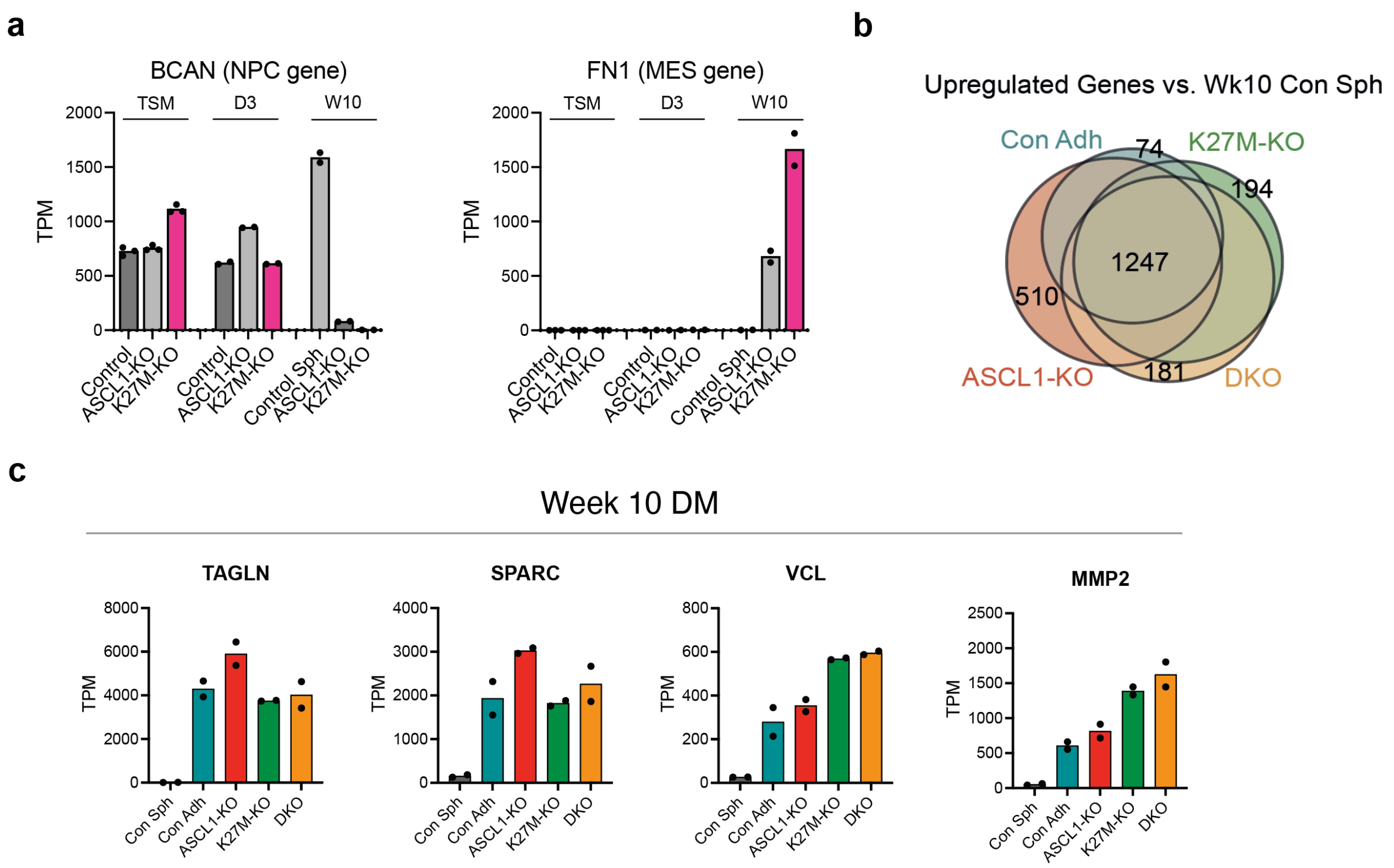

Supplementary Figure 9. A MES-like subpopulation arises without genetic manipulation.

(**a**) TPM (transcripts per million) of BCAN (NPC gene) and FN1 (MES gene) expression in TPM, and Day 3 (D3) and Week 10 (W10) in DM. RNA-seq was performed as n=3 independent biological replicates in TSM and n=2 independent biological replicates in DM. (**b**) Venn diagram showing number and overlap of upregulated genes relative to Wk10 Con Spheres (>2-fold, padj < 0.05), in Wk10 DM Con Adh, ASCL1-KO, K27M-KO, and DKO (n=2 independent biological replicates). (**c**) Expression changes of specific mesenchymal genes (n=2 independent biological replicates). TPM, transcripts per million. Source data are provided as a Source Data file.

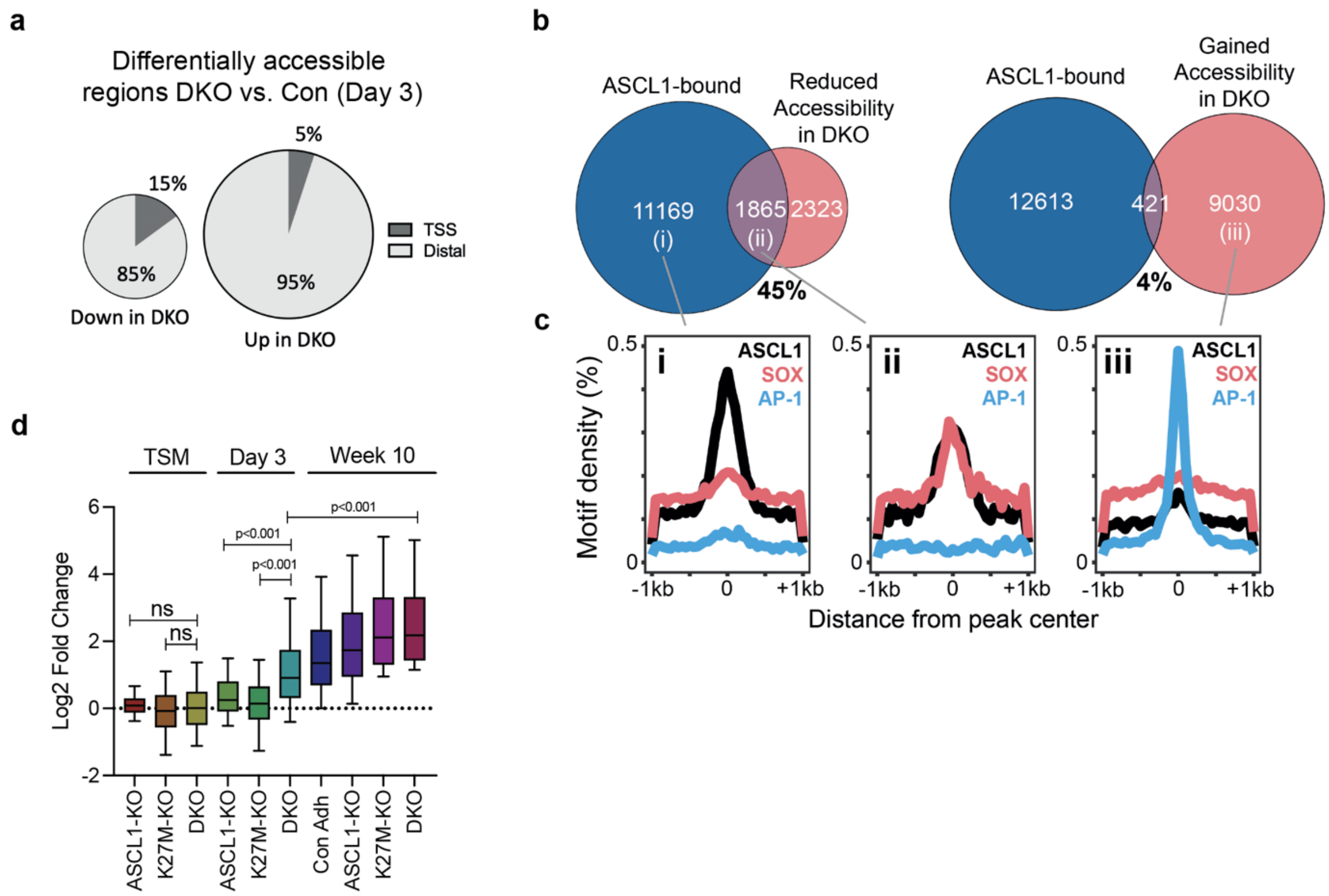

Supplementary Figure 10. ASCL1 and K27M cooperatively regulate chromatin accessibility.

(**a**) Fractions of promoter-proximal (TSS) and distal (>1kb from TSS) differentially accessible regions in DKO vs. Control (Day 3 DM). TSS, transcriptional start site. DKO, Double-knockout of ASCL1 and H3K27M. (**b**) Venn diagrams showing overlap between DKO-specific DARs and the combined set of ASCL1-bound regions in Day 0 (TSM) and Day 3 DM in DIPG-XIII. Numbers indicate peak numbers in each set, while percentages in bold indicate the percent of differential CUTAC peaks overlapping ASCL1 peaks. (**c**) Density plots showing motif density of the corresponding TFs at peak sets (i), (ii), and (iii) shown above. Subset (ii) shows selective co-enrichment of SOX and ASCL1 motifs. (**d**) Expression changes of the n=732 genes associated with increased accessibility and BRG1 binding in DIPG-XIII Day3 DKO vs. Control, which are also upregulated (log2FoldChange >1 & padj < 0.05) in DKO vs. Control gliomaspheres at Week 10 in DM. Con Adh, adherent subpopulation of control cells. Box: 25th to 75th percentiles, middle line = median; whiskers = 10th to 90th percentile. Statistic: two-tailed t-test. RNA-seq was performed as n=3 independent biological replicates in TSM and n=2 independent biological replicates in DM.

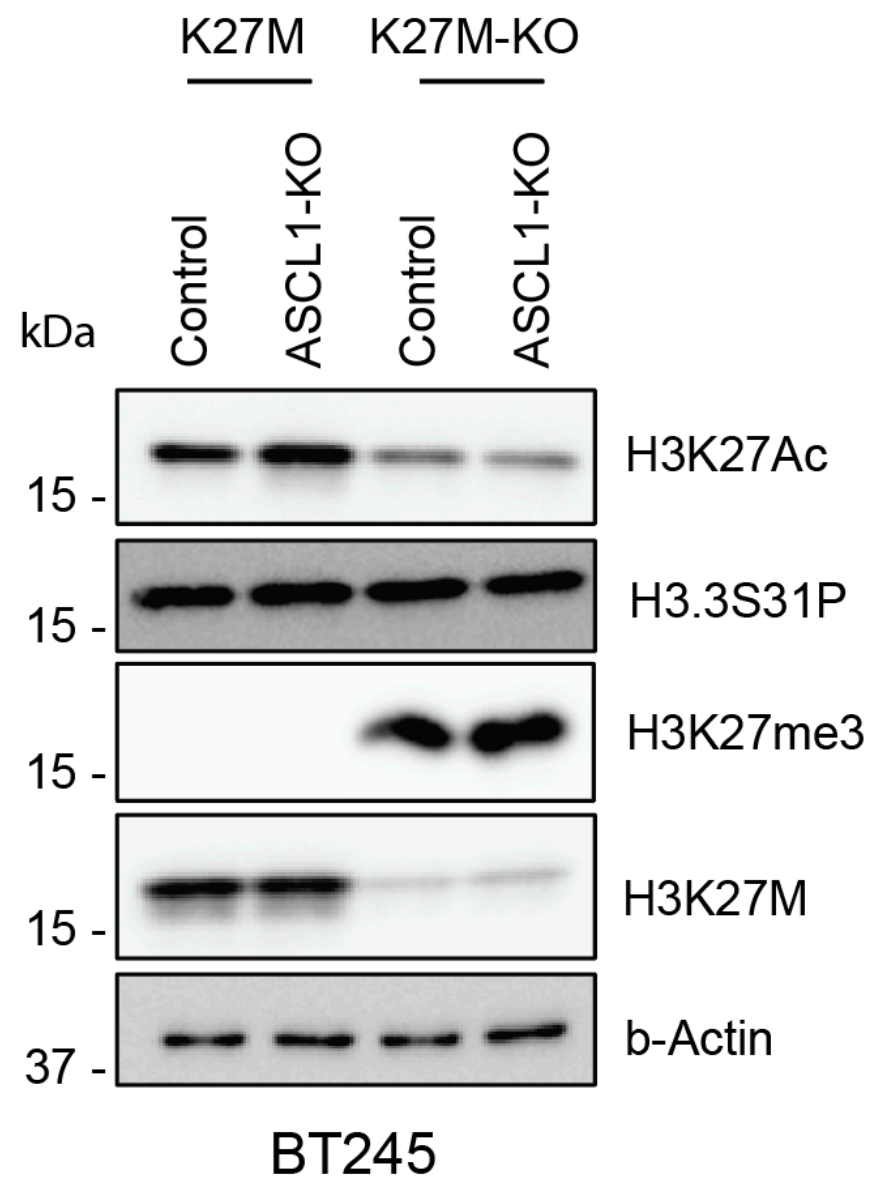

Supplementary Figure 11. **K27M-KO restores H3K27me3 and reduces H3K27Ac in BT245.**

Western blot for indicated histone post-translational modifications in K27M-KO and ASCL1-KO BT245 cells. This experiment was performed once. Source data are provided as a Source Data file.

Supplementary References

1. Li, W. *et al.* Quality control, modeling, and visualization of CRISPR screens with MAGeCK-VISPR. *Genome Biol.* **16**, 1–13 (2015).
2. Braun, E. *et al.* Comprehensive cell atlas of the first-trimester developing human brain. *Science.* **382**, (2023).
3. Siletti, K. *et al.* Transcriptomic diversity of cell types across the adult human brain. *Science.* **382**, (2023).
