# Supplementary material for: H3K27M cooperates with ASCL1 to maintain progenitor states and drive gliomagenesis
Source: Nat Commun. 2026 Oct 10;17:11510. doi: 10.1038/s41467-026-78138-z (PMC13745522; doi:10.1038/s41467-026-78138-z)
Supplement: Supplementary file 2 [file 41467_2026_78138_MOESM2_ESM.pdf]

Description of Additional Supplementary Files

Supplementary Data 1

Normalized sgRNA counts from CRISPR dropout screen in Control (K27M) and K27M-KO DIPGXIII cells grown in TSM.

Supplementary Data 2

Genes cooperatively regulated by ASCL1 and H3.3K27M in DIPGXIII, at Day 3 post-serum differentiation. A cooperatively regulated gene was defined as one where its adjusted p-value > 0.05 in both single KOs vs. Control, while its abs(Log2FoldChange) > 1 and adjusted p-value < 0.05 in DKO vs Control. p-values from Wald tests were adjusted for multiple comparisons using the Benjamini–Hochberg method.
