# Supplementary material for: H3K27M cooperates with ASCL1 to maintain progenitor states and drive gliomagenesis
Source: Nat Commun. 2026 Oct 10;17:11510. doi: 10.1038/s41467-026-78138-z (PMC13745522; doi:10.1038/s41467-026-78138-z)
Supplement: Supplementary file 3 [file 41467_2026_78138_MOESM5_ESM.pdf]

nature portfolio

Corresponding author(s): Yang Shi, Nada Jabado, Luciano Di Croce

Last updated by author(s): Aug 7, 2026

Reporting Summary

Nature Portfolio wishes to improve the reproducibility of the work that we publish. This form provides structure for consistency and transparency in reporting. For further information on Nature Portfolio policies, see our Editorial Policies and the Editorial Policy Checklist.

Statistics

For all statistical analyses, confirm that the following items are present in the figure legend, table legend, main text, or Methods section.

n/a	Confirmed	
[ ]	[x]	The exact sample size (n) for each experimental group/condition, given as a discrete number and unit of measurement
[ ]	[x]	A statement on whether measurements were taken from distinct samples or whether the same sample was measured repeatedly
[ ]	[x]	The statistical test(s) used AND whether they are one- or two-sided<br>*Only common tests should be described solely by name; describe more complex techniques in the Methods section.*
[ ]	[x]	A description of all covariates tested
[ ]	[x]	A description of any assumptions or corrections, such as tests of normality and adjustment for multiple comparisons
[ ]	[x]	A full description of the statistical parameters including central tendency (e.g. means) or other basic estimates (e.g. regression coefficient) AND variation (e.g. standard deviation) or associated estimates of uncertainty (e.g. confidence intervals)
[ ]	[x]	For null hypothesis testing, the test statistic (e.g. F, t, r) with confidence intervals, effect sizes, degrees of freedom and P value noted<br>*Give P values as exact values whenever suitable.*
[x]	[ ]	For Bayesian analysis, information on the choice of priors and Markov chain Monte Carlo settings
[x]	[ ]	For hierarchical and complex designs, identification of the appropriate level for tests and full reporting of outcomes
[ ]	[x]	Estimates of effect sizes (e.g. Cohen's d, Pearson's r), indicating how they were calculated

Our web collection on statistics for biologists contains articles on many of the points above.

Software and code

Policy information about availability of computer code

Data collection	No software was generated in this study. The Bio-Rad ChemiDoc was used for the acquisition of western blot images. Libraries for RNA-seq, CUT&RUN, and ATAC-seq were sequenced on the Illumina NextSeq 2000. DNA/RNA concentration measurements were performed using the Thermo Fisher NanoDrop Lite (ND1000) and the Qubit. Bioluminescent imaging was collected using the IVIS Spectrum In Vivo Imaging System.
Data analysis	GraphPad Prism 10 was used for analysis of in vivo and in vitro phenotypic assays and for most of graph production and for the statistical test.<br>Statistical analysis of CRISPR screen results was conducted via the MAGeCK (version 0.5.9.5) package.<br>For RNA-seq, fastq files were aligned to the hg38 genome using STAR v2.7.9a. Bam files were sorted and indexed using samtools v1.8. Gene counts were generated using featureCounts from the Rsubread v2.6.1 package, using the inbuilt hg38 genome annotation and default parameters. Differential gene expression was performed with DESeq2 v1.32.0. P-values were adjusted using the Benjamini-Hochberg procedure.<br>For CUT&RUN and CUT&Tag analysis, reads were aligned to the human hg38 (target genome) and Saccharomyces cerevisiae sacCer3 (spike-in genome) using Bowtie2 v2.3.5.1. Spike-in-normalized bigwig track files were created using bamCoverage from deepTools v3.3.1. Peaks were called with MACS2 v2.6.<br>For single-cell RNA+ATAC, Count matrices were generated by mapping reads to hg38 using CellRanger-ARC (v2.0.0) and SAMtools (v1.18). Downstream processing was performed in Seurat v5.<br><br>Statistical analysis of CRISPR screen results was conducted via the MAGeCK (version 0.5.9.5) package.<br>For RNA-seq, fastq files were aligned to the hg38 genome using STAR v2.7.9a. Bam files were sorted and indexed using samtools v1.8. Gene counts were generated using featureCounts from the Rsubread v2.6.1 package, using the inbuilt hg38 genome annotation and default parameters. Differential gene expression was performed with DESeq2 v1.32.0. P-values were adjusted using the Benjamini-Hochberg

procedure.
For CUT&RUN and CUT&Tag analysis, reads were aligned to the human hg38 (target genome) and Saccharomyces cerevisiae sacCer3 (spike-in genome) using Bowtie2 v2.3.5.1. Spike-in-normalized bigwig track files were created using bamCoverage from deepTools v3.3.1. Peaks were called with MACS2 v2.6.

For manuscripts utilizing custom algorithms or software that are central to the research but not yet described in published literature, software must be made available to editors and reviewers. We strongly encourage code deposition in a community repository (e.g. GitHub). See the Nature Portfolio guidelines for submitting code & software for further information.

Data

Policy information about availability of data

All manuscripts must include a data availability statement. This statement should provide the following information, where applicable:

- Accession codes, unique identifiers, or web links for publicly available datasets
- A description of any restrictions on data availability
- For clinical datasets or third party data, please ensure that the statement adheres to our policy

The sequencing data generated in this study have been deposited and are publicly available in the Gene Expression Omnibus (GEO) database under accession codes GSE269554 (https://www.ncbi.nlm.nih.gov/geo/query/acc.cgi?acc=GSE269554) (chromatin accessibility by CUTAC), GSE269555 (https://www.ncbi.nlm.nih.gov/geo/query/acc.cgi?acc=GSE269555) (CUT&RUN), GSE269556 (https://www.ncbi.nlm.nih.gov/geo/query/acc.cgi?acc=GSE269556) (CUT&Tag), GSE269557 (https://www.ncbi.nlm.nih.gov/geo/query/acc.cgi?acc=GSE269557) (bulk RNA-seq), and GSE326427 (https://www.ncbi.nlm.nih.gov/geo/query/acc.cgi?acc=GSE326427) (single-cell RNA+ATAC-seq). The publicly available data used in this study are available in the European Genome-phenome Archive (EGA) under accession code EGAS00001004301, EGAS00001007510, EGAS00001003572, EGAS00001002696, EGAS00001004107; under dbGaP accession number phs002380.v1.p1; on the UCSC cell browser at http://cells.ucsc.edu/?ds=dev-thal, the Gene Expression Omnibus (GEO) under accession code GSE133531, GSE188625, GSE131928, GSE184357, GSE94259; and the BRAIN Initiative Cell Census Network under accession code SCR_015820. The remaining data are available within the Article, Supplementary Information or Source Data file.

Research involving human participants, their data, or biological material

Policy information about studies with human participants or human data. See also policy information about sex, gender (identity/presentation), and sexual orientation and race, ethnicity and racism.

Reporting on sex and gender	Sex/gender was not considered in this study because DMGs occur with similar frequency in both males and females, with similar disease characteristics. This study focused on the molecular mechanisms of tumor-intrinsic cell plasticity.
Reporting on race, ethnicity, or other socially relevant groupings	Race, ethnicity, or other socially relevant groupings were not considered.
Population characteristics	H3K27M-mutant glioma cell lines.
Recruitment	As this was an analysis based on publicly available datasets and cancer cell lines, recruitment was not performed in our study.
Ethics oversight	As this was an analysis based on publicly available datasets and cancer cell lines, ethics oversight was not provided by the Oxford Central University Research Ethics Committee.

Note that full information on the approval of the study protocol must also be provided in the manuscript.

Field-specific reporting

Please select the one below that is the best fit for your research. If you are not sure, read the appropriate sections before making your selection.

[x] Life sciences [ ] Behavioural & social sciences [ ] Ecological, evolutionary & environmental sciences

For a reference copy of the document with all sections, see nature.com/documents/nr-reporting-summary-flat.pdf

Life sciences study design

All studies must disclose on these points even when the disclosure is negative.

Sample size	No statistical methods were used to pre-determine sample sizes.
Data exclusions	No data were excluded from the analysis.
Replication	All attempts at replication were successful.
Randomization	Samples were allocated to groups according to genotype. No randomization was required because the sequencing of nucleic acid libraries are not affected by sample randomization.
Blinding	Data collection and analysis did not involve any blinding procedures. The investigators were not blinded since the treatments were administered by the same party responsible for data collection.

Behavioural & social sciences study design

All studies must disclose on these points even when the disclosure is negative.

Study description	N/A
Research sample	N/A
Sampling strategy	N/A
Data collection	N/A
Timing	N/A
Data exclusions	N/A
Non-participation	N/A
Randomization	N/A

Ecological, evolutionary & environmental sciences study design

All studies must disclose on these points even when the disclosure is negative.

Study description	N/A
Research sample	N/A
Sampling strategy	N/A
Data collection	N/A
Timing and spatial scale	N/A
Data exclusions	N/A
Reproducibility	N/A
Randomization	N/A
Blinding	N/A

Did the study involve field work? ☐ Yes ☒ No

Field work, collection and transport

Field conditions	N/A
Location	N/A
Access & import/export	N/A
Disturbance	N/A

Reporting for specific materials, systems and methods

We require information from authors about some types of materials, experimental systems and methods used in many studies. Here, indicate whether each material, system or method listed is relevant to your study. If you are not sure if a list item applies to your research, read the appropriate section before selecting a response.

Materials & experimental systems

n/a	Involved in the study	
	x	Antibodies
	x	Eukaryotic cell lines
x		Palaeontology and archaeology
	x	Animals and other organisms
x		Clinical data
x		Dual use research of concern
x		Plants

Methods

n/a	Involved in the study	
	x	ChIP-seq
x		Flow cytometry
x		MRI-based neuroimaging

Antibodies

Antibodies used	Western blotting: anti-H3K27me3, Cell Signaling Technology #9733 (1:2000); anti-H3K27M, Abcam ab190631 (1:1000); anti-H3K27me2, Cell Signaling Technology #9728 1:1000); anti-H3K36me2, Abcam ab9049 (1:1000); anti-beta-Actin-HRP, Santa Cruz Biotechnology sc-47778 HRP (1:10000); anti-ASCL1, Santa Cruz Biotechnology sc-374104 (1:1000); anti-histone H4, Cell Signaling Technology #2592 (1:1000); anti-histone H3, Cell Signaling Technology #9715 (1:2000)<br>Immunohistochemistry: anti-histone H3 K27M, RevMab Biosciences, catalogue no. 31-1175-00-S (1:600)<br>Immunofluorescence: anti-Doublecortin (Cell Signaling Technology #4604) (1:1000); anti-beta-tubulin III (GeneTex GT1338) (1:1000)<br>CUT&RUN/CUT&Tag: anti-ASCL1, Abcam ab74065 (1:100); anti-H3K27me3, Cell Signaling Technology #9733 (1:100 for CUT&RUN; 1:75 for CUT&Tag); anti-H3K27M, Abcam ab190631 (1:100); anti-BRG1, Cell Signaling Technology #49360 (1:100)
Validation	All antibodies have been commercially validated for use in the respective assays. We also validated ASCL1 and H3K27M antibodies in CUT&RUN and WB using knockout cell lines.

Eukaryotic cell lines

Policy information about cell lines and Sex and Gender in Research

Cell line source(s)	HEK293T cells were obtained from ATCC. DIPG-XIII (female) cells were obtained from Dr. Michelle Monje. BT245 (male) cells were obtained from Dr. Keith Ligon. HSJ-019 (female) cells were obtained from Dr. Nada Jabado.
Authentication	Cell lines have been authenticated as unique by distinct DNA methylation, RNA-seq, and chromatin patterns. The identity of cell lines were checked by microsatellite typing (DNA fingerprinting). CRISPR/Cas9 edited clones were verified by Sanger sequencing and Western blotting.
Mycoplasma contamination	The cells were confirmed to be negative for mycoplasma.
Commonly misidentified lines<br>(See ICLAC register)	None used in this study.

Palaeontology and Archaeology

Specimen provenance	N/A
Specimen deposition	N/A
Dating methods	N/A

☐ Tick this box to confirm that the raw and calibrated dates are available in the paper or in Supplementary Information.

Ethics oversight	N/A

Note that full information on the approval of the study protocol must also be provided in the manuscript.

Animals and other research organisms

Policy information about studies involving animals; ARRIVE guidelines recommended for reporting animal research, and Sex and Gender in Research

Laboratory animals	4- to 6-week-old NSG (NOD.Cg-PrkdcSCID Il2rgtm1Wjl/SzJ) mice were purchased from Charles River.
Wild animals	No wild animals were used in this study.

Reporting on sex	Only female mice were used to reduce variability associated with sex-specific differences in behavior and housing, as female mice can be group-housed with reduced aggression and stress compared to males, therefore improving experimental consistency. Sex/gender analysis was not performed because this study focused on the molecular mechanisms of tumor-intrinsic cell plasticity
Field-collected samples	No field-collected samples were used in this study.
Ethics oversight	Mice were housed, bred, and handled according to the procedures outlined by the McGill University Health Centre Animal Care Committee and adhering to the Canadian Council on Animal Care's standard.

Note that full information on the approval of the study protocol must also be provided in the manuscript.

Clinical data

Policy information about clinical studies
All manuscripts should comply with the ICMJE guidelines for publication of clinical research and a completed CONSORT checklist must be included with all submissions.

Clinical trial registration	N/A
Study protocol	N/A
Data collection	N/A
Outcomes	N/A

Dual use research of concern

Policy information about dual use research of concern

Hazards

Could the accidental, deliberate or reckless misuse of agents or technologies generated in the work, or the application of information presented in the manuscript, pose a threat to:

No	Yes	
☒	☐	Public health
☒	☐	National security
☒	☐	Crops and/or livestock
☒	☐	Ecosystems
☒	☐	Any other significant area

Experiments of concern

Does the work involve any of these experiments of concern:

No	Yes	
☒	☐	Demonstrate how to render a vaccine ineffective
☒	☐	Confer resistance to therapeutically useful antibiotics or antiviral agents
☒	☐	Enhance the virulence of a pathogen or render a nonpathogen virulent
☒	☐	Increase transmissibility of a pathogen
☒	☐	Alter the host range of a pathogen
☒	☐	Enable evasion of diagnostic/detection modalities
☒	☐	Enable the weaponization of a biological agent or toxin
☒	☐	Any other potentially harmful combination of experiments and agents

Plants

Seed stocks

N/A

Novel plant genotypes

N/A

Authentication

N/A

ChIP-seq

Data deposition

☒ Confirm that both raw and final processed data have been deposited in a public database such as GEO.

☒ Confirm that you have deposited or provided access to graph files (e.g. BED files) for the called peaks.

Data access links
May remain private before publication.

https://www.ncbi.nlm.nih.gov/geo/query/acc.cgi?acc=GSE269554
https://www.ncbi.nlm.nih.gov/geo/query/acc.cgi?acc=GSE269555
https://www.ncbi.nlm.nih.gov/geo/query/acc.cgi?acc=GSE269556
https://www.ncbi.nlm.nih.gov/geo/query/acc.cgi?acc=GSE269557

Files in database submission

CUTAC:
CUTAC_ASCL1KO_Day3_Rep1.R1.fastq.gz
CUTAC_ASCL1KO_Day3_Rep2.R1.fastq.gz
CUTAC_ASCL1KO_TSM_Rep1.R1.fastq.gz
CUTAC_ASCL1KO_TSM_Rep2.R1.fastq.gz
CUTAC_Control_Day3_Rep1.R1.fastq.gz
CUTAC_Control_Day3_Rep2.R1.fastq.gz
CUTAC_Control_TSM_Rep1.R1.fastq.gz
CUTAC_Control_TSM_Rep2.R1.fastq.gz
CUTAC_DKO_Day3_Rep1.R1.fastq.gz
CUTAC_DKO_Day3_Rep2.R1.fastq.gz
CUTAC_DKO_TSM_Rep1.R1.fastq.gz
CUTAC_DKO_TSM_Rep2.R1.fastq.gz
CUTAC_K27MKO_Day3_Rep1.R1.fastq.gz
CUTAC_K27MKO_Day3_Rep2.R1.fastq.gz
CUTAC_K27MKO_TSM_Rep1.R1.fastq.gz
CUTAC_K27MKO_TSM_Rep2.R1.fastq.gz

CUTAC_ASCL1KO_Day3_Rep1.R2.fastq.gz
CUTAC_ASCL1KO_Day3_Rep2.R2.fastq.gz
CUTAC_ASCL1KO_TSM_Rep1.R2.fastq.gz
CUTAC_ASCL1KO_TSM_Rep2.R2.fastq.gz
CUTAC_Control_Day3_Rep1.R2.fastq.gz
CUTAC_Control_Day3_Rep2.R2.fastq.gz
CUTAC_Control_TSM_Rep1.R2.fastq.gz
CUTAC_Control_TSM_Rep2.R2.fastq.gz
CUTAC_DKO_Day3_Rep1.R2.fastq.gz
CUTAC_DKO_Day3_Rep2.R2.fastq.gz
CUTAC_DKO_TSM_Rep1.R2.fastq.gz
CUTAC_DKO_TSM_Rep2.R2.fastq.gz
CUTAC_K27MKO_Day3_Rep1.R2.fastq.gz
CUTAC_K27MKO_Day3_Rep2.R2.fastq.gz
CUTAC_K27MKO_TSM_Rep1.R2.fastq.gz
CUTAC_K27MKO_TSM_Rep2.R2.fastq.gz

CUTAC_ASCL1KO_Day3_Rep1.bamCoverage.bw
CUTAC_ASCL1KO_Day3_Rep2.bamCoverage.bw
CUTAC_ASCL1KO_TSM_Rep1.bamCoverage.bw
CUTAC_ASCL1KO_TSM_Rep2.bamCoverage.bw
CUTAC_Control_Day3_Rep1.bamCoverage.bw
CUTAC_Control_Day3_Rep2.bamCoverage.bw

CUTAC_Control_TSM_Rep1.bamCoverage.bw
CUTAC_Control_TSM_Rep2.bamCoverage.bw
CUTAC_DKO_Day3_Rep1.bamCoverage.bw
CUTAC_DKO_Day3_Rep2.bamCoverage.bw
CUTAC_DKO_TSM_Rep1.bamCoverage.bw
CUTAC_DKO_TSM_Rep2.bamCoverage.bw
CUTAC_K27MKO_Day3_Rep1.bamCoverage.bw
CUTAC_K27MKO_Day3_Rep2.bamCoverage.bw
CUTAC_K27MKO_TSM_Rep1.bamCoverage.bw
CUTAC_K27MKO_TSM_Rep2.bamCoverage.bw

CUT&RUN:

CnR_ASCL1_ASCL1KO_TSM.R1.fastq.gz
CnR_ASCL1_Control_Adh_Wk10_Rep1.R1.fastq.gz
CnR_ASCL1_Control_Adh_Wk10_Rep2.R1.fastq.gz
CnR_ASCL1_Control_Day3_Rep1.R1.fastq.gz
CnR_ASCL1_Control_Day3_Rep2.R1.fastq.gz
CnR_ASCL1_Control_Sph_Wk10_Rep1.R1.fastq.gz
CnR_ASCL1_Control_Sph_Wk10_Rep2.R1.fastq.gz
CnR_ASCL1_Control_TSM_Rep1.R1.fastq.gz
CnR_ASCL1_Control_TSM_Rep2.R1.fastq.gz
CnR_ASCL1_Control_TSM_Rep3.R1.fastq.gz
CnR_ASCL1_K27MKO_TSM_Rep1.R1.fastq.gz
CnR_ASCL1_K27MKO_TSM_Rep2.R1.fastq.gz
CnR_ASCL1_K27MKO_TSM_Rep3.R1.fastq.gz
CnR_ASCL1_K27MKO_Wk10_Rep2.R1.fastq.gz
CnR_BRG1_ASCL1KO_Day3_Rep1.R1.fastq.gz
CnR_BRG1_ASCL1KO_Day3_Rep2.R1.fastq.gz
CnR_BRG1_ASCL1KO_Day3_Rep3.R1.fastq.gz
CnR_BRG1_Control_Day3_Rep1.R1.fastq.gz
CnR_BRG1_Control_Day3_Rep2.R1.fastq.gz
CnR_BRG1_Control_Day3_Rep3.R1.fastq.gz
CnR_BRG1_DKO_Day3_Rep1.R1.fastq.gz
CnR_BRG1_DKO_Day3_Rep2.R1.fastq.gz
CnR_BRG1_DKO_Day3_Rep3.R1.fastq.gz
CnR_BRG1_K27MKO_Day3_Rep1.R1.fastq.gz
CnR_BRG1_K27MKO_Day3_Rep2.R1.fastq.gz
CnR_H3K27M_Control_TSM_rep1.R1.fastq.gz
CnR_H3K27M_Control_TSM_rep2.R1.fastq.gz
CnR_H3K27M_K27MKO_TSM_rep1.R1.fastq.gz
CnR_H3K27M_K27MKO_TSM_rep2.R1.fastq.gz
CnR_H3K27me3_ASCL1KO_Day3_Rep1.R1.fastq.gz
CnR_H3K27me3_ASCL1KO_Wk10_Rep1.R1.fastq.gz
CnR_H3K27me3_Control_Adh_Wk10_Rep1.R1.fastq.gz
CnR_H3K27me3_Control_Day3_Rep1.R1.fastq.gz
CnR_H3K27me3_Control_Sph_Wk10_Rep1.R1.fastq.gz
CnR_H3K27me3_DKO_Day3_Rep1.R1.fastq.gz
CnR_H3K27me3_DKO_Wk10_Rep1.R1.fastq.gz
CnR_H3K27me3_K27MKO_Day3_Rep1.R1.fastq.gz
CnR_H3K27me3_K27MKO_Wk10_Rep1.R1.fastq.gz
CnR_IgG_Control_TSM.R1.fastq.gz

CnR_ASCL1_ASCL1KO_TSM.R2.fastq.gz
CnR_ASCL1_Control_Adh_Wk10_Rep1.R2.fastq.gz
CnR_ASCL1_Control_Adh_Wk10_Rep2.R2.fastq.gz
CnR_ASCL1_Control_Day3_Rep1.R2.fastq.gz
CnR_ASCL1_Control_Day3_Rep2.R2.fastq.gz
CnR_ASCL1_Control_Sph_Wk10_Rep1.R2.fastq.gz
CnR_ASCL1_Control_Sph_Wk10_Rep2.R2.fastq.gz
CnR_ASCL1_Control_TSM_Rep1.R2.fastq.gz
CnR_ASCL1_Control_TSM_Rep2.R2.fastq.gz
CnR_ASCL1_Control_TSM_Rep3.R2.fastq.gz
CnR_ASCL1_K27MKO_TSM_Rep1.R2.fastq.gz
CnR_ASCL1_K27MKO_TSM_Rep2.R2.fastq.gz
CnR_ASCL1_K27MKO_TSM_Rep3.R2.fastq.gz
CnR_ASCL1_K27MKO_Wk10_Rep2.R2.fastq.gz
CnR_BRG1_ASCL1KO_Day3_Rep1.R2.fastq.gz
CnR_BRG1_ASCL1KO_Day3_Rep2.R2.fastq.gz

CnR_BRG1_ASCL1KO_Day3_Rep3.R2.fastq.gz
CnR_BRG1_Control_Day3_Rep1.R2.fastq.gz
CnR_BRG1_Control_Day3_Rep2.R2.fastq.gz
CnR_BRG1_Control_Day3_Rep3.R2.fastq.gz
CnR_BRG1_DKO_Day3_Rep1.R2.fastq.gz
CnR_BRG1_DKO_Day3_Rep2.R2.fastq.gz
CnR_BRG1_DKO_Day3_Rep3.R2.fastq.gz
CnR_BRG1_K27MKO_Day3_Rep1.R2.fastq.gz
CnR_BRG1_K27MKO_Day3_Rep2.R2.fastq.gz
CnR_H3K27M_Control_TSM_rep1.R2.fastq.gz
CnR_H3K27M_Control_TSM_rep2.R2.fastq.gz
CnR_H3K27M_K27MKO_TSM_rep1.R2.fastq.gz
CnR_H3K27M_K27MKO_TSM_rep2.R2.fastq.gz
CnR_H3K27me3_ASCL1KO_Day3_Rep1.R2.fastq.gz
CnR_H3K27me3_ASCL1KO_Wk10_Rep1.R2.fastq.gz
CnR_H3K27me3_Control_Adh_Wk10_Rep1.R2.fastq.gz
CnR_H3K27me3_Control_Day3_Rep1.R2.fastq.gz
CnR_H3K27me3_Control_Sph_Wk10_Rep1.R2.fastq.gz
CnR_H3K27me3_DKO_Day3_Rep1.R2.fastq.gz
CnR_H3K27me3_DKO_Wk10_Rep1.R2.fastq.gz
CnR_H3K27me3_K27MKO_Day3_Rep1.R2.fastq.gz
CnR_H3K27me3_K27MKO_Wk10_Rep1.R2.fastq.gz
CnR_IgG_Control_TSM.R2.fastq.gz

CnR_ASCL1_ASCL1KO_TSM.bamCoverage.spikeNormalized.bw
CnR_ASCL1_Control_Adh_Wk10_Rep1.bamCoverage.spikeNormalized.bw
CnR_ASCL1_Control_Adh_Wk10_Rep2.bamCoverage.spikeNormalized.bw
CnR_ASCL1_Control_Day3_Rep1.bamCoverage.spikeNormalized.bw
CnR_ASCL1_Control_Day3_Rep2.bamCoverage.spikeNormalized.bw
CnR_ASCL1_Control_Sph_Wk10_Rep1.bamCoverage.spikeNormalized.bw
CnR_ASCL1_Control_Sph_Wk10_Rep2.bamCoverage.spikeNormalized.bw
CnR_ASCL1_Control_TSM_Rep1.bamCoverage.spikeNormalized.bw
CnR_ASCL1_Control_TSM_Rep2.bamCoverage.spikeNormalized.bw
CnR_ASCL1_Control_TSM_Rep3.bamCoverage.spikeNormalized.bw
CnR_ASCL1_K27MKO_TSM_Rep1.bamCoverage.spikeNormalized.bw
CnR_ASCL1_K27MKO_TSM_Rep2.bamCoverage.spikeNormalized.bw
CnR_ASCL1_K27MKO_TSM_Rep3.bamCoverage.spikeNormalized.bw
CnR_ASCL1_K27MKO_Wk10_Rep2.bamCoverage.spikeNormalized.bw
CnR_BRG1_ASCL1KO_Day3_Rep1.bamCoverage.spikeNormalized.bw
CnR_BRG1_ASCL1KO_Day3_Rep2.bamCoverage.spikeNormalized.bw
CnR_BRG1_ASCL1KO_Day3_Rep3.bamCoverage.spikeNormalized.bw
CnR_BRG1_Control_Day3_Rep1.bamCoverage.spikeNormalized.bw
CnR_BRG1_Control_Day3_Rep2.bamCoverage.spikeNormalized.bw
CnR_BRG1_Control_Day3_Rep3.bamCoverage.spikeNormalized.bw
CnR_BRG1_DKO_Day3_Rep1.bamCoverage.spikeNormalized.bw
CnR_BRG1_DKO_Day3_Rep2.bamCoverage.spikeNormalized.bw
CnR_BRG1_DKO_Day3_Rep3.bamCoverage.spikeNormalized.bw
CnR_BRG1_K27MKO_Day3_Rep1.bamCoverage.spikeNormalized.bw
CnR_BRG1_K27MKO_Day3_Rep2.bamCoverage.spikeNormalized.bw
CnR_H3K27M_Control_TSM_rep1.bamCoverage.spikeNormalized.bw
CnR_H3K27M_Control_TSM_rep2.bamCoverage.spikeNormalized.bw
CnR_H3K27M_K27MKO_TSM_rep1.bamCoverage.spikeNormalized.bw
CnR_H3K27M_K27MKO_TSM_rep2.bamCoverage.spikeNormalized.bw
CnR_H3K27me3_ASCL1KO_Day3_Rep1.bamCoverage.spikeNormalized.bw
CnR_H3K27me3_ASCL1KO_Wk10_Rep1.bamCoverage.spikeNormalized.bw
CnR_H3K27me3_Control_Adh_Wk10_Rep1.bamCoverage.spikeNormalized.bw
CnR_H3K27me3_Control_Day3_Rep1.bamCoverage.spikeNormalized.bw
CnR_H3K27me3_Control_Sph_Wk10_Rep1.bamCoverage.spikeNormalized.bw
CnR_H3K27me3_DKO_Day3_Rep1.bamCoverage.spikeNormalized.bw
CnR_H3K27me3_DKO_Wk10_Rep1.bamCoverage.spikeNormalized.bw
CnR_H3K27me3_K27MKO_Day3_Rep1.bamCoverage.spikeNormalized.bw
CnR_H3K27me3_K27MKO_Wk10_Rep1.bamCoverage.spikeNormalized.bw
CnR_IgG_Control_TSM.bamCoverage.spikeNormalized.bw

CUT&Tag

CnT_H3K27me3_ASCL1KO_Wk10_Rep1.R1.fastq.gz
CnT_H3K27me3_ASCL1KO_Wk10_Rep2.R1.fastq.gz
CnT_H3K27me3_Control_Adh_Wk10_Rep1.R1.fastq.gz

CnT_H3K27me3_Control_Adh_Wk10_Rep2.R1.fastq.gz
CnT_H3K27me3_Control_Day3_Rep1.R1.fastq.gz
CnT_H3K27me3_Control_Day3_Rep2.R1.fastq.gz
CnT_H3K27me3_Control_Sph_Wk10_Rep1.R1.fastq.gz
CnT_H3K27me3_Control_Sph_Wk10_Rep2.R1.fastq.gz
CnT_H3K27me3_Control_TSM_Rep1.R1.fastq.gz
CnT_H3K27me3_Control_TSM_Rep2.R1.fastq.gz
CnT_H3K27me3_DKO_Wk10_Rep1.R1.fastq.gz
CnT_H3K27me3_DKO_Wk10_Rep2.R1.fastq.gz
CnT_H3K27me3_K27MKO_Day3_Rep1.R1.fastq.gz
CnT_H3K27me3_K27MKO_Day3_Rep2.R1.fastq.gz
CnT_H3K27me3_K27MKO_TSM_Rep1.R1.fastq.gz
CnT_H3K27me3_K27MKO_TSM_Rep2.R1.fastq.gz
CnT_H3K27me3_K27MKO_Wk10_Rep1.R1.fastq.gz
CnT_H3K27me3_K27MKO_Wk10_Rep2.R1.fastq.gz

CnT_H3K27me3_ASCL1KO_Wk10_Rep1.R2.fastq.gz
CnT_H3K27me3_ASCL1KO_Wk10_Rep2.R2.fastq.gz
CnT_H3K27me3_Control_Adh_Wk10_Rep1.R2.fastq.gz
CnT_H3K27me3_Control_Adh_Wk10_Rep2.R2.fastq.gz
CnT_H3K27me3_Control_Day3_Rep1.R2.fastq.gz
CnT_H3K27me3_Control_Day3_Rep2.R2.fastq.gz
CnT_H3K27me3_Control_Sph_Wk10_Rep1.R2.fastq.gz
CnT_H3K27me3_Control_Sph_Wk10_Rep2.R2.fastq.gz
CnT_H3K27me3_Control_TSM_Rep1.R2.fastq.gz
CnT_H3K27me3_Control_TSM_Rep2.R2.fastq.gz
CnT_H3K27me3_DKO_Wk10_Rep1.R2.fastq.gz
CnT_H3K27me3_DKO_Wk10_Rep2.R2.fastq.gz
CnT_H3K27me3_K27MKO_Day3_Rep1.R2.fastq.gz
CnT_H3K27me3_K27MKO_Day3_Rep2.R2.fastq.gz
CnT_H3K27me3_K27MKO_TSM_Rep1.R2.fastq.gz
CnT_H3K27me3_K27MKO_TSM_Rep2.R2.fastq.gz
CnT_H3K27me3_K27MKO_Wk10_Rep1.R2.fastq.gz
CnT_H3K27me3_K27MKO_Wk10_Rep2.R2.fastq.gz

CnT_H3K27me3_ASCL1KO_Wk10_Rep1.bamCoverage.spikeNormalized.bw
CnT_H3K27me3_ASCL1KO_Wk10_Rep2.bamCoverage.spikeNormalized.bw
CnT_H3K27me3_Control_Adh_Wk10_Rep1.bamCoverage.spikeNormalized.bw
CnT_H3K27me3_Control_Adh_Wk10_Rep2.bamCoverage.spikeNormalized.bw
CnT_H3K27me3_Control_Day3_Rep1.bamCoverage.spikeNormalized.bw
CnT_H3K27me3_Control_Day3_Rep2.bamCoverage.spikeNormalized.bw
CnT_H3K27me3_Control_Sph_Wk10_Rep1.bamCoverage.spikeNormalized.bw
CnT_H3K27me3_Control_Sph_Wk10_Rep2.bamCoverage.spikeNormalized.bw
CnT_H3K27me3_Control_TSM_Rep1.bamCoverage.spikeNormalized.bw
CnT_H3K27me3_Control_TSM_Rep2.bamCoverage.spikeNormalized.bw
CnT_H3K27me3_DKO_Wk10_Rep1.bamCoverage.spikeNormalized.bw
CnT_H3K27me3_DKO_Wk10_Rep2.bamCoverage.spikeNormalized.bw
CnT_H3K27me3_K27MKO_Day3_Rep1.bamCoverage.spikeNormalized.bw
CnT_H3K27me3_K27MKO_Day3_Rep2.bamCoverage.spikeNormalized.bw
CnT_H3K27me3_K27MKO_TSM_Rep1.bamCoverage.spikeNormalized.bw
CnT_H3K27me3_K27MKO_TSM_Rep2.bamCoverage.spikeNormalized.bw
CnT_H3K27me3_K27MKO_Wk10_Rep1.bamCoverage.spikeNormalized.bw
CnT_H3K27me3_K27MKO_Wk10_Rep2.bamCoverage.spikeNormalized.bw

Genome browser session
(e.g. UCSC)

N/A. Data viewed in IGV.

Methodology

Replicates

All biological replicates, no technical replicates

Sequencing depth

CUTAC:

CUTAC_ASCL1KO_Day3_Rep1.R1.fastq.gz	18800783
CUTAC_ASCL1KO_Day3_Rep2.R1.fastq.gz	20553412
CUTAC_ASCL1KO_TSM_Rep1.R1.fastq.gz	22330170
CUTAC_ASCL1KO_TSM_Rep2.R1.fastq.gz	16613754
CUTAC_Control_Day3_Rep1.R1.fastq.gz	11026983
CUTAC_Control_Day3_Rep2.R1.fastq.gz	7231161
CUTAC_Control_TSM_Rep1.R1.fastq.gz	9991395
CUTAC_Control_TSM_Rep2.R1.fastq.gz	18109515

CUTAC_DKO_Day3_Rep1.R1.fastq.gz	8643638
CUTAC_DKO_Day3_Rep2.R1.fastq.gz	12252975
CUTAC_DKO_TSM_Rep1.R1.fastq.gz	17561246
CUTAC_DKO_TSM_Rep2.R1.fastq.gz	15105551
CUTAC_K27MKO_Day3_Rep1.R1.fastq.gz	34118182
CUTAC_K27MKO_Day3_Rep2.R1.fastq.gz	15248767
CUTAC_K27MKO_TSM_Rep1.R1.fastq.gz	20207997
CUTAC_K27MKO_TSM_Rep2.R1.fastq.gz	12826628

CUT&RUN:

CnR_ASCL1_ASCL1KO_TSM.R1.fastq.gz	15468192
CnR_ASCL1_Control_Adh_Wk10_Rep1.R1.fastq.gz	15963430
CnR_ASCL1_Control_Adh_Wk10_Rep2.R1.fastq.gz	14962678
CnR_ASCL1_Control_Day3_Rep1.R1.fastq.gz	18034926
CnR_ASCL1_Control_Day3_Rep2.R1.fastq.gz	21519094
CnR_ASCL1_Control_Sph_Wk10_Rep1.R1.fastq.gz	24220069
CnR_ASCL1_Control_Sph_Wk10_Rep2.R1.fastq.gz	20545521
CnR_ASCL1_Control_TSM_Rep1.R1.fastq.gz	16050687
CnR_ASCL1_Control_TSM_Rep2.R1.fastq.gz	92888986
CnR_ASCL1_Control_TSM_Rep3.R1.fastq.gz	22530830
CnR_ASCL1_DKO_Day3.R1.fastq.gz	22942369
CnR_ASCL1_K27MKO_TSM_Rep1.R1.fastq.gz	21395649
CnR_ASCL1_K27MKO_TSM_Rep2.R1.fastq.gz	12186773
CnR_ASCL1_K27MKO_TSM_Rep3.R1.fastq.gz	15445486
CnR_ASCL1_K27MKO_Wk10_Rep2.R1.fastq.gz	20137529
CnR_BRG1_ASCL1KO_Day3_Rep1.R1.fastq.gz	16880338
CnR_BRG1_ASCL1KO_Day3_Rep2.R1.fastq.gz	11020364
CnR_BRG1_ASCL1KO_Day3_Rep3.R1.fastq.gz	22044345
CnR_BRG1_Control_Day3_Rep1.R1.fastq.gz	26599613
CnR_BRG1_Control_Day3_Rep2.R1.fastq.gz	15785562
CnR_BRG1_Control_Day3_Rep3.R1.fastq.gz	29810368
CnR_BRG1_DKO_Day3_Rep1.R1.fastq.gz	7919510
CnR_BRG1_DKO_Day3_Rep2.R1.fastq.gz	7835998
CnR_BRG1_DKO_Day3_Rep3.R1.fastq.gz	13468611
CnR_BRG1_K27MKO_Day3_Rep1.R1.fastq.gz	12452284
CnR_BRG1_K27MKO_Day3_Rep2.R1.fastq.gz	13371144
CnR_H3K27M_Control_TSM_rep1.R1.fastq.gz	5193788
CnR_H3K27M_Control_TSM_rep2.R1.fastq.gz	7149788
CnR_H3K27M_K27MKO_TSM_rep1.R1.fastq.gz	5152249
CnR_H3K27M_K27MKO_TSM_rep2.R1.fastq.gz	5642553
CnR_H3K27me3_ASCL1KO_Day3_Rep1.R1.fastq.gz	14122861
CnR_H3K27me3_ASCL1KO_Wk10_Rep1.R1.fastq.gz	15119256
CnR_H3K27me3_Control_Adh_Wk10_Rep1.R1.fastq.gz	19027825
CnR_H3K27me3_Control_Day3_Rep1.R1.fastq.gz	11760885
CnR_H3K27me3_Control_Sph_Wk10_Rep1.R1.fastq.gz	14093898
CnR_H3K27me3_DKO_Day3_Rep1.R1.fastq.gz	22157729
CnR_H3K27me3_DKO_Wk10_Rep1.R1.fastq.gz	20220186
CnR_H3K27me3_K27MKO_Day3_Rep1.R1.fastq.gz	20044189
CnR_H3K27me3_K27MKO_Wk10_Rep1.R1.fastq.gz	24012880
CnR_IgG_Control_TSM.R1.fastq.gz	33122166

CUT&Tag:

CnT_H3K27me3_ASCL1KO_Wk10_Rep1.R1.fastq.gz	12541068
CnT_H3K27me3_ASCL1KO_Wk10_Rep2.R1.fastq.gz	15234990
CnT_H3K27me3_Control_Adh_Wk10_Rep1.R1.fastq.gz	14828934
CnT_H3K27me3_Control_Adh_Wk10_Rep2.R1.fastq.gz	11246668
CnT_H3K27me3_Control_Day3_Rep1.R1.fastq.gz	10662606
CnT_H3K27me3_Control_Day3_Rep2.R1.fastq.gz	9147801
CnT_H3K27me3_Control_Sph_Wk10_Rep1.R1.fastq.gz	11343727
CnT_H3K27me3_Control_Sph_Wk10_Rep2.R1.fastq.gz	12009054
CnT_H3K27me3_Control_TSM_Rep1.R1.fastq.gz	19713362
CnT_H3K27me3_Control_TSM_Rep2.R1.fastq.gz	15859932
CnT_H3K27me3_DKO_Wk10_Rep1.R1.fastq.gz	27448592
CnT_H3K27me3_DKO_Wk10_Rep2.R1.fastq.gz	51662404
CnT_H3K27me3_K27MKO_Day3_Rep1.R1.fastq.gz	17607802
CnT_H3K27me3_K27MKO_Day3_Rep2.R1.fastq.gz	18746386
CnT_H3K27me3_K27MKO_TSM_Rep1.R1.fastq.gz	17032814
CnT_H3K27me3_K27MKO_TSM_Rep2.R1.fastq.gz	24788021

	CnT_H3K27me3_K27MKO_Wk10_Rep1.R1.fastq.gz 17479362<br>CnT_H3K27me3_K27MKO_Wk10_Rep2.R1.fastq.gz 27926107
Antibodies	anti-H3K27me3, Cell Signaling Technology #9733 (1:100 for CUT&RUN; 1:75 for CUT&Tag)<br>anti-H3K4me2, Sigma-Aldrich 07-030 (1:75 for CUTAC)<br>anti-ASCL1, Abcam ab74065 (1:100 for CUT&RUN)<br>anti-H3K27M, Abcam ab190631 (1:100 for CUT&RUN)<br>anti-BRG1, Cell Signaling Technology #49360 (1:100 for CUT&RUN)
Peak calling parameters	Peaks were called with MACS2 v2.6 with default parameters
Data quality	Quality of chromatin profiles for each sample was assessed by the percentage of mapped reads, the fraction of reads in peaks, and expected genomic distribution patterns based on previous reports. In addition, the quality of ASCL1 and K27M CUT&RUN profiles was confirmed using knockout lines. The quality of H3K27me3 profiles was further confirmed using K27M and K27M-KO lines, where K27M-KO resulted in expected global gains of H3K27me3.
Software	For RNA-seq, fastq files were aligned to the hg38 genome using STAR v2.7.9a. Bam files were sorted and indexed using samtools v1.8. Gene counts were generated using featureCounts from the Rsubread v2.6.1 package, using the inbuilt hg38 genome annotation and default parameters. Differential gene expression was performed with DESeq2 v1.32.0. P-values were adjusted using the Benjamini-Hochberg procedure.<br>For CUT&RUN and CUT&Tag analysis, reads were aligned to the human hg38 (target genome) and Saccharomyces cerevisiae sacCer3 (spike-in genome) using Bowtie2 v2.3.5.1. Spike-in-normalized bigwig track files were created using bamCoverage from deepTools v3.3.1. Peaks were called with MACS2 v2.6. Peak annotation was performed using the annotatePeaks.pl script from Homer v4.11. Gene set enrichment analysis was performed using msigdbr (v7.5.1) and clusterProfiler (v4.0.5).

Flow Cytometry

Plots

Confirm that:

☐ The axis labels state the marker and fluorochrome used (e.g. CD4-FITC).

☐ The axis scales are clearly visible. Include numbers along axes only for bottom left plot of group (a 'group' is an analysis of identical markers).

☐ All plots are contour plots with outliers or pseudocolor plots.

☐ A numerical value for number of cells or percentage (with statistics) is provided.

Methodology

Sample preparation	N/A
Instrument	N/A
Software	N/A
Cell population abundance	N/A
Gating strategy	N/A

☐ Tick this box to confirm that a figure exemplifying the gating strategy is provided in the Supplementary Information.

Magnetic resonance imaging

Experimental design

Design type	N/A
Design specifications	N/A
Behavioral performance measures	N/A

Acquisition

Imaging type(s)	N/A
Field strength	N/A
Sequence & imaging parameters	N/A
Area of acquisition	N/A

Diffusion MRI ☐ Used ☐ Not used

Preprocessing

Preprocessing software	N/A
Normalization	N/A
Normalization template	N/A
Noise and artifact removal	N/A
Volume censoring	N/A

Statistical modeling & inference

Model type and settings	N/A
Effect(s) tested	N/A

Specify type of analysis: ☐ Whole brain ☐ ROI-based ☐ Both

Statistic type for inference (See Eklund et al. 2016)	N/A
Correction	N/A

Models & analysis

n/a	Involved in the study
☐	☐ Functional and/or effective connectivity
☐	☐ Graph analysis
☐	☐ Multivariate modeling or predictive analysis

Functional and/or effective connectivity	N/A
Graph analysis	N/A
Multivariate modeling and predictive analysis	N/A
