# Supplementary material for: H3K27M cooperates with ASCL1 to maintain progenitor states and drive gliomagenesis
Source: Nat Commun. 2026 Oct 10;17:11510. doi: 10.1038/s41467-026-78138-z (PMC13745522; doi:10.1038/s41467-026-78138-z)
Supplement: Supplementary file 4 [file 41467_2026_78138_MOESM6_ESM.pdf]

nature portfolio

Peer Review File

H3K27M cooperates with ASCL1 to maintain progenitor states and drive gliomagenesis

Corresponding Author: Professor Yang Shi

This file contains all reviewer reports in order by version, followed by all author rebuttals in order by version.

Version 0:

Reviewer comments:

Reviewer #1

(Remarks to the Author)
Review -H3K27M co-opts ASCL1 to maintain progenitor states and drive gliomagenesis

This study investigates the role of ASCL1 in H3K27M-driven diffuse midline gliomas (DMGs), demonstrating that ASCL1 is a key mediator of tumor plasticity and the maintenance of oligodendroglial progenitor states. The authors show that H3K27M prevents Polycomb-mediated silencing of ASCL1, which in turn redistributes BRG1 to regulatory elements enriched for ASCL1 and SOX motifs, sustaining neural progenitor gene expression. Thus ASCL1 plays a role in preserving a stem-like, proliferative pool of radial glial (RGC) and oligodendrocyte progenitor cell (OPC)-like states while preventing differentiation into astrocytic and ependymal lineages in DIPG XIIIs. It achieves this by recruiting the BRG1 chromatin remodeler to neural progenitor regulatory elements, thereby promoting stem and neural progenitor gene expression while repressing mesenchymal differentiation. Additionally, ASCL1 is regulated by PRC2, and its silencing contributes to DMG plasticity, enabling the coexistence of RGC/OPC-like and mesenchymal-like states. They further demonstrate that ASCL1 is critical for K27M-dependent differentiation resistance in DIPG XIIIs, and its loss significantly impairs tumor formation, highlighting its potential as a therapeutic target. The study is well-executed, with clear data and figures. However, it relies too heavily on a single DMG/DIPG cell line, which limits the broader applicability of the findings. Despite this limitation, the study provides valuable insights into ASCL1/ BRG1 regulation by H3K27M and its role in maintaining DMG plasticity.

I recommend this manuscript for publication after the correction of the following weaknesses.

1. Failure to cite earlier work: A study by Lewis et al., Histone H3.3 K27M chromatin functions implicate a network of neurodevelopmental factors including ASCL1 and NEUROD1 in DIPG 2022, is cited in the introduction but not for the correct reason. This is study also made reverent K27M cells and was the first to demonstrate the direct impact of H3.3 K27M on the regulation of ASCL1. This result should be properly cited and discussed in the appropriate context. Only 1 paper is even cited from 2024 -are the authors up to date with work on ASCL1/K27M?

2. Over-reliance on a single cell line: The study's dependence on DIPG XIII is a notable limitation. While the authors perform one experiment with a second DMG cell line (BT245) to validate ASCL1 knockout alone in vivo, the extent to which ASCL1 functions similarly in BT245 remains unclear. There is limited experimental validation of any other findings in this second cell line, raising concerns about reproducibility. More robust validation with additional replicates in an independent cell line would strengthen the findings. Given the heterogeneity of DMGs, this work would have been much stronger and may have reveled additional findings if the experiments used three independent DMG cell lines to ensure that the observed effects are not cell-line-specific. Without broader validation, the conclusions regarding ASCL1 remain tentative. There are now multiple papers citing several H3.3 K27M > WT revertant cell lines these can be obtained or new one created for further validation. Notably, again the studies by Lewis et al. in 2022 utilized two K27M > WT revertant cell lines not one cell line.

3. Characterization of the DIPG XIII cell line: The study heavily relies on DIPG XIII cells, yet their characterization is not well presented. A more thorough genomic characterization, array CGH or karyotype analysis, is necessary, as chromosomal alterations—such as gains of chromosome 1 and losses of other chromosomes (and therefore genes) —are frequently observed in DMG/DIPG. Additionally, H3.3K27M is initially heterozygous, present in only one copy of H3.3 (H3F3A), DIPG cells also retain two additional copies of wild-type H3.3(H3F3B), as well as copies of H3.1 and H3.2. It remains unclear

whether the mutant allele has undergone copy number expansion, which could significantly impact the observed phenotype. Providing this information would strengthen the study's conclusions. As the study focuses on H3 K27me3 and complete loss of this mark is shown in the figures for DIPG XIII; however, in many DMG cells/publications, the loss is incomplete at around 80-90%, and the levels of H3K27me2 seem to vary. Can the authors confirm there is a complete loss of H3 K27me3 in this cell line making it very unusual or is this simply an underexposed WB.

4. Limited consideration of other histone PTMs: The study only considers the on the dominant negative effect of H3. K27M on H3K27me3 but does not account for other histone PTMs affected by H3K27M and H3.3 G34R mutations, such as H3.3 acetylation at K27 (H3 K27Ac) or phosphorylation at S31 (H3.3S31P) these modifications also influence chromatin dynamics and gene expression.

Minor: The poor engraftment of DMG cells in xenografts is a well-documented. Consider using 1 of the several genetic mouse models.

N.B.

1) Lewis NA, Klein RH, Kelly C, Yee J, Knoepfler PS. Histone H3.3 K27M chromatin functions implicate a network of neurodevelopmental factors including ASCL1 and NEUROD1 in DIPG. Epigenetics Chromatin. 2022 May 19;15(1):18. doi: 10.1186/s13072-022-00447-6. PMID: 35590427; PMCID: PMC9121554.
2) Sitbon, D., Boyarchuk, E., Dingli, F. et al. Histone variant H3.3 residue S31 is essential for Xenopus gastrulation regardless of the deposition pathway. Nat Commun 11, 1256 (2020). https://doi.org/10.1038/s41467-020-15084-4
3) Day CA, Grigore F, Hakkim FL, Paul S, Langfald A, Weberg M, Fadness S, Schwab P, Sepaniac L, Stumpff J, Vaughan KT, Daniels DJ, Robinson JP, Hinchcliffe EH. The histone H3.3 K27M mutation suppresses Ser31phosphorylation and mitotic fidelity, which can directly drive gliomagenesis. Curr Biol. 2025 Jan 20;35(2):354-362.e7. doi: 10.1016/j.cub.2024.11.035.

Reviewer #2

(Remarks to the Author)
This paper presents compelling evidence for the critical role of ASCL1 in H3K27M-driven diffuse midline gliomas (DMGs), demonstrating how this transcription factor cooperates with the oncohistone mutation to maintain stemness, block differentiation, and drive tumor formation. The long-term differentiation model developed provides valuable insights into these tumors' mechanisms of phenotypic plasticity.

However, several points need to be addressed :
1) How do the authors explain the major dropout of ASCL1 in CRISPR screening despite the mild effect of ASCL1 silencing on proliferation? Is this related to clonogenicity? This question is particularly relevant for the BT245 line, which was not analyzed in the functional part of the study.
2) Alternatively, does this discrepancy suggest that the tumor microenvironment may play a role in ASCL1-dependent gliomagenesis? Have the authors investigated potential interactions between ASCL1 and microenvironmental factors?
3) The long-term differentiation model shows remarkable phenotypic plasticity in K27M-mutant cells. Single-cell RNA-seq on the DM spheres and adherent populations could be of high interest to better characterize cell state transitions and potential intermediate states.
4) While the authors indicate that "gradual gliomasphere emergence in the months following differentiation for many, albeit not all, pediatric glioma lines tested," the phenotypic analyses would benefit from validation in additional cell lines, particularly BT245, to correlate the proposed mechanism with the differences observed in the mouse xenograft model. The transcriptomic switch induced by ASCL1 loss appears less pronounced in this line (Fig. 1g), and it would be interesting to see what happens in DM conditions.
5) The authors state: "However, to our knowledge such plasticity has not been faithfully captured in a single in vitro model." What about recently published organoid models in which these states seem to exist? Could the authors comment on this?
6) If K27M-KO cells completely lose ASCL1 expression after 10 weeks in DM, how do the authors explain the synergistic effects observed in double KO cells? Are these effects limited to early stages of differentiation, before the complete loss of ASCL1 in K27M-KO cells?
7) Have the authors observed any correlation between ASCL1 expression levels and patient outcomes in human DMG samples? Such clinical correlation would further support the biological significance of their findings.

These questions aim to strengthen an impressive study with significant implications for understanding DMG biology and developing potential therapeutic strategies.

Reviewer #3

(Remarks to the Author)
In this study, the authors aim to demonstrate that the transcription factor ASCL1 is a key mediator of cellular states, plasticity, and tumorigenesis in H3K27M diffuse midline gliomas (DMGs). Furthermore, they proposed that ASCL1 function with H3K27M to redistribute BRG1, a chromatin remodeler, potentially through SOX proteins to bind to DNA motifs important for promoting expression of neural progenitor cell (NPC)/oligodendrocyte precursor cell (OPC) genes at the expense of mesenchymal/differentiation genes.

Although this study utilizes innovative sequencing technology to try to uncover the molecular mechanisms of the plasticity

and stem cell hierarchy of H3K27M DMGs, there are some drawbacks with the design of the experiments and the mostly general and superficial analyses of the data. For one, the use of DIPG-XIII cell line, which is highly sensitive to ASCL1 and K27M loss of function, in a cell cultured differentiation paradigm may not accurately capture the complexity or function of ASCL1 as a mediator downstream of K27M mutations in DMGs in an in vivo context, especially since the findings are not validated in a primary, recurrent, or even orthotopic xenograft DMGs. Secondly, many of the analyses used (normalized enrichment scores, metamodules, GSEA) do not clearly demonstrate how unique cell type-specific signature genes for NPC, OPC, astrocytes, and mesenchymal cells are bound by ASCL1 and BRG1, and how the chromatin accessibility at the loci of these genes under the various ASCL1-KO, K27M-KO, DKO contexts altered their expression to support the conclusion that ASCL1 and BRG1 play critical roles in regulating the cellular states or plasticity of DMGs.

Below are comments of analyses in some the main figures that seem problematic.

Figure 1a-c is confusing to understand and it's not clear how the CRISPR dropout screen identified ASCL1 or other genes as "vulnerability" factors in the K27M or K27M-KO cell lines. Secondly, there should be better description of why is ASCL1 gRNA counts are decreased in K27M but not in K27M-KO?
Figure 1g, should show how proliferating/cell cycle genes and each cell type-specific signature genes are altered compared to control. What about mesenchymal signature genes here too, since mesenchymal differentiation is extensively referred to in later figures? If the conclusion is that K27M-KO and ASCL1-KO results in similar decrease in proliferating markers but differentially altered the cell type markers, then this needs to be showed at the individual gene level (cell cycle genes, astrocyte genes, etc). Furthermore, changes in gene expression, especially cell type markers, should be validated at the protein level.

Figures 3a,b is also confusing as it is not clear how many and what genes are altered.

Figure 3d,e should include cell type-specific signature genes and analyses, for example NPC, OPC, astrocytes, mesenchymal; all cellular states described by Neftel et al 2019.

Figure 4c,f,g need to show differentially expressed genes and examples of cell type-specific genes at the gene loci level for H3K27me3 and ASCL1 C&R peaks.

Figure 4d,e: do all the genes highlighted red in d all belong to the 383 (38%) intersected genes in e? This point is not clear.

Figure 5b,f, genes associated with ASCL1 C&R peaks in (b) should be intersected with genes associated with de-enrichment or enrichment of H3Kme3 (f).

Figure 5g, IGV tracks of ASCL1 C&R peaks should be aligned and included with H3K27me3 for the genes indicated, especially SOX genes and various of the cell-type specific genes. SOX10 is not a NPC marker or specific OPC marker.

Figure 6, why is it that IGV tracks of CUTAC, ASCL1 and BRG1 are not shown together in one panel for control, ASCL1-KO, K27M-KO, and DKO? To support the conclusion that ASCL1 and K27M cooperatively direct BRG1 to maintain RGC/OPC stem states, but BRG1 deposition is re-directed to differentiation genes in cells in DM3, genes that clearly define the stem states (NPC/OPC) needs to be shown demonstrating decreases BRG1 deposition in all the KOs compared to control, whereas differentiation genes (i.e. mesenchymal or astrocyte genes) showed increased deposition of BRG1.
Figure 6 c,e, CUTAC peaks and ASCL1 and BRG1 C&R peaks that are unchanged should also be shown.

Minor comments

- The authors need to clearly demonstrate which genes are used to define stem and non-stem states. It is unclear how some of the genes that were used for the various figures (NCAN, TMOD3, FN1) support their conclusion. For example, FN1 is used as a MES gene but does not seem to be specific to these cell types.

- K27M-mutant and K27M-positive are terms used several times in the text along with K27M-KO, which is confusing. Is K27M-mutant or mutations the same as K27M-positive or the same as K27M-KO?

- There is no direct data to support that ASCL1 function through SOX10 or any other SOX transcription factors to direct BRG1 to stem state genes, which weakens conclusion of study.

Reviewer #4

(Remarks to the Author)
Jiao et al investigate the molecular mechanisms underlying phenotypic plasticity and tumorigenesis in H3K27M DMGs, identifying ASCL1 as a critical downstream effector of H3K27M. Using a long-term differentiation approach, combined with genome-wide CRISPR screening, transcriptomic profiling, and epigenomic analyses, the authors demonstrate that ASCL1 collaborates with H3K27M to maintain RGC/OPC-like states by redirecting BRG1 to regulatory elements enriched with ASCL1 and SOX motifs. Loss of ASCL1 reduces tumor formation in vivo, promotes alternative differentiation pathways, and reveals a dynamic interplay between H3K27M, ASCL1, and PRC2 in driving tumor plasticity. The study proposes a K27M-ASCL1-SOX-BRG1 axis as a therapeutic target, offering significant insights into pediatric glioma biology and epigenetic dysregulation in cancer.

1. Depending on tissue availability from the mouse experiments in Fig. 1, it would be interesting to stain (or perform RNA-seq on) the control and ASCL1-KO tumors in the BT245 model to determine the in vivo impact of ASCL1 loss. This could help address whether a differentiated phenotype is observed in vivo. If possible, including K27M-KO and/or DKO xenografts would provide a more complete comparison and confirm the cooperative effects observed in vitro.

2. Could the authors utilize the scRNA-seq from DMG tumors? Correlating the heterogeneity of ASCL1 expression levels or the identified gene signatures in single cell data could further substantiate the results. Although the authors observe the heterogeneity in the control tumor cells only under differentiation pressure, is it possible that a subset of the control cells' chromatin may be poised for differentiation (even under stem-like conditions)? If so, this could be observed in publicly available scRNA-seq (or scATAC-seq if this were available) data derived from patient samples?

3. How does ASCL1 redistribute BRG1 away from mesenchymal sites; is this perhaps through competitive binding or recruitment mechanisms? Additional experiments could clarify direct or indirect interactions between ASCL1, SOX factors, and BRG1. How does dynamic PRC2 recruitment or eviction occur in the presence of H3K27M? Experiments such PRC2 inhibitor treatments under differentiation conditions could help elucidate this interaction.

4. It would be important to demonstrate causality through rescue experiments with ectopic re-expression of ASCL1 or mutant forms unable to interact with BRG1.

5. The overlap between ASCL1 peaks and SEs (Fig. 4e) is interesting, but the functional relevance of this overlap could be explored in more detail. Are these SEs critical for the expression of RGC/OPC genes? Does ASCL1 binding activate these SEs or only co-localizes with them? ChIP-seq for H3K27Ac in ASCL1-KO cells ccould be a straightforward way to test this.

6. The authors nicely show that ASCL1-KO skews toward astrocytic/ependymal signatures, while K27M-KO promotes oligodendroglial differentiation. Are the underlying transcriptional/epigenetic drivers of this divergence known? How does ASCL1 loss enable alternative differentiation? The data, while impressive, remains somewhat descriptive, and addition of some functional experiments would help solidify the findings. In addition, including a schematic/model at the end could help readers summarize the results.

7. Are control cells sgRNA controls? If not, appropriate experimental controls would be important to include.

Version 1:

Reviewer comments:

Reviewer #1

(Remarks to the Author)

I thank the authors for their response to my initial concerns. The revised manuscript is significantly strengthened by the inclusion of extensive new data, particularly the validation in a second independent diffuse midline glioma cell line (BT245). The authors have successfully addressed most previous limitations. The study now provides a robust mechanistic link between H3K27M, Polycomb-mediated silencing, and ASCL1-driven chromatin remodeling. The evidence supporting ASCL1 as a critical mediator of tumor state diversity and a potential therapeutic target is compelling. The authors have met most of my requirements. This manuscript now provides valuable insights into the molecular drivers of DMG plasticity. I recommend this work for publication.

Summary of Improvements

Citations Integrated Lewis et al. (2022) and 15+ new 2024-2025 references. Resolved
Model Diversity Full epigenomic and functional validation in BT245 line. Resolved
Cell Characterization Provided transcript abundance (68% mutant) and genomic history. Resolved
In Vivo Evidence Added clonal ASCL1-KO xenograft data showing abrogated tumorigenesis. Resolved

Reviewer #2

(Remarks to the Author)
Overall, the authors have provided a robust point-by-point response to all comments. The experimental effort undertaken during the revision is substantial, with characterization of a second independent cell line (BT245), including phenotypic plasticity assays, single-nuclei omics, and new xenograft experiments using validated clonal ASCL1-KO lines; addition of a patient survival analysis; ASCL1 rescue experiments; and adoption of a common reference framework (Neftel et al., 2019) for the definition of glioma cell states.
A few points remain partially unresolved at the experimental level (ASCL1, SOX factors, and BRG1 interactions), but these limitations are explicitly acknowledged by the authors and discussed appropriately. They do not undermine the central conclusions of the manuscript, which are robust and of high interest.
I recommend acceptance of this manuscript.

Reviewer #3

(Remarks to the Author)
The authors have satisfactorily addressed most of the concerns raised in my initial review. The manuscript has been strengthened by the inclusion of additional analyses that clarify the transcriptional programs associated with ASCL1, improved gene-level visualization of cell state–associated markers, and the incorporation of an additional DMG model (BT245) with supporting single-cell and in vivo validation experiments. Together, these additions improve the clarity of the presentation and strengthen the overall conclusions of the study.

One point that may be helpful to acknowledge in the discussion is that the primary experimental systems used in this work rely largely on patient-derived DMG cell lines maintained under in vitro culture conditions. While these models preserve key genetic features of H3K27M DMG and demonstrate tumorigenic capacity in xenograft settings, prolonged culture and differentiation paradigms may not fully capture the cellular heterogeneity or tumor microenvironment present in primary tumors. Notably, it is possible that other neurodevelopmental transcription factors or regulatory pathways could interact with or compensate for ASCL1 in more physiological contexts beyond SOX and BRG1.

Reviewer #4

(Remarks to the Author)
The authors have adequately addressed my comments. Thank you.

Open Access This Peer Review File is licensed under a Creative Commons Attribution 4.0 International License, which permits use, sharing, adaptation, distribution and reproduction in any medium or format, as long as you give appropriate credit to the original author(s) and the source, provide a link to the Creative Commons license, and indicate if changes were made.
In cases where reviewers are anonymous, credit should be given to 'Anonymous Referee' and the source.
The images or other third party material in this Peer Review File are included in the article's Creative Commons license, unless indicated otherwise in a credit line to the material. If material is not included in the article's Creative Commons license and your intended use is not permitted by statutory regulation or exceeds the permitted use, you will need to obtain permission directly from the copyright holder.
To view a copy of this license, visit https://creativecommons.org/licenses/by/4.0/

POINT-BY-POINT RESPONSE LETTER

We sincerely thank all the reviewers for their insightful comments, which have significantly improved our manuscript. We have performed an extensive set of experiments and analyses and have revised the manuscript accordingly to address all major concerns. A detailed point-by-point response is provided below; each response (in blue) is prefaced by "Response".

Reviewer #1

This study investigates the role of ASCL1 in H3K27M-driven diffuse midline gliomas (DMGs), demonstrating that ASCL1 is a key mediator of tumor plasticity and the maintenance of oligodendroglial progenitor states. The authors show that H3K27M prevents Polycomb-mediated silencing of ASCL1, which in turn redistributes BRG1 to regulatory elements enriched for ASCL1 and SOX motifs, sustaining neural progenitor gene expression. Thus ASCL1 plays a role in preserving a stem-like, proliferative pool of radial glial (RGC) and oligodendrocyte progenitor cell (OPC)-like states while preventing differentiation into astrocytic and ependymal lineages in DIPG XIIIs. It achieves this by recruiting the BRG1 chromatin remodeler to neural progenitor regulatory elements, thereby promoting stem and neural progenitor gene expression while repressing mesenchymal differentiation. Additionally, ASCL1 is regulated by PRC2, and its silencing contributes to DMG plasticity, enabling the coexistence of RGC/OPC-like and mesenchymal-like states. They further demonstrate that ASCL1 is critical for K27M-dependent differentiation resistance in DIPG XIIIs, and its loss significantly impairs tumor formation, highlighting its potential as a therapeutic target. The study is well-executed, with clear data and figures. However, it relies too heavily on a single DMG/DIPG cell line, which limits the broader applicability of the findings. Despite this limitation, the study provides valuable insights into ASCL1/ BRG1 regulation by H3K27M and its role in maintaining DMG plasticity.

I recommend this manuscript for publication after the correction of the following weaknesses.

Response: We thank the reviewer for their positive comments.

1. Failure to cite earlier work: A study by Lewis et al., Histone H3.3 K27M chromatin functions implicate a network of neurodevelopmental factors including ASCL1 and NEUROD1 in DIPG 2022, is cited in the introduction but not for the correct reason. This is study also made reverent K27M cells and was the first to demonstrate the direct impact of H3.3 K27M on the regulation of ASCL1. This result should be properly cited and discussed in the appropriate context.

Response: We thank the reviewer for these important suggestions. We have clarified the reference to previous work (Lewis et al., 2022)[1] and have also included the reference to earlier work from the same group (Chen et al., 2020).[2] Lines 122-124 now read: "This aligns with previous findings demonstrating H3K27M directly impacts ASCL1 expression and sensitivity to transient ASCL1 knockdown.[1,2] We also now refer to Lewis et al., 2022 when discussing our ASCL1- and K27M-regulated chromatin accessibility data.

Only 1 paper is even cited from 2024 -are the authors up to date with work on ASCL1/K27M?

Response: We have revised our manuscript with a number of updated references, including:

Day et al., 2025 [3]
Beckman et al., 2025 [4]
Lundie-Brown et al., 2025 [5]
Sarnow et al., 2025 [6]
Hamed et al., 2025 [7]
Tirosh and Suva, 2024 [8]
Mbah et al., 2024 [9]
Mangena et al., 2025 [10]
Nouruzi et al., 2024 [11]
Myers et al., 2024 [12]
Wang et al., 2024 [13]
Jiao et al., 2024 [14]
Bruschi et al., 2024 [15]
Azzarelli et al., 2024 [16]
Sitbon et al., 2020 [17]

2. Over-reliance on a single cell line: The study's dependence on DIPG XIII is a notable limitation. While the authors perform one experiment with a second DMG cell line (BT245) to validate ASCL1 knockout alone in vivo, the extent to which ASCL1 functions similarly in BT245 remains unclear. There is limited experimental validation of any other findings in this second cell line, raising concerns about reproducibility. More robust validation with additional replicates in an independent cell line would strengthen the findings. Given the heterogeneity of DMGs, this work would have been much stronger and may have reveled additional findings if the experiments used three independent DMG cell lines to ensure that the observed effects are not cell-line-specific. Without broader validation, the conclusions regarding ASCL1 remain tentative. There are now multiple papers citing several H3.3 K27M > WT revertant cell lines these can be obtained or new one created for further validation. Notably, again the studies by Lewis et al. in 2022 utilized two K27M > WT revertant cell lines not one cell line.

Response: We thank the reviewer for this critical suggestion. To address this concern, we have now substantially expanded our analyses in a second DMG cell line (BT245) to support our conclusions. First, we demonstrate that serum-induced differentiation in BT245 also reveals phenotypic plasticity, including the re-emergence of gliomaspheres. As observed in DIPG-XIII, ASCL1 is also essential for this phenotype in BT245 cells (**Fig. 7a,c**). Second, we performed single-nucleus RNA-seq and ATAC-seq to characterize transcriptional and epigenomic heterogeneity in ASCL1-WT and ASCL1-KO BT245 cells. Consistent with our findings in DIPG-XIII, ASCL1 knockout reduced cell-state diversity (**Fig. 7d**). ASCL1 promoted NPC-like gene expression while permitting co-existence of NPC and MES-like states, whereas ASCL1-KO cells were largely restricted to MES-like states (**Fig. 7e**). Third, we show that ASCL1 negatively

regulates AP-1 motif accessibility while positively regulating accessibility of its own motif and SOX motifs, particularly in actively cycling cells (**Fig. 7f–h**), further supporting conservation of the regulatory program identified in DIPG-XIII. Finally, we repeated BT245 xenograft experiments using a clonal ASCL1-KO line. ASCL1 loss abrogated tumorigenesis, recapitulating the phenotype observed in DIPG-XIII (**Fig. 7i**). In addition to the detailed analyses of BT245 cells described above, we also began to investigate additional DMG cell lines and found similar plasticity regulation and a possible role for ASCL1 (data not shown). Together, these additional functional, epigenomic, and *in vivo* data in an independent DMG model substantially strengthen our conclusion that ASCL1 is a critical regulator of H3K27M-mutant DMG plasticity and tumorigenicity.

3. Characterization of the DIPG XIII cell line: The study heavily relies on DIPG XIII cells, yet their characterization is not well presented. A more thorough genomic characterization, array CGH or karyotype analysis, is necessary, as chromosomal alterations—such as gains of chromosome 1 and losses of other chromosomes (and therefore genes) —are frequently observed in DMG/DIPG. Additionally, H3.3K27M is initially heterozygous, present in only one copy of H3.3 (H3F3A), DIPG cells also retain two additional copies of wild-type H3.3(H3F3B), as well as copies of H3.1 and H3.2. It remains unclear whether the mutant allele has undergone copy number expansion, which could significantly impact the observed phenotype. Providing this information would strengthen the study’s conclusions.

Response: We thank the reviewer for this comment. We examined the relative abundance of mutant and wild-type H3F3A transcripts in our bulk RNA-seq data. Approximately 68% of H3F3A transcripts harbor the K27M mutation (AAG→ATG) (**Reviewer Only Fig. 1**). This suggests that the mutant allele has not undergone significant copy number expansion, consistent with whole exome sequencing of DIPG-XIII performed by Grasso et al., *Nature Medicine* 2015 (PMID: 26151328). The DIPG-XIII cell line has been used extensively in a large number of DMG studies (PMID: 26151328, 38116029; 36471070; 33207202; 33049227; 31526762; 30890717; 38594734), and we now provide a description of the genomic characterization of DIPG-XIII (PMID: 26151328) in the updated text as suggested.

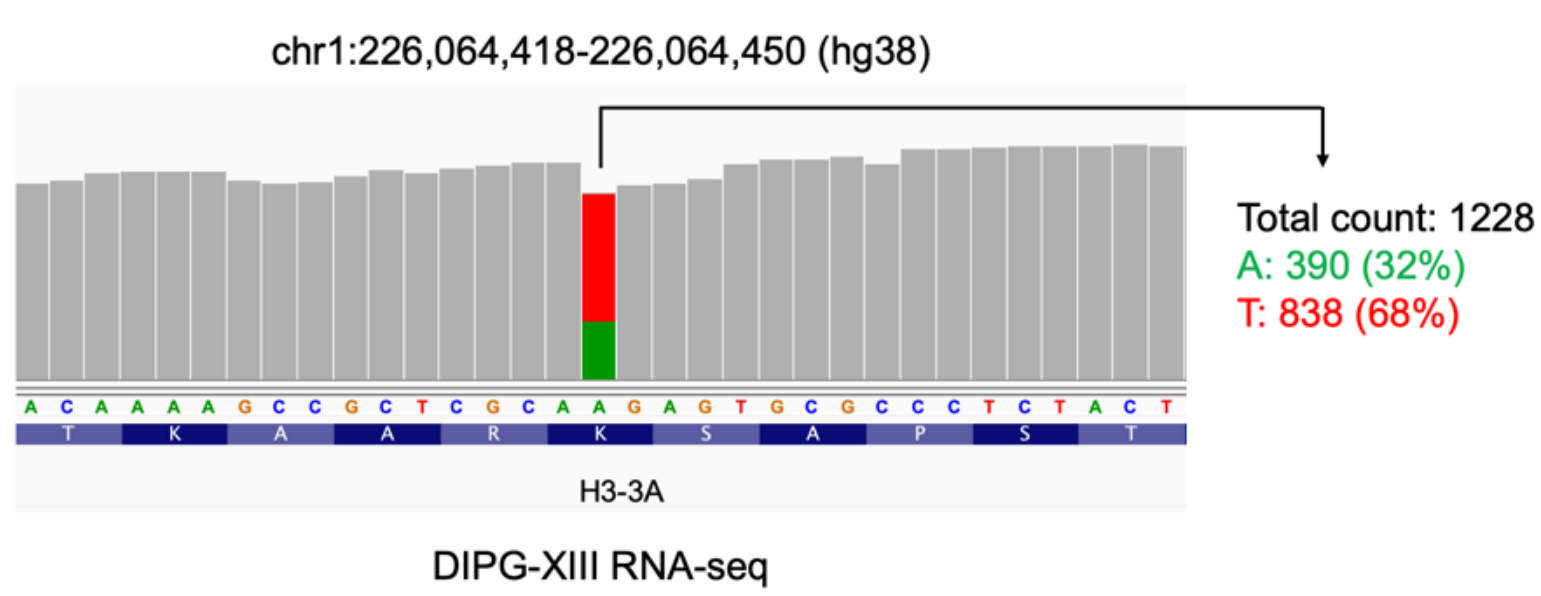

Reviewer Only Fig. 1. RNA-seq read pileup at the H3F3A K27 codon (chr1:226,064,418–226,064,450, hg38) in DIPG-XIII.

As the study focuses on H3 K27me3 and complete loss of this mark is shown in the figures for DIPG XIII; however, in many DMG cells/publications, the loss is incomplete at around 80-90%, and the levels of H3K27me2 seem to vary. Can the authors confirm there is a complete loss of H3 K27me3 in this cell line making it very unusual or is this simply an underexposed WB.

Response: This is an underexposed WB, as also is the case in other publications on the K27M mutation (initial study by Lewis et al, Science 2013, among others for example). As expected, we detect low remaining levels of H3K27me3 in DIPG-XIII, at higher exposure of the H3K27me3 blot (**Extended Data Fig. 1a**). This is consistent with CUT&RUN data (**Reviewer only Fig. 2**), with which we also detect residual H3K27me3 retained at specific loci, in line with previous reports.[18–20] Thus DIPG-XIII is not unusual with respect to H3K27me3 levels, and a more highly exposed WB clarifies this point.

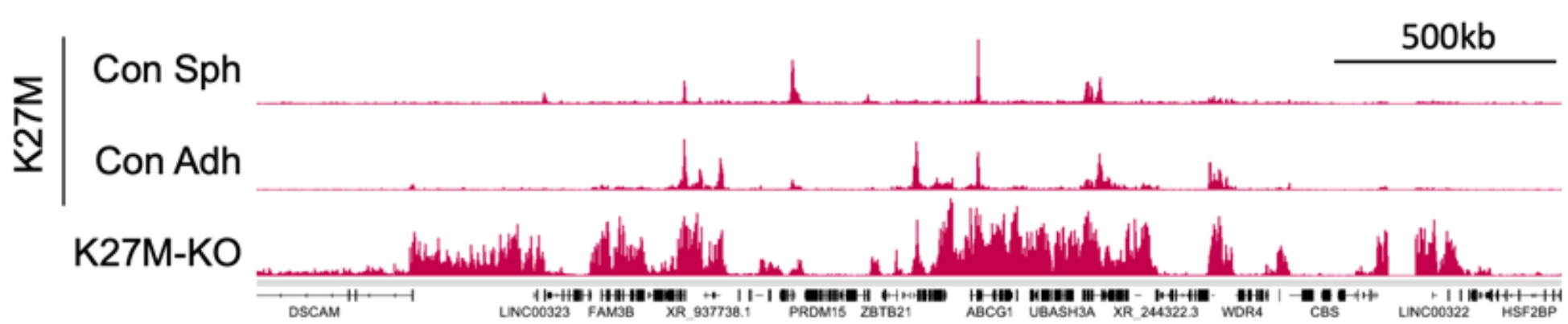

Reviewer Only Fig. 2. IGV tracks of H3K27me3 CUT&RUN in K27M subpopulations (Sph and Adh) and K27M-KO.

4. Limited consideration of other histone PTMs: The study only considers the on the dominant negative effect of H3. K27M on H3K27me3 but does not account for other histone PTMs affected by H3K27M and H3.3 G34R mutations, such as H3.3 acetylation at K27 (H3 K27Ac) or phosphorylation at S31 (H3.3S31P) these modifications also influence chromatin dynamics and gene expression.

Response: We have expanded our consideration of additional histone PTMs. We have added Western blot data of H3K27Ac and histone H3.3 Ser31 phosphorylation (H3.3S31P) levels in H3.3K27M-KO BT245 cells, demonstrating that H3K27Ac is increased by H3K27M as previously shown, while H3.3S31P was largely unchanged (**Extended Data Fig. 11**). Taken together with previous work demonstrating reduced mitotic H3.3S31P driven by H3.3K27M,[3] our data suggest cell cycle stage-specific effects of H3K27M on H3.3S31P. To further investigate additional histone PTMs, we performed CUT&RUN for H3K4me3 following long-term differentiation. We found that ASCL1 was required to maintain H3K4me3 at ASCL1-bound super-enhancers (SEs) (**Fig. 4f**), and loss of ASCL1 strongly reduced the expression of SE-regulated genes (**Fig. 4g**). We hope these additional data address the reviewer's comment.

Minor: The poor engraftment of DMG cells in xenografts is a well-documented. Consider using 1 of the several genetic mouse models.

Response: In the revised manuscript we used two DMG models with robust engraftment in PDOX systems that have been widely used by us and others. The main limitation of these models is the time required for tumor formation, which ranges from 3 to 7 months depending on the model. While genetic mouse models could provide additional insights, they may not reliably reproduce the cell of origin; therefore, we focused on patient-derived cell lines that we and others have validated as faithfully reproducing DMGs. We respectfully think that developing and validating genetic mouse models is beyond the scope of this revision. Instead, to address the reviewer's point, we strengthened the *in vivo* evidence by performing additional xenograft experiments in both DIPG-XIII and BT245, including experiments with clonal ASCL1-KO lines, which further support our conclusion that ASCL1 is required for DMG tumorigenesis (**Fig. 7i**).

N.B.

1) Lewis NA, Klein RH, Kelly C, Yee J, Knoepfler PS. Histone H3.3 K27M chromatin functions implicate a network of neurodevelopmental factors including ASCL1 and NEUROD1 in DIPG. Epigenetics Chromatin. 2022 May 19;15(1):18. doi: 10.1186/s13072-022-00447-6. PMID: 35590427; PMCID: PMC9121554.

2) Sitbon, D., Boyarchuk, E., Dingli, F. et al. Histone variant H3.3 residue S31 is essential for Xenopus gastrulation regardless of the deposition pathway. Nat Commun 11, 1256 (2020). https://doi.org/10.1038/s41467-020-15084-4

3) Day CA, Grigore F, Hakkim FL, Paul S, Langfald A, Weberg M, Fadness S, Schwab P, Sepaniac L, Stumpff J, Vaughan KT, Daniels DJ, Robinson JP, Hinchcliffe EH. The histone H3.3 K27M mutation suppresses Ser31phosphorylation and mitotic fidelity, which can directly drive gliomagenesis. Curr Biol. 2025 Jan 20;35(2):354-362.e7. doi: 10.1016/j.cub.2024.11.035.

Response: We thank the reviewer for highlighting these studies, which we now refer to in our revised manuscript.

Reviewer #2, expertise in diffuse midline gliomas omics and therapy (Remarks to the Author):

This paper presents compelling evidence for the critical role of ASCL1 in H3K27M-driven diffuse midline gliomas (DMGs), demonstrating how this transcription factor cooperates with the oncohistone mutation to maintain stemness, block differentiation, and drive tumor formation. The long-term differentiation model developed provides valuable insights into these tumors' mechanisms of phenotypic plasticity.

Response: We thank the reviewer for their positive comments.

However, several points need to be addressed:

1) How do the authors explain the major dropout of ASCL1 in CRISPR screening despite the mild effect of ASCL1 silencing on proliferation? Is this related to clonogenicity? This question is particularly relevant for the BT245 line, which was not analyzed in the functional part of the study.

Response:

The apparent discrepancy between the strong dropout of ASCL1-targeting gRNAs in the CRISPR screen and the relatively mild effect of ASCL1 silencing on short-term proliferation can be explained by the duration and format of the screen. The screen was conducted over 21 days (approximately 14 cell doublings). Even a modest reduction in proliferation rate (e.g., ~20%) sustained over this period would result in an >80% reduction in final cell numbers, leading to a pronounced dropout phenotype. In addition, clonogenic effects likely contribute. Because the screen is performed in a pooled format, ASCL1–wild-type cells may outcompete ASCL1-knockout cells over time, further amplifying gRNA dropout.

We would like to emphasize that this behavior is similar to what has been observed following K27M removal in DMG models (Harutyunyan et al., *Nat Commun* 2019; Krug et al., *Cancer Cell* 2019; Krug et al., *Nat Genet*, accepted). In both cases, genetic perturbation has limited impact on 2D proliferation assays but leads to profound effects on tumor formation in vivo, largely through promotion of terminal differentiation. ASCL1 loss allows escape from the oligodendroglial lineage blockade via astrocytic/ependymal differentiation, while K27M editing removes the oligodendroglial differentiation blockade itself.

In the BT245 line, ASCL1 knockout did not significantly reduce in vitro proliferation (**Fig. 7a,b**), yet it markedly extended survival in xenograft models. In our initial submission, we used a pooled population of ASCL1 CRISPR/Cas9–edited cells; however, we subsequently determined that this pool contained a substantial fraction of unedited cells. Over time, these unedited cells outcompeted the ASCL1-KO cells and ultimately drove tumor formation.

Consistent with this interpretation, immunostaining revealed that many H3K27M-positive tumor cells retained ASCL1 expression, as shown in the reviewer-only figure below. Tumors generated from injections of pooled ASCL1 CRISPR/Cas9–edited cells were stained by immunohistochemistry (IHC) for H3K27M (yellow) and ASCL1 (teal). Numerous tumor cells co-

stained for H3K27M and ASCL1, appearing green across tumor sections. This staining pattern closely resembled that observed in BT245 unedited tumors, which also displayed both dual ASCL1/H3K27M-positive cells and H3K27M-only cells. These findings recapitulate our in vitro differentiation data, in which ASCL1 expression is maintained in progenitor cells but is downregulated in cells that differentiate along the oligodendroglial lineage, consistent with silencing of this pioneer transcription factor following glial differentiation.

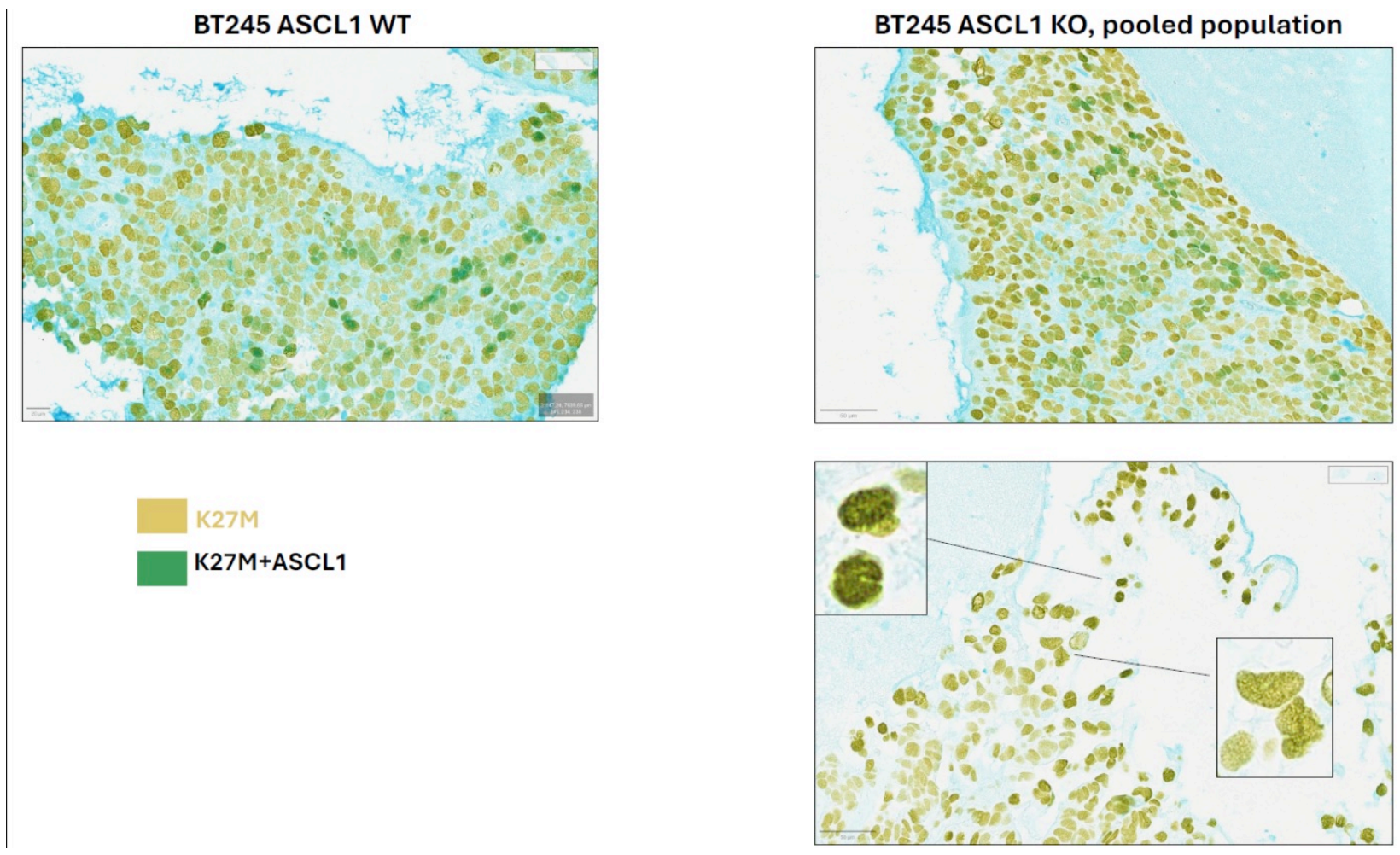

Reviewer Only Fig. 3 Tumors generated from injections of pooled ASCL1 CRISPR/Cas9–edited cells were stained by immunohistochemistry (IHC) for H3K27M (yellow) and ASCL1 (teal). Numerous tumor cells co-stained for H3K27M and ASCL1, appearing green across tumor sections.

When we repeated the BT245 xenograft experiments using a fully clonal ASCL1-KO line, tumorigenesis was completely abrogated (**Fig. 7i**). Thus, although our study was initially motivated by proliferative effects (including potential synthetic lethality with K27M), our data demonstrate that ASCL1 represents a critical in vivo tumor dependency that can be uncoupled from its effects on in vitro proliferation. We have now added substantial functional data to clarify how ASCL1 regulates cellular plasticity in BT245 (described below). Together, these findings support a major role for the in vivo tumor microenvironment in driving ASCL1 dependency, consistent with the reviewer's suggestion.

2) Alternatively, does this discrepancy suggest that the tumor microenvironment may play a role in ASCL1-dependent gliomagenesis? Have the authors investigated potential interactions between ASCL1 and microenvironmental factors?

Response: We thank the reviewer for these insightful questions. We agree with the reviewer's suggestion that ASCL1 functionally interacts with microenvironmental factors. In our manuscript, we have investigated this interaction by inducing differentiation *in vitro*. We show that changing culture conditions from TSM to DM alters ASCL1-dependent phenotypes; for example, differentiation medium exacerbates ASCL1-dependent gene expression programs (**Reviewer only Fig. 4**). Because ASCL1 loss mildly affects *in vitro* proliferation (in TSM) but significantly impairs tumor formation *in vivo*, this suggests that differentiation signals present in the tumor microenvironment enhances the DMG dependency on ASCL1.

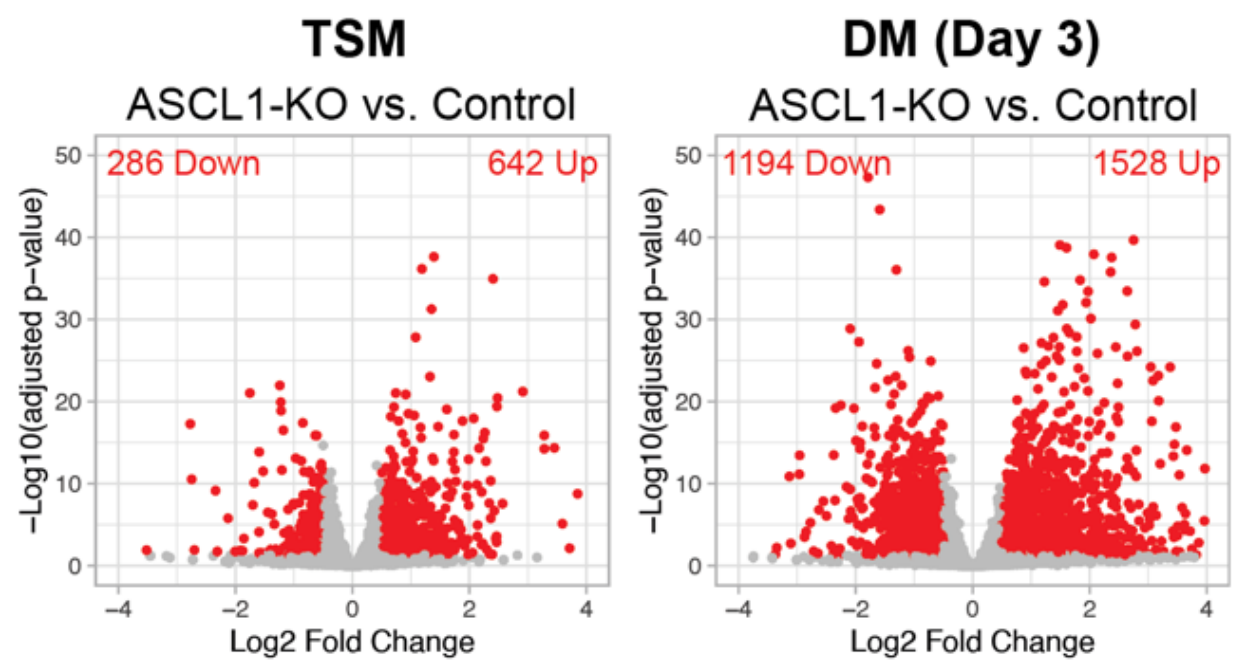

Reviewer only Fig. 4. Volcano plots of differential gene expression in ASCL1-KO vs. Control DIPG-XIII in TSM and at Day 3 in DM. Significantly dysregulated genes (padj < 0.05 and abs(log2 fold change > 0.5) are highlighted in red.

3) The long-term differentiation model shows remarkable phenotypic plasticity in K27M-mutant cells. Single-cell RNA-seq on the DM spheres and adherent populations could be of high interest to better characterize cell state transitions and potential intermediate states.

Response: We thank the reviewer for this critical suggestion. We have performed single-cell RNA+ATAC-seq on BT245 cells in DM, to better characterize cell state transitions and ASCL1-dependent phenotypic plasticity. We find that ASCL1-KO results in reduced cell state diversity, as evidenced by a reduced number of cell clusters (**Fig. 7d**). We mapped NPC and MES gene signatures onto Control and ASCL1-KO cells and found that – as in DIPG-XIII – ASCL1 promoted NPC-like states and enabled the co-existence of NPC and MES-like states, while ASCL1-KO cells were restricted to MES-like states (**Fig. 7e**). To identify potential intermediate states, we performed RNA velocity analysis. This analysis identified actively cycling cells (Cluster 3) as a highly plastic progenitor-like population from which the majority of other cell states arise (**Fig. 7g**). Notably, this recapitulates findings from scRNA-seq of DMG tumors, where actively cycling tumor cells were also found to be the most progenitor-like, giving rise to various differentiated subpopulations[21]. Finally, we show that key NPC- and MES-associated genes and regulatory motifs are ASCL1-dependent in BT245 cells, consistent with our findings in DIPG-XIII (**Fig. 7f**, **h**).

4) While the authors indicate that "gradual gliomasphere emergence in the months following differentiation for many, albeit not all, pediatric glioma lines tested," the phenotypic analyses would benefit from validation in additional cell lines, particularly BT245, to correlate the proposed mechanism with the differences observed in the mouse xenograft model. The transcriptomic switch induced by ASCL1 loss appears less pronounced in this line (Fig. 1g), and it would be interesting to see what happens in DM conditions.

Response: We thank the reviewer for this important suggestion. We now show that like in DIPG-XIII, ASCL1 is required for gliomasphere re-emergence in BT245 under differentiating conditions (**Fig. 7a,c**). Our sc-RNA-seq at Day 6 in DM support that ASCL1-dependent transcriptomic changes are enhanced in DM, including dramatic changes to NPC and MES genes (**Fig. 7e,f**).

5) The authors state: "However, to our knowledge such plasticity has not been faithfully captured in a single in vitro model." What about recently published organoid models in which these states seem to exist? Could the authors comment on this?

Response: We thank the reviewer for the excellent suggestion. We have removed this sentence from the manuscript, and we now comment on recently published organoid models as suggested. The revised manuscript now reads: “While recent advances in brain tumor organoids offer unique opportunities to study cancer cell states, we find that simple monocultures under homogenous differentiating conditions are also sufficient to reveal phenotypic plasticity and its regulators.”

6) If K27M-KO cells completely lose ASCL1 expression after 10 weeks in DM, how do the authors explain the synergistic effects observed in double KO cells? Are these effects limited to early stages of differentiation, before the complete loss of ASCL1 in K27M-KO cells?

Response: Yes, this is exactly our interpretation – the synergy between ASCL1-KO and K27M-KO is limited to early differentiation. The double-KO *accelerates* differentiation, but the end state is similar to single KOs, consistent with the complete loss of ASCL1 in K27M-KO cells.

7) Have the authors observed any correlation between ASCL1 expression levels and patient outcomes in human DMG samples? Such clinical correlation would further support the biological significance of their findings.

Response: We thank the reviewer for this suggestion and have incorporated such an analysis into the manuscript: To maximize statistical power, we pooled all available cases of DMG with H3K27M mutations in either H3F3A, HIST1H3C, HIST1H3D, HIST1H3F from pedcBioportal. This required reclassification of some samples according to the latest WHO classification of CNS tumors of 2021 since many of the datasets were annotated prior to release of this WHO classification of CNS tumors of 2016 that first recognized DMGs with H3K27M mutation as a separate glioma entity. Altogether, we assembled 143 patients in this way and performed multi-group univariate LogRank rest that showed a significant inverse correlation between ASCL1 expression and OS (**Extended Data Fig. 2c**).

These questions aim to strengthen an impressive study with significant implications for understanding DMG biology and developing potential therapeutic strategies.

Response: We thank the reviewer for their positive comments, helpful suggestions, and insightful questions.

Reviewer #3, expertise in glioma stem cells, plasticity and ASCL1 (Remarks to the Author):

In this study, the authors aim to demonstrate that the transcription factor ASCL1 is a key mediator of cellular states, plasticity, and tumorigenesis in H3K27M diffuse midline gliomas (DMGs). Furthermore, they proposed that ASCL1 function with H3K27M to redistribute BRG1, a chromatin remodeler, potentially through SOX proteins to bind to DNA motifs important for promoting expression of neural progenitor cell (NPC)/oligodendrocyte precursor cell (OPC) genes at the expense of mesenchymal/differentiation genes.

Although this study utilizes innovative sequencing technology to try to uncover the molecular mechanisms of the plasticity and stem cell hierarchy of H3K27M DMGs, there are some drawbacks with the design of the experiments and the mostly general and superficial analyses of the data. For one, the use of DIPG-XIII cell line, which is highly sensitive to ASCL1 and K27M loss of function, in a cell cultured differentiation paradigm may not accurately capture the complexity or function of ASCL1 as a mediator downstream of K27M mutations in DMGs in an in vivo context, especially since the findings are not validated in a primary, recurrent, or even orthotopic xenograft DMGs. Secondly, many of the analyses used (normalized enrichment scores, metamodules, GSEA) do not clearly demonstrate how unique cell type-specific signature genes for NPC, OPC, astrocytes, and mesenchymal cells are bound by ASCL1 and BRG1, and how the chromatin accessibility at the loci of these genes under the various ASCL1-KO, K27M-KO, DKO contexts altered their expression to support the conclusion that ASCL1 and BRG1 play critical roles in regulating the cellular states or plasticity of DMGs.

Response: We thank the reviewer for these critical comments. We have added an extensive set of experiments to support our conclusions. As suggested, we now (1) focus our analyses on previously established glioma cell state markers from Neftel et al., 2019, and (2) highlight the expression and chromatin state of unique cell type-specific signature genes throughout the revised manuscript. Moreover, we have significantly strengthened our conclusions using a second cell line (BT245), through additional plasticity assays, single-cell multiomics, and additional *in vivo* experiments. Please refer to Fig. 1 k,l; Fig. 3a, d-g; Fig. 4g,h; Fig. 5g; Fig. 6b,d,e; and Fig. 7 in the revised manuscript.

Below are comments of analyses in some the main figures that seem problematic.

Figure 1a-c is confusing to understand and it's not clear how the CRISPR dropout screen identified ASCL1 or other genes as "vulnerability" factors in the K27M or K27M-KO cell lines. Secondly, there should be better description of why is ASCL1 gRNA counts are decreased in K27M but not in K27M-KO?

Response: We thank the reviewer for these questions and clarify the design and analysis of our CRISPR screen. We now include two important quality control measures for our CRISPR screen (**Fig. 1b, 1c**). Confirming the validity of the screen, sgRNAs targeting essential genes were strongly depleted over time, in both K27M and K27M-KO (**Fig. 1b**), while non-targeting controls

remained stable (**Fig. 1c**). The differential depletion of gRNAs in K27M and K27M-KO is represented by the difference in beta score (**Fig. 1d**). Out of all 18,761 genes targeted, ASCL1 gRNAs were the most differentially depleted in K27M mutant cells compared to K27M-KO (**Fig. 1d**), suggesting a K27M-specific proliferative disadvantage in ASCL1-KO cells in a pooled screen setting. We validated the K27M-specific effects of ASCL1 by deleting ASCL1 in either K27M or K27M-KO (**Fig. 1g**) and observed a mild reduction in cell proliferation upon ASCL1-KO only in K27M mutant cells. This is consistent with our screen results, as even mild (eg. ~20%) reductions in proliferation are compounded over the course of the ~14 cell doublings through which the screen was performed, leading to a >80% reduction in the final number of cells.

Figure 1g, should show how proliferating/cell cycle genes and each cell type-specific signature genes are altered compared to control.

Response: We now show expression changes of 3 cell cycle genes (AURKB, MCM7, CDCA5), two oligodendrocyte genes (CLDN11, TUBB4A) and 2 astro-mesenchymal genes (GJA1, S100A10) in Control, ASCL1-KO, K27M-KO, and DKO. The changes in cell cycle gene expression are consistent with the moderate reduction in proliferation following ASCL1-KO in the presence of K27M (**Fig. 1k,l**).

What about mesenchymal signature genes here too, since mesenchymal differentiation is extensively referred to in later figures? If the conclusion is that K27M-KO and ASCL1-KO results in similar decrease in proliferating markers but differentially altered the cell type markers, then this needs to be showed at the individual gene level (cell cycle genes, astrocyte genes, etc).

Response: We thank the reviewer for this question. The reason why mesenchymal signature genes were not shown is because the mesenchymal signature is primarily upregulated by serum-induced differentiation, whereas all samples analyzed in the original Figure 1g were grown in TSM. We have added individual genes as suggested (**Fig. 1k,l**). Further, we have noticed that the mesenchymal signature is most similar to the astrocyte signature, consistent with previous reports[21], and in fact some genes (e.g., S100A10 and GJA1 (**Fig.1l**) are classified as "astrocytic" in one study[21] and "mesenchymal" in another[22]. Given this overlap, we now refer to these genes as "astro-mesenchymal".

Furthermore, changes in gene expression, especially cell type markers, should be validated at the protein level.

Response: We thank the reviewer for this suggestion. In **Fig. 2c** we provide protein-level evidence that only Control DIPG-XIII cells express the neuronal marker Doublecortin following neuronal differentiation. Further, we agree with the reviewer that validating additional ASCL1-dependent gene expression changes would be important to support our conclusions. However, rather than validating protein-level changes in a small number of genes, we believed it was more valuable to validate entire ASCL1-dependent gene signatures in a different DMG line. We therefore performed single-cell RNA-seq in BT245 cells. Consistent with our findings in DIPG-XIII,

we show that ASCL1 is required in BT245 cells to promote NPC gene signatures and repress MES gene signatures (as defined in Neftel 2019) (**Fig. 7e, f**).

Figures 3a,b is also confusing as it is not clear how many and what genes are altered.

Response: We agree and thank the reviewer for highlighting this point. In our revised manuscript, we clarify how many and what genes are altered by adding **Supplemental Table 2**, which shows the complete list of cooperatively regulated genes by ASCL1 and K27M (downregulated, n=382) and (upregulated, n=598) used to generate the original Figures 3a, b. Because our criteria more accurately define cooperatively regulated genes, we now use "cooperative" instead of "synergistic" to include additive effects. We now show expression changes of specific genes, as suggested, highlighting cell cycle and differentiation markers (**Fig. 3a**), and have moved the original Figure 3a to the supplement.

Figure 3d,e should include cell type-specific signature genes and analyses, for example NPC, OPC, astrocytes, mesenchymal; all cellular states described by Neftel et al 2019.
Response: We thank the reviewer for this helpful suggestion. We now show gene signature scores for all cellular states described by Neftel et al., 2019 as suggested (**Fig. 3e**). For consistency, in **Fig. 3d** we also now only show genes from Neftel 2019 signatures for cell cycle, NPC1-like, and MES1-like. The conclusions are consistent with those from our original figures.

Figure 4c,f,g need to show differentially expressed genes and examples of cell type-specific genes at the gene loci level for H3K27me3 and ASCL1 C&R peaks.

Response: We have added these specific examples as suggested. We now show expression changes of NPC (BCAN) and MES (FN1) genes (defined by Neftel et al., 2019) (**Extended Data Fig. 9a**) as specific examples for genes included in our original Figure 4c. In **Fig. 4h**, we now show RNA, H3K27me3 and ASCL1 CUT&RUN at the MYT1 locus, which is an NPC gene as defined in Neftel 2019. We also include a super-enhancer track (Nagaraja et al., 2017) to support our conclusions that ASCL1 directly binds super-enhancers to promote transcriptional activity of cell state-specific genes. Finally, we included H3K4me3 CUT&RUN data to expand our analyses to additional histone marks. Additional specific examples of ASCL1-dependent genes which are also direct ASCL1 targets is shown in **Fig. 4g**.

Figure 4d,e: do all the genes highlighted red in d all belong to the 383 (38%) intersected genes in e? This point is not clear.

Response: Each dot in the original Figure 4d is an ASCL1 peak, and the strongest 15% of peaks are highlighted red. Some of these peaks are labelled with their nearest gene. In the original Figure 4e, we asked: how many of these red peaks overlap with DIPG super-enhancers? We find that 38% of them overlap with previously annotated super-enhancers. In other words, out of the top 15% of ASCL1 peaks, 38% of them reside in super-enhancers. We have clarified this point in the revised manuscript.

Figure 5b,f, genes associated with ASCL1 C&R peaks in (b) should be intersected with genes associated with de-enrichment or enrichment of H3Kme3 (f).

Response: We have performed this analysis as suggested. We found that 3.5% (240/6788) of ASCL1 peaks in DM spheres overlapped with differential H3K27me3 peaks between Control Sph and Adh subpopulations (2.0% and 1.5% overlap with enriched and depleted H3K27me3, respectively).

Figure 5g, IGV tracks of ASCL1 C&R peaks should be aligned and included with H3K27me3 for the genes indicated, especially SOX genes and various of the cell-type specific genes. SOX10 is not a NPC marker or specific OPC marker.

Response: We thank the reviewer for this comment and have added IGV data at various other cell type-specific genes. Our rationale for showing the SOX10 locus was: (1) SOX10 has been shown to drive H3K27M-dependent phenotypes (Pajovic et al., 2020; Mo et al., 2022). (2) SOX10 is highly expressed in OPCs and in DMGs. (3) SOX10 is a master regulator of neural crest fates, which was recently tied to early tumor formation in glioblastoma[7]. **Fig. 5g** now also shows the NPC gene (and a direct ASCL1 target) DLL3, along with FN1 which is a MES gene[22], and COL1A1 which is highly expressed in diverse mesenchymal cells. As suggested, we have included the RNA-seq, ASCL1, H3K27me3 IGV tracks, and now also show the superenhancer annotation.

Figure 6, why is it that IGV tracks of CUTAC, ASCL1 and BRG1 are not shown together in one panel for control, ASCL1-KO, K27M-KO, and DKO? To support the conclusion that ASCL1 and K27M cooperatively direct BRG1 to maintain RGC/OPC stem states, but BRG1 deposition is re-directed to differentiation genes in cells in DM3, genes that clearly define the stem states (NPC/OPC) needs to be shown demonstrating decreases BRG1 deposition in all the KOs compared to control, whereas differentiation genes (i.e. mesenchymal or astrocyte genes) showed increased deposition of BRG1.

Response: We thank the reviewer for this question. We have now shown IGV tracks of CUTAC, ASCL1, and BRG1 together in one panel, at NPC/OPC and MES genes as defined in Neftel et al., 2019, as suggested (**Fig. 6e**). BRG1 deposition decreases in all the KOs compared to control, whereas differentiation (MES) genes show increased BRG1 deposition in DKOs.

Figure 6 c,e, CUTAC peaks and ASCL1 and BRG1 C&R peaks that are unchanged should also be shown.

Response: We now show the unchanged peaks.

Minor comments

- The authors need to clearly demonstrate which genes are used to define stem and non-stem states. It is unclear how some of the genes that were used for the various figures (NCAN, TMOD3, FN1) support their conclusion. For example, FN1 is used as a MES gene but does not seem to be specific to these cell types.

Response: We completely agree that clearly defining our choice of cell state markers would improve our manuscript. Based on this reviewers' suggestions, we now focus our analysis on genes and loci which were used to define glioma cell state-specific signatures in Neftel et al., 2019 in our revised manuscript. This increases the alignment of our analysis to a widely adopted glioma cell-state framework and, importantly, does not change our conclusion that ASCL1 and K27M cooperate to regulate DMG plasticity.

- K27M-mutant and K27M-positive are terms used several times in the text along with K27M-KO, which is confusing. Is K27M-mutant or mutations the same as K27M-positive or the same as K27M-KO?

Response: We thank the reviewer for this question. We used K27M-mutant and K27M-positive interchangeably in the original manuscript – which we agree can be confusing. For consistency, we have removed "K27M-positive" from the revised manuscript and only use "K27M-mutant".

- There is no direct data to support that ASCL1 function through SOX10 or any other SOX transcription factors to direct BRG1 to stem state genes, which weakens conclusion of study.

Response: We thank the reviewer for this comment and have addressed this in 2 ways.

First, we have performed single-cell multiomics (RNA+ATAC-seq) in differentiating BT245 cells, which show similar ASCL1-dependent phenotypic plasticity as DIPG-XIII (**Fig. 7c,e**). We have identified a proliferative cluster of cells (Cluster 3) which gives rise to most other cell states based on RNA velocity analysis (**Fig. 7d,g**). Focusing on this cluster, we used chromVAR[23] to comprehensively assess the effect of ASCL1 on SOX transcription factor activity and show that ASCL1 loss reduces the accessibility of most of the 17 SOX motifs in the JASPAR database (including SOX4, which is an NPC gene) (**Fig. 7h**). This strengthens our conclusion that ASCL1 functions with SOX TFs to direct neural progenitor-like states.

Second, we have revised the text to highlight that our data is consistent with previously reported direct interactions between ASCL1 and BRG1 in regulating neural differentiation[24]. Our data is also consistent with the previously reported roles of SOX TFs in directing BRG1 activity in DMGs and provides a mechanistic rationale for the reported benefit of targeting BRG1 in K27M-mutant DMG[25,26].

Reviewer #4, expertise in glioma epigenetics, CUT&Tag and CUT&RUN (Remarks to the Author):

Jiao et al investigate the molecular mechanisms underlying phenotypic plasticity and tumorigenesis in H3K27M DMGs, identifying ASCL1 as a critical downstream effector of H3K27M. Using a long-term differentiation approach, combined with genome-wide CRISPR screening, transcriptomic profiling, and epigenomic analyses, the authors demonstrate that ASCL1 collaborates with H3K27M to maintain RGC/OPC-like states by redirecting BRG1 to regulatory elements enriched with ASCL1 and SOX motifs. Loss of ASCL1 reduces tumor formation in vivo, promotes alternative differentiation pathways, and reveals a dynamic interplay between H3K27M, ASCL1, and PRC2 in driving tumor plasticity. The study proposes a K27M-ASCL1-SOX-BRG1 axis as a therapeutic target, offering significant insights into pediatric glioma biology and epigenetic dysregulation in cancer.

1. Depending on tissue availability from the mouse experiments in Fig. 1, it would be interesting to stain (or perform RNA-seq on) the control and ASCL1-KO tumors in the BT245 model to determine the in vivo impact of ASCL1 loss. This could help address whether a differentiated phenotype is observed in vivo. If possible, including K27M-KO and/or DKO xenografts would provide a more complete comparison and confirm the cooperative effects observed in vitro.

Response: We thank the reviewer for this important suggestion. In our initial submission, we used a pooled population of ASCL1 CRISPR/Cas9–edited cells; however, we subsequently determined that this pool contained a substantial fraction of unedited cells. Over time, these unedited cells outcompeted the ASCL1-KO cells and ultimately drove tumor formation. Consistent with this interpretation, immunostaining revealed that many H3K27M-positive tumor cells retained ASCL1 expression, as shown in **Reviewer Only Figure 3** below.

In our revised manuscript, we show new data obtained from injections of single ASCL1KO clones where we validated complete editing and loss of the protein (**Fig. 7a**). These BT245 ASCL1-KO lines did not generate tumors despite a long follow-up of more than 8 months (**Fig. 7i**), suggesting that complete loss of ASCL1 is incompatible with tumor formation. Notably, this phenocopies the effect of K27M-KO, which also abrogates tumorigenic potential in both DIPG-XIII and BT245 (Harutyunyan et al., 2019). Consequently, because neither ASCL1-KO nor K27M-KO generate tumors *in vivo*, there is no tumor tissue available for additional staining or RNA-seq. We have clarified this in the revised manuscript.

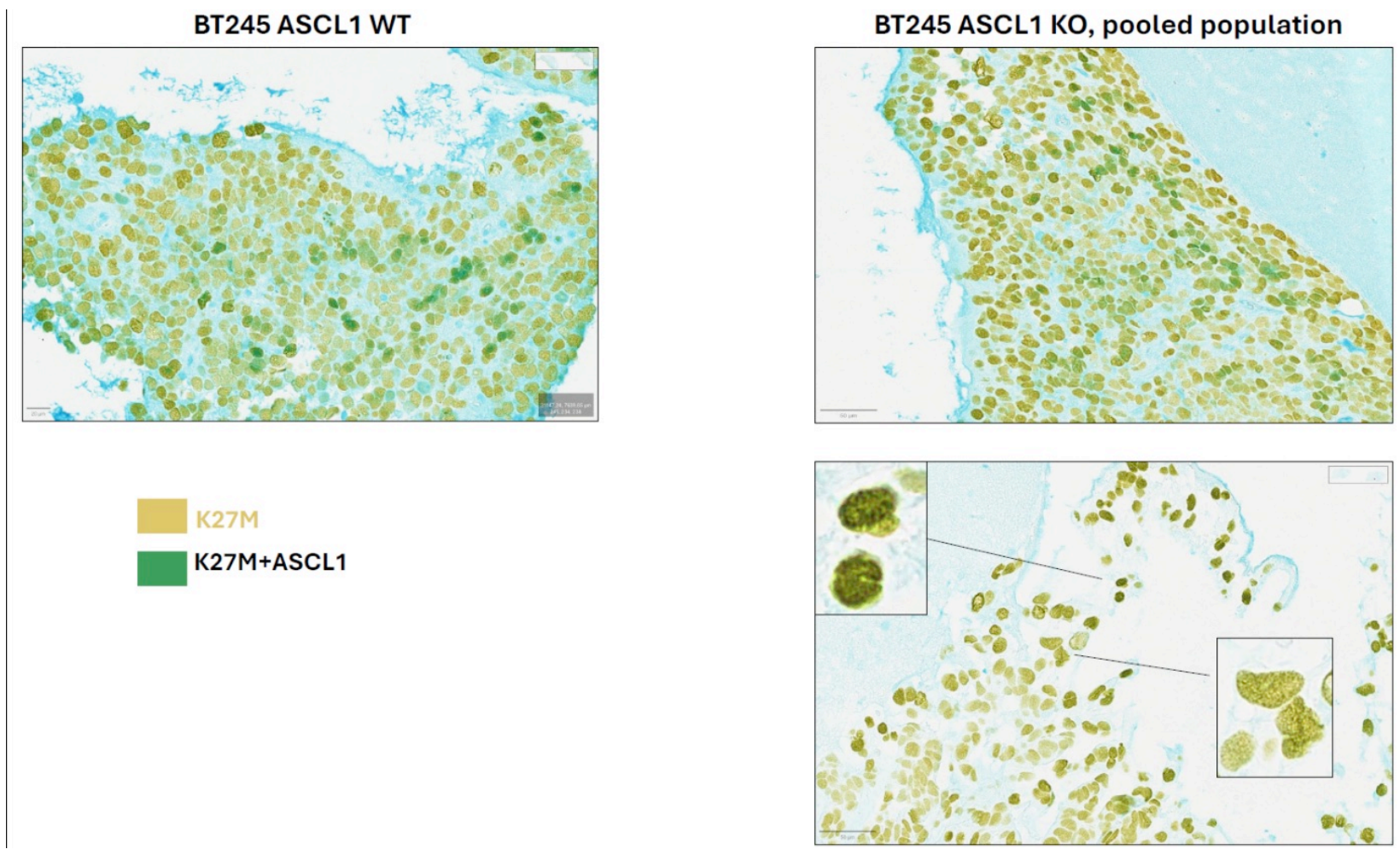

Reviewer Only Fig. 3 Tumors generated from injections of pooled ASCL1 CRISPR/Cas9–edited cells were stained by immunohistochemistry (IHC) for H3K27M (yellow) and ASCL1 (teal). Numerous tumor cells co-stained for H3K27M and ASCL1, appearing green across tumor sections.

2. Could the authors utilize the scRNA-seq from DMG tumors? Correlating the heterogeneity of ASCL1 expression levels or the identified gene signatures in single cell data could further substantiate the results. Although the authors observe the heterogeneity in the control tumor cells only under differentiation pressure, is it possible that a subset of the control cells' chromatin may be poised for differentiation (even under stem-like conditions)? If so, this could be observed in publicly available scRNA-seq (or scATAC-seq if this were available) data derived from patient samples?

Response: We thank the reviewer for this insightful question. To address this, we not only analyzed scRNA-seq from DMG tumors, we also performed single-cell multiomics on differentiating BT245 cells. Using our previously reported scRNA-seq dataset[27], and data from Filbin et al.[21], we show that ASCL1 expression is highest in the stem-like, proliferative DMG subpopulation, and lowest in differentiated subpopulations (**Extended Data Fig. 4** and **Reviewer Only Fig. 5**), supporting our model that heterogeneity in ASCL1 expression drives cell state plasticity in DMGs, with low-ASCL1 cells poised for differentiation *in vivo*.

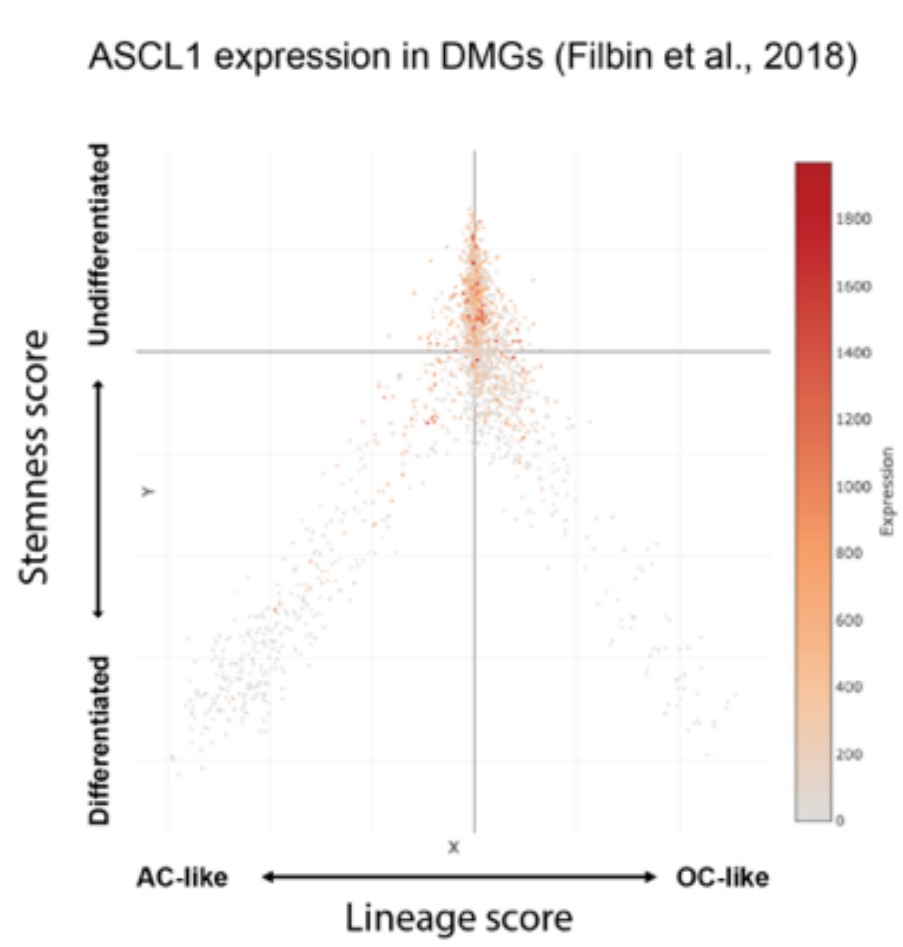

Reviewer Only Fig. 5. Scatter plot from single-cell RNA-sequencing data of pediatric H3K27M diffuse midline gliomas[21]. Each dot represents a single tumor cell, colored by normalized ASCL1 expression level (red gradient). Data visualized using the Broad Institute Single Cell Portal.

To further support this conclusion, we performed single-cell RNA+ATAC-seq of Control and ASCL1-KO BT245 cells under differentiating conditions. To investigate whether a subset of control cells' chromatin may be poised for differentiation, as the reviewer suggests, we show that ASCL1 motif accessibility (and ASCL1 expression) is heterogeneous in control cells (**Fig. 7f**). Low ASCL1 activity correlated with MES-like differentiation (comparing **Fig. 7e** and ASCL1 RNA expression in **7f**). Consistently, ASCL1-KO reduced transcriptional heterogeneity (**Fig. 7e**) and increased the accessibility of FOS transcription factor motifs, members of the AP-1 family known to promote MES-like glioma states (**Fig. 7f**).[28] These data further strengthen our conclusion that heterogeneity in ASCL1 expression and activity enable distinct DMG states.

3. How does ASCL1 redistribute BRG1 away from mesenchymal sites; is this perhaps through competitive binding or recruitment mechanisms? Additional experiments could clarify direct or indirect interactions between ASCL1, SOX factors, and BRG1.

Response: We propose that ASCL1 redistributes BRG1 away from mesenchymal sites via a mechanism recently described for SOX9 [29], where SOX9 opens lineage enhancers while recruiting chromatin modifiers away from alternative lineage enhancers. Our new single-cell RNA+ATAC data from an independent cell line (BT245) shows that, as in DIPG-XIII, ASCL1 is required for maintaining SOX motif accessibility and repressing AP-1 motif accessibility (**Fig. 7f, h).** While we do not directly test physical competition or protein–protein interactions in our study, ASCL1, SOX, and AP-1 family TFs have each been shown independently to interact with and recruit BRG1[29–31]. We have revised the text to place our findings in context of this evidence.

How does dynamic PRC2 recruitment or eviction occur in the presence of H3K27M? Experiments such PRC2 inhibitor treatments under differentiation conditions could help elucidate this interaction.

Response: Because our manuscript focuses on the functional interaction between ASCL1 and H3K27M and how ASCL1 regulates DMG cell state plasticity, we have not investigated how PRC2 recruitment/eviction is affected by H3K27M. However, we agree with the reviewer that this is an important and interesting question, and we have updated our manuscript to discuss previous work investigating how oncohistones impact the dynamic PRC2 recruitment/eviction through live-cell single molecule tracking[32]. This work showed that H3.3K27M stabilizes PRC2 on chromatin but also increases its target search time, resulting in negligible net effects on the level of PRC2 (EZH2) bound to chromatin [32]. This is consistent with independent studies demonstrating negligible[20] or transient[33] effects of H3K27M on PRC2 recruitment.

4. It would be important to demonstrate causality through rescue experiments with ectopic re-expression of ASCL1 or mutant forms unable to interact with BRG1.

Response: We have performed rescue experiments by ectopically expressing 3XFLAG-ASCL1 (**Extended Data Fig. 8a**). We find that re-expressing ASCL1 restores gliomasphere re-emergence (**Extended Data Fig. 8b,c**), albeit at reduced levels compared to wild-type. Incomplete rescue may be due to a combination of (1) reduced 3XFLAG-ASCL1 levels relative to

wild-type, (2) partial loss-of-function due to the 3XFLAG tag, and (3) irreversible effects caused by ASCL1-KO. Nonetheless, we further assessed rescue phenotypes by qPCR, and show that re-introducing ASCL1 can significantly re-activate silenced NPC genes and re-silence activated MES genes (**Extended Data Fig. 8d**). Together these data strengthen our conclusions by showing that ASCL1 is necessary and sufficient for promoting NPC states and repressing MES states in DMG.

To our knowledge, there is no reported ASCL1 mutant that specifically abolishes BRG1 interaction while leaving ASCL1's DNA binding intact. While it has been shown that ASCL1 associates with BRG1 in differentiating neural progenitors[30], a minimal BRG1-contacting motif has not been mapped. We believe mapping such a protein-protein interaction motif is beyond the scope of our manuscript but would be an exciting area for future investigation.

5. The overlap between ASCL1 peaks and SEs (Fig. 4e) is interesting, but the functional relevance of this overlap could be explored in more detail. Are these SEs critical for the expression of RGC/OPC genes? Does ASCL1 binding activate these SEs or only co-localizes with them? ChIP-seq for H3K27Ac in ASCL1-KO cells ccould be a straightforward way to test this.

Response: We thank the reviewer for these critical questions. We have attempted H3K27Ac CUT&RUN experiments in DMGs but have only succeeded in obtaining clear peaks in K27M-KO cells. We suspect this is partially due to increased spreading of H3K27Ac in the presence of K27M, as previously reported.[34,35] Nonetheless, we have successfully performed CUT&RUN for H3K4me3, another active mark found not only at promoters but also at SEs, and show that ASCL1-KO reduces H3K4me3 at super-enhancers (**Fig. 4f**). As the most important test for enhancer functionality, we also show that ASCL1 activates the expression of these SE-associated genes, which include key RGC/OPC genes (**Fig. 4g**). Together with our original Figure 4g (now Fig. 4j), our data strongly support a model where ASCL1 not only binds strongly to SEs but directly activates the expression of SE-associated genes.

6. The authors nicely show that ASCL1-KO skews toward astrocytic/ependymal signatures, while K27M-KO promotes oligodendroglial differentiation. Are the underlying transcriptional/epigenetic drivers of this divergence known?

Response: We favor a model where the persistent expression of ASCL1 in K27M-KO drives the transcriptional divergence between K27M-KO and ASCL1-KO. The differential skewing of cell fates in ASCL1-KO and K27M-KO is reflected by the lack of overlap in genes, which are commonly regulated by ASCL1 and K27M in the absence of differentiation signals. In these conditions, continued ASCL1 expression enables continued repression of astrocytic/ependymal signatures[36], thereby biasing K27M-KO cells toward oligodendroglial fates. Activation of these astrocytic signatures requires ASCL1 to be silenced.

How does ASCL1 loss enable alternative differentiation? The data, while impressive, remains somewhat descriptive, and addition of some functional experiments would help solidify the findings.

Response: We thank the reviewer for this question and comment. We have added considerable functional experiments to solidify our findings that ASCL1 loss drives DMG differentiation. As discussed above in response to comments 2, 3, and 5, our new single-cell analyses in BT245 support our findings from DIPG-XIII, where ASCL1 loss activates the MES-like program, at least in part through increasing AP-1 and reducing SOX family TF motif accessibility. We have clarified a direct, functional role for ASCL1 binding to SEs, which drives NPC/OPC gene expression. Its loss reduces accessibility at ASCL1 motifs as well as SOX motif-containing elements, particularly in the proliferative subpopulation that depends on ASCL1 to maintain NPC/OPC-like states under differentiating conditions. By characterizing ASCL1 function in an additional DMG model, and through additional *in vivo* and single-cell multiomics experiments, we believe we have significantly strengthened our conclusion that ASCL1 is a master regulator of DMG cell state plasticity.

In addition, including a schematic/model at the end could help readers summarize the results.

Response: We fully agree and have included a summary schematic (**Fig. 8**).

7. Are control cells sgRNA controls? If not, appropriate experimental controls would be important to include.

Response: Yes, control cells all express non-targeting control sgRNAs and we have clarified this in the revised manuscript.

References

1. Lewis, N.A., Klein, R.H., Kelly, C., Yee, J., and Knoepfler, P.S. (2022). Histone H3.3 K27M chromatin functions implicate a network of neurodevelopmental factors including ASCL1 and NEUROD1 in DIPG. Epigenetics Chromatin *15*. https://doi.org/10.1186/s13072-022-00447-6.

2. Chen, K.Y., Bush, K., Klein, R.H., Cervantes, V., Lewis, N., Naqvi, A., Carcaboso, A.M., Lechpammer, M., and Knoepfler, P.S. (2020). Reciprocal H3.3 gene editing identifies K27M and G34R mechanisms in pediatric glioma including NOTCH signaling. Commun. Biol. *3*. https://doi.org/10.1038/s42003-020-1076-0.

3. Day, C.A., Grigore, F., Hakkim, F.L., Paul, S., Langfald, A., Weberg, M., Fadness, S., Schwab, P., Sepaniac, L., Stumpff, J., et al. (2025). The histone H3.3 K27M mutation suppresses Ser31phosphorylation and mitotic fidelity, which can directly drive gliomagenesis. Current Biology *35*, 354-362.e7. https://doi.org/10.1016/j.cub.2024.11.035.

4. Beckman, W.F., Parkinson, L.M., Chaytor, L., and Philpott, A. (2025). Distinct proliferative and neuronal programmes of chromatin binding and gene activation by ASCL1 are cell cycle stage-specific. Development *152*. https://doi.org/10.1242/dev.204816.

5. Lundie-Brown, J., Puletti, F., Philpott, A., and Azzarelli, R. (2025). Cell fate acquisition and reprogramming by the proneural transcription factor ASCL1. Preprint at Royal Society Publishing, https://doi.org/10.1098/rsob.250018 https://doi.org/10.1098/rsob.250018.

6. Sarnow, K., Majercak, E., Qurbonov, Q., Cruzeiro, G.A.V., Jeong, D., Haque, I.A., Khalil, A., Baird, L.C., Filbin, M.G., and Tang, X. (2025). Neuroimmune-competent human brain organoid model of diffuse midline glioma. Neuro. Oncol. *27*, 369–382. https://doi.org/10.1093/neuonc/noae245.

7. Hamed, A.A., Hua, K., Trinh, Q.M., Simons, B.D., Marioni, J.C., Stein, L.D., and Dirks, P.B. (2025). Gliomagenesis mimics an injury response orchestrated by neural crest-like cells. Nature *638*, 499–509. https://doi.org/10.1038/s41586-024-08356-2.

8. Tirosh, I., and Suva, M.L. (2024). Cancer cell states: Lessons from ten years of single-cell RNA-sequencing of human tumors. Cancer Cell *42*, 1497–1506. https://doi.org/10.1016/j.ccell.2024.08.005.

9. Mbah, N.E., Myers, A.L., Sajjakulnukit, P., Chung, C., Thompson, J.K., Hong, H.S., Giza, H., Dang, D., Nwosu, Z.C., Shan, M., et al. (2024). Therapeutic targeting of differentiation-state dependent metabolic vulnerabilities in diffuse

midline glioma. Nature Communications *15*. https://doi.org/10.1038/s41467-024-52973-4.

10. Mangena, V., Chanoch-Myers, R., Sartore, R., Paulsen, B., Gritsch, S., Weisman, H., Hara, T., Breakefield, X.O., Breyne, K., Regev, A., et al. (2024). Glioblastoma-cortical organoids recapitulate cell state heterogeneity and intercellular transfer. Cancer Discov. https://doi.org/10.1158/2159-8290.CD-23-1336.

11. Nouruzi, S., Namekawa, T., Tabrizian, N., Kobelev, M., Sivak, O., Scurll, J.M., Cui, C.J., Ganguli, D., and Zoubeidi, A. (2024). ASCL1 regulates and cooperates with FOXA2 to drive terminal neuroendocrine phenotype in prostate cancer. JCI Insight *9*. https://doi.org/10.1172/jci.insight.185952.

12. Myers, B.L., Brayer, K.J., Paez-Beltran, L.E., Villicana, E., Keith, M.S., Suzuki, H., Newville, J., Anderson, R.H., Lo, Y., Mertz, C.M., et al. (2024). Transcription factors ASCL1 and OLIG2 drive glioblastoma initiation and co-regulate tumor cell types and migration. Nature Communications *15*. https://doi.org/10.1038/s41467-024-54750-9.

13. Wang, C., Sun, M., Shao, C., Schlicker, L., Zhuo, Y., Harim, Y., Peng, T., Tian, W., Stöffler, N., Schneider, M., et al. (2024). A multidimensional atlas of human glioblastoma-like organoids reveals highly coordinated molecular networks and effective drugs. NPJ Precis. Oncol. *8*. https://doi.org/10.1038/s41698-024-00500-5.

14. Jiao, A.L., Sendinc, E., Zee, B.M., Wallner, F., and Shi, Y. (2024). An E2 ubiquitin-conjugating enzyme links diubiquitinated H2B to H3K27M oncohistone function. Proc. Natl. Acad. Sci. U. S. A. *121*. https://doi.org/10.1073/pnas.2416614121.

15. Bruschi, M., Midjek, L., Ajlil, Y., Vairy, S., Lancien, M., Ghermaoui, S., Kergrohen, T., Verreault, M., Idbaih, A., de Biagi, C.A.O., et al. (2024). Diffuse midline glioma invasion and metastasis rely on cell-autonomous signaling. Neuro. Oncol. *26*, 553–568. https://doi.org/10.1093/neuonc/noad161.

16. Azzarelli, R., Gillen, S., Connor, F., Lundie-Brown, J., Puletti, F., Drummond, R., Raffaelli, A., and Philpott, A. (2024). Phospho-regulation of ASCL1-mediated chromatin opening during cellular reprogramming. Development *151*. https://doi.org/10.1242/dev.204329.

17. Sitbon, D., Boyarchuk, E., Dingli, F., Loew, D., and Almouzni, G. (2020). Histone variant H3.3 residue S31 is essential for Xenopus gastrulation regardless of the deposition pathway. Nat. Commun. *11*. https://doi.org/10.1038/s41467-020-15084-4.

18. Piunti, A., Hashizume, R., Morgan, M.A., Bartom, E.T., Horbinski, C.M., Marshall, S.A., Rendleman, E.J., Ma, Q., Takahashi, Y.H., Woodfin, A.R., et al. (2017). Therapeutic targeting of polycomb and BET bromodomain proteins in diffuse intrinsic pontine gliomas. Nat. Med. *23*, 493–500. https://doi.org/10.1038/nm.4296.

19. Harutyunyan, A.S., Chen, H., Lu, T., Horth, C., Nikbakht, H., Krug, B., Russo, C., Bareke, E., Marchione, D.M., Coradin, M., et al. (2020). H3K27M in Gliomas Causes a One-Step Decrease in H3K27 Methylation and Reduced Spreading within the Constraints of H3K36 Methylation. Cell Rep. *33*. https://doi.org/10.1016/j.celrep.2020.108390.

20. Harutyunyan, A.S., Krug, B., Chen, H., Papillon-Cavanagh, S., Zeinieh, M., De Jay, N., Deshmukh, S., Chen, C.C.L., Belle, J., Mikael, L.G., et al. (2019). H3K27M induces defective chromatin spread of PRC2-mediated repressive H3K27me2/me3 and is essential for glioma tumorigenesis. Nat. Commun. *10*, 1262. https://doi.org/10.1038/s41467-019-09140-x.

21. Filbin, M.G., Tirosh, I., Hovestadt, V., Shaw, M.L., Escalante, L.E., Mathewson, N.D., Neftel, C., Frank, N., Pelton, K., Hebert, C.M., et al. (2018). Developmental and oncogenic programs in H3K27M gliomas dissected by single-cell RNA-seq. Science. *360*, 331–335. https://doi.org/10.1126/science.aao4750.

22. Neftel, C., Laffy, J., Filbin, M.G., Hara, T., Shore, M.E., Rahme, G.J., Richman, A.R., Silverbush, D., Shaw, M.L., Hebert, C.M., et al. (2019). An Integrative Model of Cellular States, Plasticity, and Genetics for Glioblastoma. Cell *178*, 835-849.e21. https://doi.org/10.1016/j.cell.2019.06.024.

23. Schep, A.N., Wu, B., Buenrostro, J.D., and Greenleaf, W.J. (2017). ChromVAR: Inferring transcription-factor-associated accessibility from single-cell epigenomic data. Nat. Methods *14*, 975–978. https://doi.org/10.1038/nmeth.4401.

24. Păun, O., Tan, Y.X., Patel, H., Strohbuecker, S., Ghanate, A., Cobolli-Gigli, C., Sopena, M.L., Gerontogianni, L., Goldstone, R., Ang, S.L., et al. (2023). Pioneer factor ASCL1 cooperates with the mSWI/SNF complex at distal regulatory elements to regulate human neural differentiation. Genes Dev. *37*, 218–242. https://doi.org/10.1101/gad.350269.122.

25. Panditharatna, E., Marques, J.G., Wang, T., Trissal, M.C., Liu, I., Jiang, L., Beck, A., Groves, A., Dharia, N. V., Li, D., et al. (2022). BAF Complex Maintains Glioma Stem Cells in Pediatric H3K27M Glioma. Cancer Discov. *12*, 2881–2905. https://doi.org/10.1158/2159-8290.CD-21-1491.

26. Mo, Y., Duan, S., Zhang, X., Hua, X., Zhou, H., Wei, H.J., Watanabe, J., McQuillan, N., Su, Z., Gu, W., et al. (2022). Epigenome Programming by H3.3K27M Mutation Creates a Dependence of Pediatric Glioma on SMARCA4. Cancer Discov. *12*, 2906–2929. https://doi.org/10.1158/2159-8290.CD-21-1492.

27. Jessa, S., Mohammadnia, A., Harutyunyan, A.S., Hulswit, M., Varadharajan, S., Lakkis, H., Kabir, N., Bashardanesh, Z., Hébert, S., Faury, D., et al. (2022). K27M in canonical and noncanonical H3 variants occurs in distinct oligodendroglial cell lineages in brain midline gliomas. Nat. Genet. *54*, 1865–1880. https://doi.org/10.1038/s41588-022-01205-w.

28. Marques, C., Unterkircher, T., Kroon, P., Oldrini, B., Izzo, A., Dramaretska, Y., Ferrarese, R., Kling, E., Schnell, O., Nelander, S., et al. (2021). Nf1 regulates mesenchymal glioblastoma plasticity and aggressiveness through the ap-1 transcription factor fosl1. Elife *10*. https://doi.org/10.7554/ELIFE.64846.

29. Yang, Y., Gomez, N., Infarinato, N., Adam, R.C., Sribour, M., Baek, I., Laurin, M., and Fuchs, E. (2023). The pioneer factor SOX9 competes for epigenetic factors to switch stem cell fates. Nat. Cell Biol. *25*, 1185–1195. https://doi.org/10.1038/s41556-023-01184-y.

30. Păun, O., Tan, Y.X., Patel, H., Strohbuecker, S., Ghanate, A., Cobolli-Gigli, C., Sopena, M.L., Gerontogianni, L., Goldstone, R., Ang, S.L., et al. (2023). Pioneer factor ASCL1 cooperates with the mSWI/SNF complex at distal regulatory elements to regulate human neural differentiation. Genes Dev. *37*, 218–242. https://doi.org/10.1101/gad.350269.122.

31. Vierbuchen, T., Ling, E., Cowley, C.J., Couch, C.H., Wang, X., Harmin, D.A., Roberts, C.W.M., and Greenberg, M.E. (2017). AP-1 Transcription Factors and the BAF Complex Mediate Signal-Dependent Enhancer Selection. Mol. Cell *68*, 1067-1082.e12. https://doi.org/10.1016/j.molcel.2017.11.026.

32. Tatavosian, R., Duc, H.N., Huynh, T.N., Fang, D., Schmitt, B., Shi, X., Deng, Y., Phiel, C., Yao, T., Zhang, Z., et al. (2018). Live-cell single-molecule dynamics of PcG proteins imposed by the DIPG H3.3K27M mutation. Nat. Commun. *9*. https://doi.org/10.1038/s41467-018-04455-7.

33. Stafford, J.M., Lee, C.H., Voigt, P., Descostes, N., Saldana-Meyer, R., Yu, J.R., Leroy, G., Oksuz, O., Chapman, J.R., Suarez, F., et al. (2018). Multiple modes of PRC2 inhibition elicit global chromatin alterations in H3K27M pediatric glioma. Sci. Adv. *4*. https://doi.org/10.1126/sciadv.aau5935.

34. Krug, B., De Jay, N., Harutyunyan, A.S., Deshmukh, S., Marchione, D.M., Guilhamon, P., Bertrand, K.C., Mikael, L.G., McConechy, M.K., Chen, C.C.L., et

al. (2019). Pervasive H3K27 Acetylation Leads to ERV Expression and a Therapeutic Vulnerability in H3K27M Gliomas. Cancer Cell *35*, 782-797.e8. https://doi.org/10.1016/j.ccell.2019.04.004.

35. Brien, G.L., Bressan, R.B., Monger, C., Gannon, D., Lagan, E., Doherty, A.M., Healy, E., Neikes, H., Fitzpatrick, D.J., Deevy, O., et al. (2021). Simultaneous disruption of PRC2 and enhancer function underlies histone H3.3-K27M oncogenic activity in human hindbrain neural stem cells. Nat. Genet. *53*, 1221–1232. https://doi.org/10.1038/s41588-021-00897-w.

36. Di Bella, D.J., Carcagno, A.L., Bartolomeu, M.L., Pardi, M.B., Löhr, H., Siegel, N., Hammerschmidt, M., Marín-Burgin, A., and Lanuza, G.M. (2019). Ascl1 Balances Neuronal versus Ependymal Fate in the Spinal Cord Central Canal. Cell Rep. *28*, 2264-2274.e3. https://doi.org/10.1016/j.celrep.2019.07.087.

POINT-BY-POINT RESPONSE LETTER

Round 2

We sincerely thank all the reviewers for their careful evaluation of our manuscript through both rounds of revisions.

Reviewer #1 (Remarks to the Author):

I thank the authors for their response to my initial concerns. The revised manuscript is significantly strengthened by the inclusion of extensive new data, particularly the validation in a second independent diffuse midline glioma cell line (BT245). The authors have successfully addressed most previous limitations. The study now provides a robust mechanistic link between H3K27M, Polycomb-mediated silencing, and ASCL1-driven chromatin remodeling. The evidence supporting ASCL1 as a critical mediator of tumor state diversity and a potential therapeutic target is compelling. The authors have met most of my requirements. This manuscript now provides valuable insights into the molecular drivers of DMG plasticity. I recommend this work for publication.

Summary of Improvements

Citations Integrated Lewis et al. (2022) and 15+ new 2024-2025 references. Resolved Model Diversity Full epigenomic and functional validation in BT245 line. Resolved Cell Characterization Provided transcript abundance (68% mutant) and genomic history. Resolved
In Vivo Evidence Added clonal ASCL1-KO xenograft data showing abrogated tumorigenesis. Resolved

We thank the reviewer for their constructive comments and suggestions, including further characterization of a second DMG cell line, which have been valuable in improving our manuscript.

Reviewer #2 (Remarks to the Author):

Overall, the authors have provided a robust point-by-point response to all comments. The experimental effort undertaken during the revision is substantial, with characterization of a second independent cell line (BT245), including phenotypic plasticity assays, single-nuclei omics, and new xenograft experiments using validated clonal ASCL1-KO lines; addition of a patient survival analysis; ASCL1 rescue experiments; and adoption of a common reference framework (Neftel et al., 2019) for the definition of glioma cell states.

A few points remain partially unresolved at the experimental level (ASCL1, SOX factors, and BRG1 interactions), but these limitations are explicitly acknowledged by the authors and discussed appropriately. They do not undermine the central conclusions of the manuscript, which are robust and of high interest.

I recommend acceptance of this manuscript.

We thank the reviewer for their constructive comments and suggestions, in particular the single-cell RNA-seq experiment to further characterize transcriptional heterogeneity in DMGs, which we believe has significantly improved our manuscript.

Reviewer #3 (Remarks to the Author):

The authors have satisfactorily addressed most of the concerns raised in my initial review. The manuscript has been strengthened by the inclusion of additional analyses that clarify the transcriptional programs associated with ASCL1, improved gene-level visualization of cell state–associated markers, and the incorporation of an additional DMG model (BT245) with supporting single-cell and in vivo validation experiments. Together, these additions improve the clarity of the presentation and strengthen the overall conclusions of the study.

One point that may be helpful to acknowledge in the discussion is that the primary experimental systems used in this work rely largely on patient-derived DMG cell lines maintained under in vitro culture conditions. While these models preserve key genetic features of H3K27M DMG and demonstrate tumorigenic capacity in xenograft settings, prolonged culture and differentiation paradigms may not fully capture the cellular heterogeneity or tumor microenvironment present in primary tumors. Notably, it is possible that other neurodevelopmental transcription factors or regulatory pathways could interact with or compensate for ASCL1 in more physiological contexts beyond SOX and BRG1.

We have added the following sentence in the discussion: "Finally, we note that *in vitro* differentiation and culture conditions may not fully recapitulate the cellular heterogeneity present in primary tumors, and ASCL1 may functionally interact with additional factors beyond SOX and BRG1 *in vivo*."

We thank the reviewer for their constructive comments and suggestions. We believe highlighting key individual genes and loci was valuable in further supporting our conclusions and improving our manuscript.

Reviewer #4 (Remarks to the Author):

The authors have adequately addressed my comments. Thank you.

We thank the reviewer for their constructive comments and suggestions, including the rescue experiment and the addition of single-cell multiomics data, which we believe have significantly improved our manuscript.
